# A Set of Functional Brain Networks for the Comprehensive Evaluation of Human Characteristics

**DOI:** 10.3389/fnins.2018.00149

**Published:** 2018-03-14

**Authors:** Yul-Wan Sung, Yousuke Kawachi, Uk-Su Choi, Daehun Kang, Chihiro Abe, Yuki Otomo, Seiji Ogawa

**Affiliations:** ^1^Kansei Fukushi Research Institute, Tohoku Fukushi University, Sendai, Japan; ^2^Neuroscience Research Institute, Gachon University, Incheon, South Korea

**Keywords:** resting-state fMRI, functional network, neuronal plasticity, human characteristics, psychometric parameters

## Abstract

Many human characteristics must be evaluated to comprehensively understand an individual, and measurements of the corresponding cognition/behavior are required. Brain imaging by functional MRI (fMRI) has been widely used to examine brain function related to human cognition/behavior. However, few aspects of cognition/behavior of individuals or experimental groups can be examined through task-based fMRI. Recently, resting state fMRI (rs-fMRI) signals have been shown to represent functional infrastructure in the brain that is highly involved in processing information related to cognition/behavior. Using rs-fMRI may allow diverse information about the brain through a single MRI scan to be obtained, as rs-fMRI does not require stimulus tasks. In this study, we attempted to identify a set of functional networks representing cognition/behavior that are related to a wide variety of human characteristics and to evaluate these characteristics using rs-fMRI data. If possible, these findings would support the potential of rs-fMRI to provide diverse information about the brain. We used resting-state fMRI and a set of 130 psychometric parameters that cover most human characteristics, including those related to intelligence and emotional quotients and social ability/skill. We identified 163 brain regions by VBM analysis using regression analysis with 130 psychometric parameters. Next, using a 163 × 163 correlation matrix, we identified functional networks related to 111 of the 130 psychometric parameters. Finally, we made an 8-class support vector machine classifiers corresponding to these 111 functional networks. Our results demonstrate that rs-fMRI signals contain intrinsic information about brain function related to cognition/behaviors and that this set of 111 networks/classifiers can be used to comprehensively evaluate human characteristics.

## Introduction

Humans exhibit diverse characteristics of emotion, cognition, and behavior that describe individuals. Many different psychometric parameters from questionnaires or behavioral tasks have been developed to evaluate human characteristics of cognition/behavior (Diener et al., 1985; Raine, 1991; Carver and White, 1994; Yamauchi et al., 2009). Brain function underlies cognition/behaviors, and thus, it is possible that the characteristics of an individual can be evaluated by measuring brain function.

Functional MRI (fMRI) is the most widely used non-invasive method of measuring human brain function (Ogawa et al., 1992; Kim and Ugurbil, 1997). Measurements of human cognition/behavior by fMRI require psychometric parameters describing cognition/behavior that are embodied as tasks to induce neuronal processing in the brain (brain activation). Some psychometric parameters are easy to formalize, whereas others are not (Rupp and Zumbo, 2006). For the former type of parameters, a task can be established to evoke brain activation, and the corresponding fMRI signals of brain activation can be detected from the relevant brain areas.

Brain imaging by task-based fMRI (tb-fMRI) has provided information about the brain areas and brain networks that represent given tasks (Poldrack et al., 2013). However, most brain imaging studies have focused on specific psychometric parameters (Gauthier et al., 2000; Grill-Spector et al., 2004; Fernández-Alcántara et al., 2016; Krendl and Kensinger, 2016; Kogler et al., 2017) rather than considering a comprehensive set of psychometric parameters that can describe the diverse human characteristics of cognition/behavior. This is because it is very difficult to identify brain areas/networks responsible for the many diverse human characteristics due to limitations in task designs and time to perform tests.

In contrast to tb-fMRI, resting-state fMRI (rs-fMRI) signals come from intrinsic brain activities not designated to explicit tasks (Fransson, 2005; Fox and Raichle, 2007), and thus, there is no information to link a measured rs-fMRI signal with a specific brain function. However, functional networks produced from the correlation of rs-fMRI signals with certain brain areas, such as the default mode network, are known to represent various aspects of brain function (Greicius et al., 2004; Mason et al., 2007). Recent studies have further shown that task performance can be estimated by brain networks identified by rs-fMRI (Tavor et al., 2016; Craig et al., 2017; Song et al., 2017). In addition, some studies have proven that brain areas or functional connectivity in a rs-fMRI network are correlated with the scores of an explicit task used to identify functional areas by tb-fMRI (Finn et al., 2015; Meskaldji et al., 2016). These previous studies suggested that rs-fMRI signals can be used to identify brain networks that represent sensory and higher-order cognitive function or higher-order social function (Finn et al., 2015; Lei et al., 2017; Yang et al., 2017). That is, rs-fMRI signals may represent functional infrastructures for processing information in the brain that is highly involved in the brain function related to cognition/behavior. These characteristics of rs-fMRI may allow diverse information about the brain to be obtained from rs-fMRI signals acquired during a single MRI scan if appropriate supporting information is provided that can explain the characteristics of rs-fMRI signals or the correlation between brain areas based on the signals.

**Figure 1:**
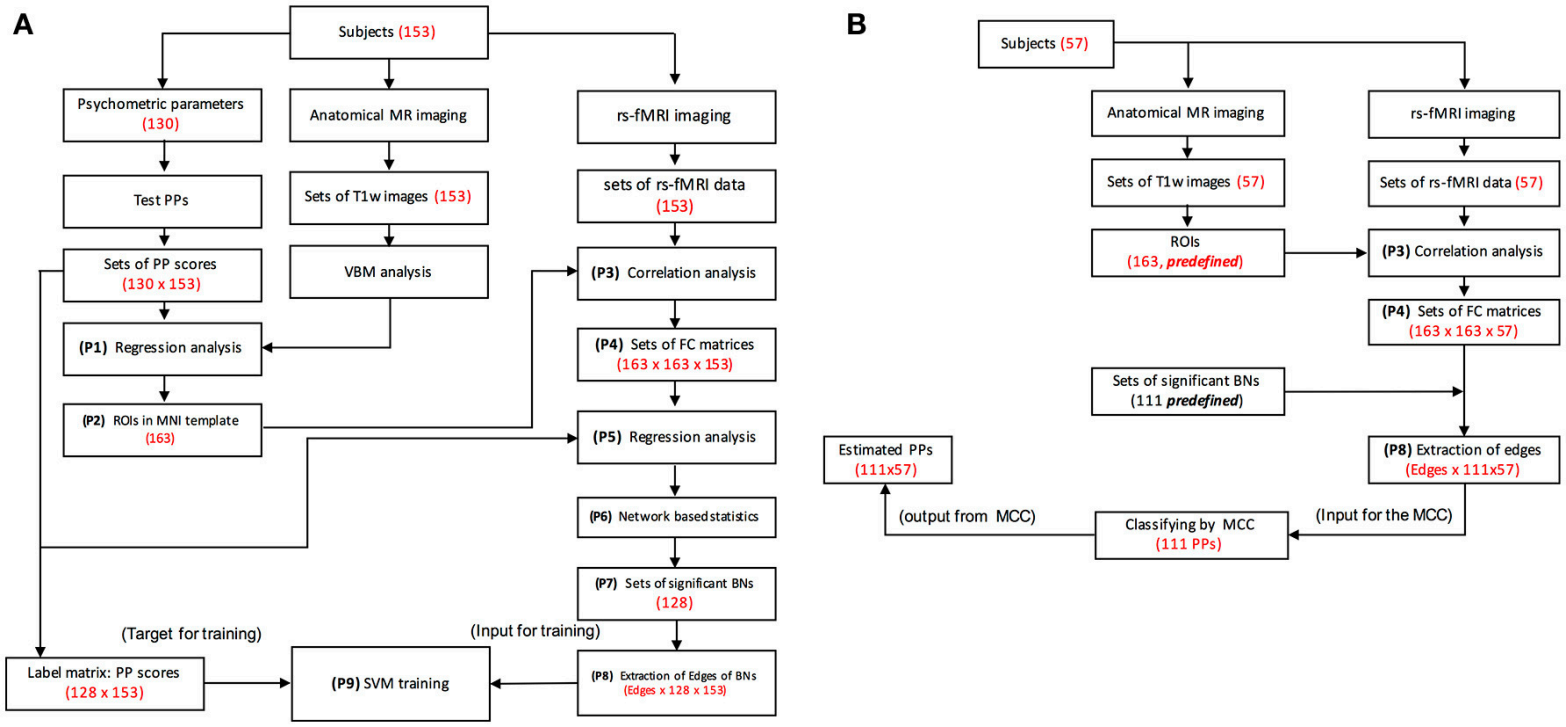
Procedure for MRI data processing. P1, P2, etc., stand for processing steps. **(A)** Diagrams of data flow for the primary experiment. Brain areas are identified by psychometric parameters and MRI data by regression analysis (P1, P2). fMRI signals are extracted from the ROIs, and the correlation matrix is constructed (P3, P4). Regression analysis and network based statistics (NBS) analysis were performed for the correlation matrices of 153 subjects (134 for IQ and 123 for EQ), and significant brain networks are identified (P5, P6, P8). Inputs to the SVM classifiers are determined from the edges of the identified functional networks (P7, P8). Scores of psychometric parameters are estimated by classifiers and compared with psychologically measured scores to calculate accuracy. Numbers and letters in the parenthesis stand for specific information obtained after the processing at each step. PP, psychometric parameter; SVM, support vector machine; rs-Fmri, resting-state functional MRI; FC, functional connectivity; BN, brain network; MCC, multiple class classifiers; T1w, T1 weighted. **(B)** Diagrams of data flow for the supplementary experiment. Bypassing the regression analyses used during training, fMRI signals are extracted from the ROIs, and the edges of functional networks are calculated (P3, P4, P8) for input into the SVM classifiers (P9). Scores of psychometric parameters are estimated by the classifiers.

Using behavioral data about psychometric parameters as the supporting information, we attempted to test whether functional networks of the brain could be identified by rs-fMRI signals and psychological parameters (Figure 1). Recently some studies have shown that it is possible to decode brain states from fMRI responses using machine learning algorithms, such as a support vector machine (Guo et al., 2015; Altmann et al., 2016; Hu et al., 2017; Zafar et al., 2017). To verify that the functional networks identified represent the corresponding psychological parameters, and also to evaluate human characteristics from the rs-fMRI signals, we used a support vector machine (SVM).

We attempted to derive a specific SVM classifier related to each identified functional network. Second, we aimed to identify a set of functional networks of the brain to comprehensively evaluate human characteristics (Figure 1). For this purpose, we prepared a set of 130 psychometric parameters (Table 1; Supplementary References), including those related to intelligence quotient (IQ), emotional quotient (EQ), and social ability/skill that can explain the most conceivable aspects of human cognition/behavior by which an individual can be described.

**Table 1:** List of psychometric parameters.

	**Parameters**
1	Cognitive competence
2	Extracurricular competence including physical competence
3	Social competence with friends of the same sex
4	Social competence with friends of opposite sex
5	General self-worth
6	**Perceived competence scale for adolescence**
7	Inhibitory control
8	Activation control
9	Attentional control
10	**Japanese version of Effortful Control (EC) scale for adults**
11	Trust vs. mistrust
12	Autonomy vs. shame/doubt
13	Initiative vs. guilt
14	Industry vs. inferiority
15	Identity vs. role confusion
16	Intimacy vs. isolation
17	**Japanese version of Rasmussen's Ego Identity Scale (REIS)**
18	Behavioral inhibition system (BIS)
19	BAS/driver
20	BAS/reward
21	BAS/fun seeking
22	**Behavioral Inhibitory System (BIS)/Behavioral Activate System (BAS) scale**
23	**Rosenberg Self Esteem Scale (RSES)**
24	Anxiety regarding others' evaluation of oneself and perceived maladjustment to interpersonal situations
25	Emotional disturbance
26	Difficulty in expressing opinions
27	**Japanese version of Jones and Russell's social reticence scale for college students**
28	Beginning social skills
29	Advanced social skills
30	Skills for dealing with feelings
31	Skill alternatives to aggression
32	Skills for dealing with stress
33	Planning skills
34	**Kikuchi's Scale of Social Skills (KiSS-18)**
35	Public self-consciousness
36	Private self-consciousness
37	**Self-consciousness scale for Japanese**
38	**Japanese version of the Self-Concept Clarity (SCC) scale**
39	Self-continuity function subscale
40	Directing-behavior function subscale
41	Social-bonding function subscale
42	**Japanese version of the TALE (Thinking About Life Experiences) scale**
43	**Japanese version of the Ego-Resiliency Scale (ER89)**
44	Positive Problem Orientation (PPO)
45	Negative Problem Orientation (NPO)
46	Problem Definition and Formulation (PDF)
47	Generation of Alternative Solution (GAS)
48	Decision Making (DM)
49	Solution Implementation and Verification (SIV)
50	Rational Problem Solving (RPS)
51	Impulsivity/Carelessness Style (ICS)
52	Avoidance Style (AS)
53	**Japanese version of the Social Problem-Solving Inventory-Revised (SPSI-R)**
54	Negative-Self (NS)
55	Positive-Self (PS)
56	Negative-Other (NO)
57	Positive-Other (PO)
58	**Japanese version of the Brief Core Schema Scale (JBCSS)**
59	Intentional behavior
60	Planfulness
61	Readiness for change
62	Using resource
63	**Japanese version of the Personal Growth Initiative Scale-II (PGIS-II)**
64	**Subjective Happiness Scale (SHS)**
65	**The Satisfaction with Life Scale (SWLS)**
66	State Anxiety (A-State)
67	Trait Anxiety (A-Trait)
68	**Japanese edition of state-trait anxiety inventory**
69	Positive symptoms
70	Negative symptoms
71	Disorganization
72	**Schizotypal Personality Questionnaire (SPQ)**
73	Social skill
74	Attention switching
75	Attention to detail
76	Communication
77	Imagination
78	**Autism-spectrum quotient**
79	**Verbal Intelligence Quotient (VIQ)**
80	**Performance Intelligence Quotient (PIQ)**
81	**Full scale Intelligence Quotient (FIQ)**
82	**Fluid intelligence**
83	**Crystallized intelligence**
84	Verbal comprehension
85	Perceptual organization
86	Vocabulary
87	Similarities
88	Arithmetic
89	Digit span
90	Information
91	Comprehension
92	Picture completion
93	Digit symbol
94	Block design
95	Matrix reasoning
96	Picture arrangement
97	Emotional awareness
98	Self-efficacy
99	Perseverance
100	Enthusiasm
101	Self-decision
102	Impulse control
103	Patience
104	Sharing positive emotion
105	Sharing negative emotion
106	Personal consideration
107	Voluntary support
108	Personal management
109	Sociability
110	Cooperation
111	Decision making
112	Optimism
113	Group consideration
114	Influence
115	Risk management
116	Tactfulness
117	Adaptability
118	**Self-awareness**
119	**Self-motivation**
120	**Self-control**
121	**Empathy**
122	**Altruism**
123	**Interpersonal relationship**
124	**Situational awareness**
125	**Leadership**
126	**Flexibility**
127	**Intrapersonal Emotional Quotient Scale (EQS)**
128	**Interpersonal (EQS)**
129	**Situational (EQS)**
130	**EQS total scale**

*Social ability/skill: 1-78, IQ: 79-96, EQ: 97-130*.

## Materials and methods

The experiments in this study comprised a primary experiment and a supplementary experiment. The primary experiment was designed to identify functional networks and to design the SVM classifiers. The supplementary experiment was performed to test the SVM classifiers developed in the primary experiment.

### Measurements of psychometric parameters

In the primary experiment, psychometric parameters were measured on three separate days: one day for the Wechsler Adult Intelligence Scale (WAIS; a measure of IQ), one day for the Emotional Quotient Scale (EQS) (Uchiyama et al., 2001), and one day for the other psychometric parameters including those related to social ability/skill. The psychometric parameters related to IQ consisted of 11 subset scores, 5 IQ scores, and 2 sub-indices derived from the subset scores. The psychometric parameters related to EQS consisted of 21 sub-factors, 12 major factors derived from the sub-factors, and a total score. The other psychometric parameters consisted of 59 sub-factors and 19 major factors, some of which were derived from the sub-factors. The parameters are listed in Table 1. Major scores and factors appear in bold type. The total number of parameters including major parameters and sub-parameters was 130. To the best of our knowledge, the number of parameters in this study is greater than in any previous psychological study. This set of 130 parameters covers most conceivable human characteristics and is considered to provide sufficient information to complete a comprehensive evaluation of human characteristics, therefore, all 130 parameters were used for the identification of functional networks.

### MRI measurements

For both the primary experiment and the supplementary experiment, each subject underwent an MRI using a 3-Tesla MRI scanner (Skyra-fit; Siemens Co., Erlangen, Germany). All subjects were scanned in two sessions corresponding to a structural image (T1) measurement for VBM and a functional image measurement (resting-state fMRI). Structural images were acquired using the following parameters: repetition time = 1,900 ms, echo time = 2.52 ms, matrix size = 256 × 256, in-plane resolution = 1 × 1 mm^2^, slice thickness = 1 mm, and number of slices = 192. The imaging orientation was sagittal. Resting-state fMRI data were acquired using the following parameters: repetition time = 1,000 ms, echo time = 24 ms, matrix size = 64 × 64, in-plane resolution = 3.4 × 3.4 mm^2^, slice thickness = 3.4 mm, and number of volumes = 480. In the resting-state fMRI session, subjects were asked to lie on the bed and not to wander their mind with their eyes open and to gently focus their eyes on the center of the visual field. The lights in the room were turned off during all MRI scans.

### Participants

#### Primary experiment

A group of 162 participants consisting of university freshmen and sophomores [55 males, 107 females; mean age and standard deviation (SD) 20.10 ± 0.86 years; 26 left hand, 136 right hand] participated in both psychometric measurements and underwent MRI as part of the primary experiment. The aim of the primary experiment was to identify functional networks and to derive the classifiers.

#### Supplementary experiment

In the supplementary experiment to test the networks and classifiers obtained in the primary experiment, a group of 57 participants (10 males, 47 females; mean age and *SD* 21.97 ± 1.47 years) participated in the psychometric measurements (parameters related to social ability/skill; refer to Measurements of Psychometric Parameters) and MRI measurements.

None of the participants had a history of neurological disease or any medical condition (i.e., pregnancy, use of a cardiac pacemaker, or claustrophobia). After the participants had been provided a complete description of the study, written informed consent was obtained in accordance with the Declaration of Helsinki. This study was approved by the Institutional Review Board Tohoku Fukushi University (Japan), and all experiments were performed with the relevant guidelines and regulations of the Institutional Review Board, which approved all the experimental protocols.

### MRI data analysis: primary experiment

The analysis of the primary experiment was performed through several processing steps for 153 participants after the removal of participants with motion artifacts (*n* = 9) during MRI acquisition (Figure 1A).

### VBM analysis

For identification of functional networks anatomical brain templates such as automated anatomical labeling (AAL) atlas (Yahata et al., 2016) have been used. To avoid partial volume effect and improve sensitivity, in this study, we tried to identify brain areas specific to psychometric parameters in each of gyri in AAL atlas. MRI data were analyzed using VBM8 (http://dbm.neuro.uni-jena.de/vbm/) implemented in the Statistical Parametric Mapping software (SPM 12; Wellcome Department of Imaging Neuroscience, London, United Kingdom), which was implemented via Matlab 2015a (MathWorks, Sherborn, MA, USA). The pre-processing by VBM8, with the default parameters used in each step, included segmentation with a bias-corrected option and spatial normalization with diffeomorphic anatomical registration using exponentiated Lie algebra (DARTEL) (Ashburner, 2007), normalization to the Montreal Neurological Institute and Hospital (MNI) coordinate system with smoothing with an 8-mm full-width at half-maximum (FWHM) filter, and modulation by Jacobian determinants, which provided segmented, normalized, and modulated gray matter images. After preprocessing, a statistical test (simple regression analysis) was used to identify brain areas correlated with each psychometric parameter (P1 and P2 of Figure 1A). In the regression analysis, total brain value (TBV) was used as a covariate. Our purpose was to find specific brain areas in AAL gyri we performed a small volume correction in each gyrus based on the AAL atlas to correct for multiple comparisons (Gur et al., 2000; Singh et al., 2014). The statistical threshold was set at an uncorrected *p* = 0.001 and at *p* = 0.05 for family-wise error (FWE) correction at the voxel level.

### rs-fMRI analysis

For functional MRI data, we used the data processing assistant for resting-state fMRI software (DPABI) (Chao-Gan and Yu-Feng, 2010; Yan et al., 2016) to pre-process the data of all 153 subjects. The pre-processing included slice-scan time correction, 3D motion correction (maximum head motion: 151 subjects passed the maximum threshold of 1.5 mm and 1.5°, and other two subjects passed the maximum threshold of 3.0 mm and 3°), removal of head motion effects by the Friston 24-parameter model, bandpass temporal filtering (between 0.01 and 0.1 Hz), and artifact rejection based on the CSF signal. These functional images were smoothed with a 6-mm FWHM filter and co-registered with each corresponding structural image. All resting-state functional images were analyzed by ROI-based correlation analysis for the predefined 163 ROIs identified by VBM (P3 of Figure 1A). In the analysis, for the time courses of the 163 ROIs, correlations were calculated, and correlation coefficient matrices (163 × 163 × 153) were created (P4 of Figure 1A). A simple regression of the correlation coefficient matrices was performed with psychologically measured scores of the psychometric parameters, and then network-based statistics (NBS) were performed to identify sets of significant brain networks (i.e., a brain network for each psychometric parameter). Zalesky et al. (2010) (P6 of Figure 1A) to identify sets of significant brain networks (mostly a brain networks for each psychometric parameter; P7 of Figure 1A). In this step, randomly shuffled correlation matrices were generated for a permutation test. Then, correlational analyses were performed on the true correlation matrix with psychometric parameters, and a non-parametric permutation test was performed on random correlation matrices with 5,000 iterations. Correction for multiple comparisons based on NBS statistics was performed at an initial threshold of *p* < 0.005, and the number of edges in the network was limited to as close to 15 as possible. Previous research has shown that a small number of brain connections can predict a psychiatric disorder (Yahata et al., 2016).

### SVM analysis

#### Classifier design

SVMs are typically used to identify a hyperplane that distinguishes between 2 groups. But in this study we attempted to design 8-class SVM classifiers. Behavioral scores are measured on a scale of 1–100. Our purpose is ideally to measure psychometric parameters by MRI at the same scale. However, there are several limitations such as sensitivity and number of samples. So, we reduced it to a scale of 1–10, 10 levels. But in classification study such as the neural network, the number of levels should be a power of 2. Therefore we decided to use 8 (i.e., 2^3^) classes. The 8-class SVM classifier can estimate the score of a psychometric parameter from rs-fMRI data into 8 levels. Although psychometric parameter scores are linear and some regression analyses such as support vector regression can be applied, we attempted to design the SVM classifiers because a discretization is generally stable to variations and some variations would exist in fMRI and psychometric measurements.

Multiple class classification with the one-against-all (OAA) method (Bishop, 2007) was performed using LIBSVM (Chang and Lin, 2011), which was implemented in Matlab 2015a (MathWorks, Sherborn, MA, USA). The OAA method involved two steps: training a single classifier per class with one positive sample of each class and all other negative samples of the remaining classes and repeating the procedure for the total number of classes. The classifier with the highest classification score was selected as a final multi-class classifier.

#### Data preparation for training—classification

Data sets for the SVM classifier design were prepared from the results of the brain network analysis and the psychologically measured data. The input dataset consisted of data matrices of features (P8 of Figure 1A) and labels (P9 of Figure 1A). A feature data matrix consisted of edges of a network (correlation coefficients of networks at P8 of Figure 1A) and a label matrix consisted of psychologically measured data (P9 of Figure 1A). For classification label data (the label matrix), the scores of psychologically measured psychometric parameters were normalized and divided into eight classes by the equation {(x-min)/(max-min)} ^*^ 8, where × is the score of the psychologically measured psychometric parameter. In the calculation results, the number after the decimal point was rounded up. The dimensions of a feature matrix were the number of edges by the number of subjects, and the number of feature matrices was the number of functional networks. Therefore, the dimensions of the input data set for the SVM were the number of edges by the number of functional networks by the number of subjects (the number of edges were omitted in Figure 1B for simplification). The dimensions of the label matrices were the number of functional networks (scores of psychologically measured psychometric parameters rated into one of eight classes) by the number of subjects.

#### Training of classifiers

With the data sets of all psychometric parameters, a leave-one-out cross validation was run using a radial basis function (RBF) kernel defined as K (x, y) = exp (−γ ∥ x − y ∥ 2) for optimizing the C and gamma (γ) values. In parameter optimization, the grid-search method was performed with various ranges of C and γ values (C = 2^−5^, 2^−4^, …, 2^15^, γ = 2^−15^, 2^−14^, …, 2^15^). After cross validation, the multiple class classifiers of the psychometric parameters with the best C and γ values were varied and deemed significant (P7 of Figure 1A).

### Procedure for MRI data: supplementary experiment

#### Test of classifiers

In the supplementary experiment, the analyses were performed on 57 participants to test the classifiers. MRI data were preprocessed via the same procedures as in the primary experiment but bypassing the regression analyses in the training procedure. fMRI signals were extracted from the ROIs, the edges of the functional networks were calculated (P3, P4, P8), and inputs for the SVM classifiers were determined from the edges (P8). Scores of psychometric parameters were estimated by the trained classifiers (Figure 1B). The estimated scores were compared with the psychologically measured scores to calculate the accuracy.

### Summary of the data processing procedures

#### Training procedure Figure 1A

Brain areas were identified by the psychometric parameters and the MRI data via a regressing analysis (P1, P2). From the ROIs, fMRI signals were extracted, and raw correlation matrices were constructed (P3, P4). A regression was performed and then a network-based statistics (NBS) analysis was performed, to identify significant brain networks (P5, P6, P7). The inputs for the SVM classifiers were from edges of the identified functional networks (P8). Scores for the psychometric parameters were estimated using a scale from 1 to 8 by the SVM classifiers and compared with psychologically measured scores, which were also rescaled to 8 levels, to calculate accuracy.

#### Test procedure Figure 1B

Bypassing the regression analyses in the training, fMRI signals were extracted from the ROIs, and then the edges of functional networks were calculated (P3, P4, P7, P8). The extracted edges were used for the inputs for the SVM classifiers and the scores of psychometric parameters were estimated by the classifiers.

## Results

### Psychological data

Scores for the psychometric parameters were acquired for all 153 participants (153 for social ability, 134 for IQ, and 123 for EQ; as data were acquired over several days, IQ and EQ data could not be acquired for some participants) included in the primary experiment (see the Materials and Methods section) and are listed in the order of the MRI measurements performed. We used all 130 parameters for subsequent MRI processing to map each parameter to the brain, such that each functional network to be identified would have sufficient information to evaluate the psychometric parameters. The behavioral scores for all 130 parameters are listed in Table 2 for the participants in the primary experiment.

**Table 2:** Behavioral scores of 130 psychometric parameters (mean and standard deviation).

	***N***	**Mean**	***SD***
1	153	58.38	10.40
2	153	56.70	14.66
3	153	73.22	12.25
4	153	56.55	20.05
5	153	56.90	12.00
6	153	60.16	9.49
7	153	66.71	9.19
8	153	64.31	10.45
9	153	58.48	10.66
10	153	63.07	8.23
11	153	56.52	10.08
12	153	60.33	11.32
13	153	45.61	9.88
14	153	57.34	10.67
15	153	53.84	10.33
16	153	51.52	12.06
17	153	54.07	7.82
18	153	69.56	8.21
19	153	80.72	10.30
20	153	59.97	17.37
21	153	77.61	16.35
22	153	73.24	8.27
23	153	59.43	12.00
24	153	54.17	18.22
25	153	71.53	17.56
26	153	56.14	21.67
27	153	58.61	15.17
28	153	63.09	18.37
29	153	66.10	12.82
30	153	63.05	14.43
31	153	62.53	13.33
32	153	64.92	14.59
33	153	64.58	14.40
34	153	64.05	10.74
35	153	73.36	11.74
36	153	65.16	12.04
37	153	69.45	10.02
38	153	57.31	10.42
39	153	60.87	16.47
40	153	67.58	15.26
41	153	69.35	16.33
42	153	65.51	12.63
43	153	68.43	10.48
44	153	58.10	16.82
45	153	56.06	19.76
46	153	63.17	14.36
47	153	53.69	16.09
48	153	58.99	15.68
49	153	50.82	18.65
50	153	56.67	13.09
51	153	47.57	14.03
52	153	44.72	16.73
53	153	52.09	11.44
54	153	25.41	17.65
55	153	73.67	16.07
56	153	11.47	13.51
57	153	63.94	16.52
58	153	43.62	11.00
59	153	64.35	13.66
60	153	56.49	13.34
61	153	53.35	13.42
62	153	59.66	14.68
63	153	58.27	11.03
64	153	66.34	13.87
65	153	50.16	14.18
66	153	72.17	10.21
67	152	63.15	10.08
68	152	67.63	8.93
69	153	26.31	20.39
70	153	52.70	23.65
71	153	43.90	26.40
72	153	40.70	17.61
73	153	40.13	28.42
74	153	53.79	18.74
75	153	46.14	24.31
76	153	40.13	21.18
77	153	31.90	15.93
78	153	42.42	13.50
79	137	103.90	10.06
80	137	98.79	11.60
81	137	101.77	10.08
82	137	126.73	13.77
83	137	101.36	13.80
84	137	104.18	10.30
85	137	97.94	13.82
86	137	43.60	10.01
87	137	65.89	11.17
88	137	58.90	11.66
89	137	63.92	11.14
90	137	57.80	11.80
91	137	57.49	13.25
92	137	65.33	9.98
93	137	71.10	10.66
94	137	76.60	13.60
95	137	78.66	10.78
96	137	77.87	10.92
97	123	57.18	20.34
98	123	47.09	20.72
99	123	58.54	20.37
100	123	52.98	17.89
101	123	49.73	21.76
102	123	57.52	22.77
103	123	44.11	18.44
104	123	76.08	21.63
105	123	58.74	22.28
106	123	68.02	24.48
107	123	63.41	21.33
108	123	37.74	21.53
109	123	43.63	24.05
110	123	55.56	19.84
111	123	42.07	23.59
112	123	42.07	22.55
113	123	66.19	18.98
114	123	36.31	26.53
115	123	42.41	22.86
116	123	47.15	21.89
117	123	53.59	22.66
118	123	52.13	18.82
119	123	55.76	17.52
120	123	50.45	16.42
121	123	67.41	20.41
122	123	65.72	19.59
123	123	45.64	18.62
124	123	50.11	17.51
125	123	39.36	23.08
126	123	50.37	20.81
127	123	52.45	14.82
128	123	57.60	16.26
129	123	47.12	18.36
130	123	52.39	14.61

### Voxel-based morphometry

**Figure 2:**
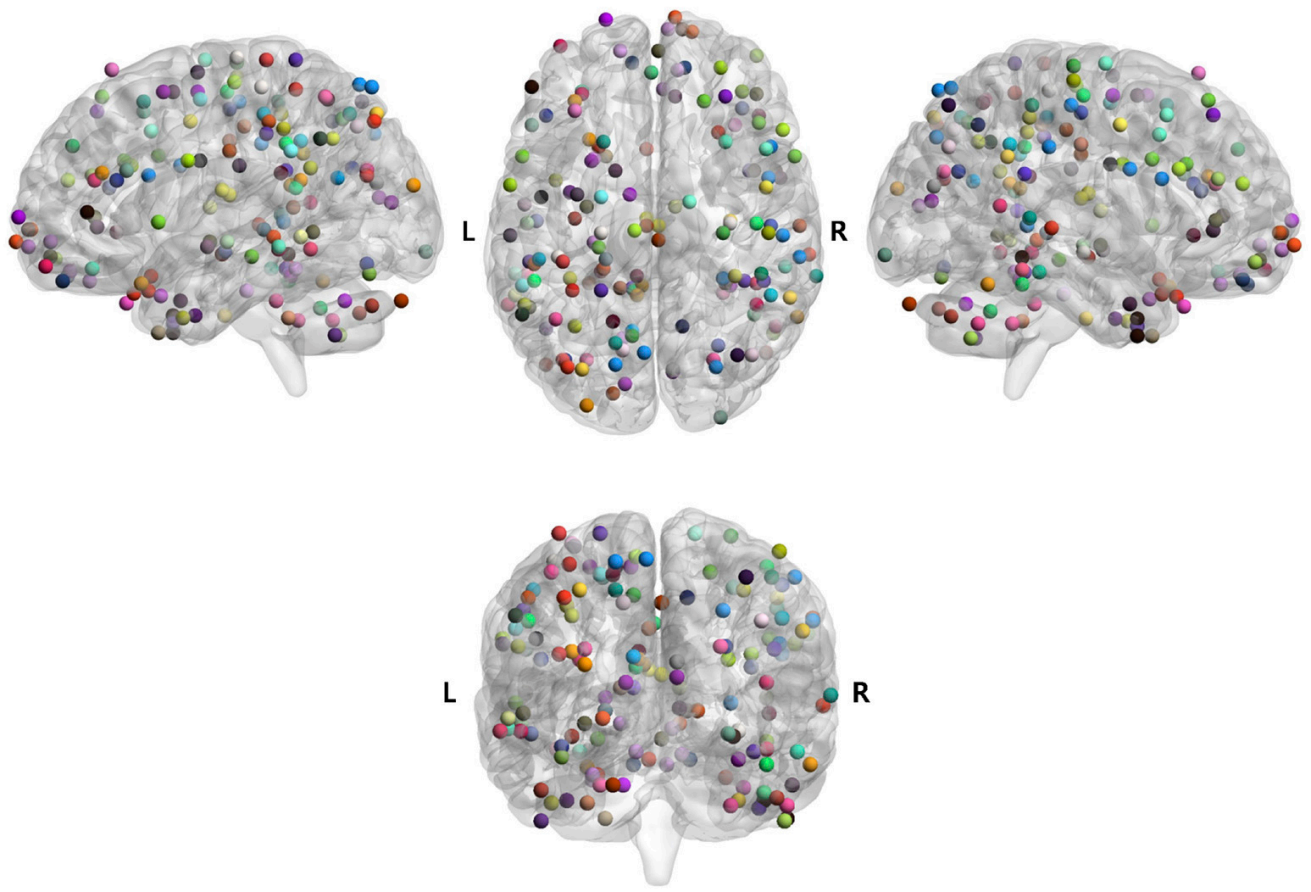
Brain maps of 163 regions of interest (ROIs) for 96 psychometric parameters. Those ROIs were identified by the regression analysis (P1 and P2 of Figure 1A). The psychometric parameters corresponding to the ROIs are shown in Table S1.

Brain areas that were significantly correlated with each psychometric parameter, reflecting a specific function of cognition/behavior related to the parameter, were identified by correlation analysis of the VBM data with the score of each psychometric parameter (P1 of Figure 1A). Brain areas for 96 parameters among 130 total psychometric parameters were identified by the analysis (*p* = 0.05, corrected; see section Materials and Methods for more details), which corresponded to a total of 187 brain areas, as some parameters were correlated with multiple brain areas. An ROI was defined as a 5-mm-diameter sphere around the voxel corresponding to the peak *t*-value in each of the 187 brain areas. Among the 187 ROIs, some ROIs overlapped with others. Therefore, the overlapping ROIs, except the first overlapping ROI in the parameter list, were removed, leaving a total of 163 remaining ROIs (P2 of Figure 1A). The locations of the ROIs in the brain are shown in Figure 2, and their coordinates are given in Table 3. The ROIs were distributed over the frontal, parietal, temporal, occipital, and cerebellar cortices and sub-cortices of the brain.

**Table 3:** Description of 163 regions of interest and the coordinates of the regions in MNI coordinates.

**#**	**Region**	**MNI coordinates**			**Psychometric parameters**
		***x***	***y***	***z***	
1	MFG_R	31	18	48	Cognitive competence
2	SFG_R	16	−7	69	Cognitive competence
3	MFG_ope_R	48	15	40	Cognitive competence
4	MFG_ope_L	−29	39	−16	Cognitive competence
5	MTG_L	−57	−37	−7	Cognitive competence
6	IPG_L	−49	−40	35	Social competence with friends of the same sex
7	PoCG_R	44	−16	56	Social competence with friends of the same sex
8	FG_R	44	−41	−21	Social competence with friends of the same sex
9	FG_L	−36	−74	−18	Social competence with friends of opposite sex
10	IPG_R	50	−46	36	General self-worth
11	PoCG_R	50	−17	49	Perceived competence scale for adolescence
12	IFG_orb_L	−38	58	−15	Inhibitory control
13	MFG_L	−29	38	20	Inhibitory control
14	MTG_R	44	−50	11	Inhibitory control
15	MTG_L	−52	−37	−8	Inhibitory control
16	MFG_L	−32	35	23	Activation control
17	MOG_L	−27	−92	18	Activation control
18	IFG_orb_L	−26	19	−22	Activation control
19	AG_L	−32	−59	37	Attentional control
20	SPG_L	−15	−66	47	Japanese version of Effortful Control (EC) scale for adults
21	MTG_R	69	−38	6	Japanese version of Effortful Control (EC) scale for adults
22	SPG_L	−15	−75	58	Trust vs. mistrust
23	SPG_L	−3	−70	59	Trust vs. mistrust
24	SMG_R	62	−30	35	Trust vs. mistrust
25	FG_R	33	−42	−19	Intimacy vs. isolation
26	Calc_L	−11	−83	11	Intimacy vs. Isolation
27	Calc_R	9	−78	13	Intimacy vs. isolation
28	MFG_L	−25	10	56	Intimacy vs. isolation
29	Hipp_L	−20	−35	−3	Japanese version of Rasmussen's Ego Identity Scale (REIS)
30	IFG_orb_R	10	70	−5	Japanese version of Rasmussen's Ego Identity Scale (REIS)
31	PrCG_R	48	−1	44	Behavioral Inhibition System (BIS)
32	SPG_L	−8	−63	45	BAS/driver
33	PrCG_R	30	−20	68	BAS/driver
34	Cu_L	−8	−61	21	BAS/reward
35	TP_Inf_R	41	6	−38	BAS/fun seeking
36	SPG_L	−21	−44	69	BAS/fun seeking
37	IPG_L	−51	−32	39	BAS/fun seeking
38	FG_R	39	−40	−16	BAS/fun seeking
39	SFG_orb_R	13	65	0	Rosenberg Self Esteem Scale (RSES)
40	Hipp_R	19	−31	0	Difficulty in expressing opinions
41	Cu_R	9	−77	19	Japanese version of Jones and Russell's social reticence scale for college students
42	PoCG_L	−37	−32	69	Beginning social skills
43	MOG_L	−37	−73	22	Beginning social skills
44	SFG_L	−21	−5	53	Advanced social skills
45	SMG_L	−54	−42	32	Advanced social skills
46	SFG_orb_R	17	64	0	Skills for dealing with feelings
47	IPG_L	−49	−33	44	Skills for dealing with feelings
48	PoCG_L	−32	31	71	Skill alternatives to aggression
49	PoCG_L	−47	−31	47	Skills for dealing with stress
50	PoCG_L	−39	−30	58	Planning skills
51	PrCG_L	−21	−20	70	Planning skills
52	Hipp_R	33	−16	−8	Planning skills
53	SMA_L	−7	−19	60	Kikuchi's Scale of Social Skills (KiSS-18)
54	IPG_R	46	−37	52	Public self-consciousness
55	SFG_L	−15	6	56	Private self-consciousness
56	TP_Mid_R	54	3	−29	Private self-consciousness
57	ACC_R	10	37	1	Private self-consciousness
58	MFG_R	31	37	48	Japanese Version of the Self-Concept Clarity (SCC) Scale
59	FG_R	44	−41	−15	Self-continuity function subscale
60	MTG_L	−60	−50	−8	Self-continuity function subscale
61	SPG_L	−12	−69	42	Directing-behavior function subscale
62	MOG_R	42	−71	35	Directing-behavior function subscale
63	SOG_L	−54	−54	37	Japanese version of the TALE (Thinking About Life Experiences) scale
64	FG_R	33	−15	−35	Decision Making (DM)
65	SPG_L	−29	−77	47	Decision Making (DM)
66	SMG_R	58	−47	31	Decision Making (DM)
67	MTG_R	66	−29	2	Impulsivity/Carelessness Style (ICS)
68	IFG_orb_R	28	22	−26	Impulsivity/Carelessness Style (ICS)
69	RectusG_L	−2	15	−19	Impulsivity/Carelessness Style (ICS)
70	IPG_L	−35	−76	44	Impulsivity/Carelessness Style (ICS)
71	IFG_orb_L	−22	16	−25	Impulsivity/Carelessness Style (ICS)
72	IFG_orb_L	−54	12	3	Avoidance Style (AS)
73	IFG_orb_L	−24	11	−27	Japanese version of the Social Problem-Solving Inventory-Revised (SPSI-R)
74	SOG_R	28	−67	25	Positive-Self (PS)
75	MFG_L	−44	29	20	Positive-Self (PS)
76	FG_L	−36	−73	−14	Positive-Self (PS)
77	Cerebellum_L	−44	−60	−43	Negative-Other (NO)
78	PCC_L	−6	−46	20	Positive-Other (PO)
79	ITG_R	61	−55	−21	Positive-Other (PO)
80	MFG_R	7	65	−7	Japanese version of the Brief Core Schema Scale (JBCSS)
81	Cerebellum_L	−21	−36	−29	Planfulness
82	Cerebellum_R	52	−59	−37	Planfulness
83	Cerebellum__R	30	−45	−37	Planfulness
84	SOG_R	26	−73	25	Planfulness
85	SOG_L	−27	−73	24	Planfulness
86	IPG_L	−40	−56	54	Planfulness
87	MFG_orb_R	44	54	−12	Readiness for change
88	MFG_R	38	39	22	Readiness for change
89	Cerebellum_R	51	−62	−43	Readiness for change
90	SFG_L	−20	18	52	Using resource
91	Cerebellum_L	−26	−41	−36	Japanese version of the Personal Growth Initiative Scale-II (PGIS-II)
92	Th_L	−2	−14	16	Subjective Happiness Scale (SHS)
93	Th_R	3	−17	14	Subjective Happiness Scale (SHS)
94	Cerebellum_R	48	−70	−34	The Satisfaction with Life Scale (SWLS)
95	Cerebellum_L	−44	−75	−31	The Satisfaction with Life Scale (SWLS)
96	Hipp_L	−32	−14	−10	The Satisfaction with Life Scale (SWLS)
97	IPG_L	−34	−39	41	State Anxiety (A-State)
98	IPG_R	35	−38	47	State Anxiety (A-State)
99	TP_Inf_L	−40	2	−36	Trait Anxiety (A-Trait)
100	Putamen_L	−27	−9	−5	Disorganization
101	MTG_L	−51	−51	−1	Disorganization
102	IFG_Med_R	3	56	−11	Disorganization
103	IFG_Tri_R	44	38	5	Disorganization
104	TP_Mid_L	−19	12	−42	Social skill
105	FG_R	31	−41	−10	Attention switching
106	IFG_Tri_L	−50	41	7	Attention switching
107	TP_Inf_R	43	6	−35	Attention switching
108	TP_Inf_R	52	6	−42	Attention switching
109	SFG_L	−19	69	5	Attention to detail
110	SFG_L	−9	−4	56	Attention to detail
111	Cerebellum_L	−12	−64	−29	Attention to detail
112	IFG_Tri_R	50	16	22	Communication
113	PrCG_R	49	3	26	Communication
114	Cerebellum_R	43	−54	−32	Imagination
115	SFG_Med	0	47	34	Imagination
116	PCC_L	−7	−44	17	Verbal IQ
117	PreCu_R	13	−59	44	Performance IQ
118	SFG_Med_R	11	48	−17	Full scale IQ
119	IFG_Med_R	5	40	−21	Full scale IQ
120	SFG_Med_L	−13	56	−5	Full scale IQ
121	PHG_L	−24	−24	−25	Full scale IQ
122	PCC_L	−7	−44	−16	Full scale IQ
123	PCC_L	−13	−43	9	Fluid intelligence
124	IFG_Tri_R	45	29	−1	Crystallized intelligence
125	SPG_L	−16	−57	53	Crystallized intelligence
126	PCC_L	−6	−38	23	Crystallized intelligence
127	TP_Sup_R	35	25	−30	Verbal comprehension
128	SFG_orb_L	−8	51	−21	Similarities
129	SFG_orb_R	14	49	−19	Similarities
130	Hipp_L	−22	−26	−11	Arithmetic
131	IFG_Tri_R	45	26	28	Arithmetic
132	PrCG_R	60	12	30	Arithmetic
133	SFG_R	22	35	54	Arithmetic
134	PrCG_L	−31	−4	64	Information
135	SMG_L	−59	−21	26	Information
136	PCC_L	−4	−42	18	Comprehension
137	SPG_L	−33	−44	57	Picture completion
138	Hipp_R	30	−39	3	Digit symbol
139	PoCG_R	55	−21	52	Digit symbol
140	SOG_R	27	−76	39	Digit symbol
141	PCC_L	−8	−43	9	Block design
142	IFG_Tri_L	−50	25	24	Matrix reasoning
143	IOG_R	29	−97	−9	Matrix reasoning
144	PoCG_R	49	−20	62	Picture arrangement
145	SMG_L	−53	−50	25	Emotional awareness
146	PoCG_R	62	−17	36	Self-efficacy
147	SMG_R	47	−40	24	Perseverance
148	Cerebellum_L	−16	−87	−29	Impulse control
149	PCC_M_L	−2	−17	32	Impulse control
150	PCC_M_R	3	−23	42	Impulse control
151	IFG_Tri_R	55	23	26	Patience
152	MFG_R	29	49	20	Patience
153	PrCG_L	−59	0	28	Patience
154	PrCG_L	−46	−5	28	Sharing positive emotion
155	ITG_R	56	−34	−16	Sharing negative emotion
156	MFG_R	38	32	17	Voluntary support
157	MTG_L	−48	−15	−9	Voluntary support
158	Th_R	10	−9	9	Decision making
159	MTG_L	−57	−46	−3	Decision making
160	ITG_L	−34	−3	−35	Optimism
161	MTG_L	−53	−8	−7	Group consideration
162	SPG_R	36	−71	52	Adaptability
163	MTG_L	−52	−16	−5	Situational awareness

### rs-fMRI

Functional networks were identified from the rs-fMRI time series. The rs-fMRI time series from the 163 ROIs were used to construct correlation matrices by correlation analysis (P3 of Figure 1A). After constructing of correlation matrices between ROIs (P4 of Figure 1A), a regression analysis was performed between the set of correlation matrices and the scores for the psychometric parameters to estimate statistically significant networks. Next, network-based statistics (NBS) were applied for network-based correction (P6 of Figure 1A). Networks were identified for a fixed significance level of *p* = 0.001 (corrected), which had different numbers of edges ranging from a few edges to more than 50. To obtain a similar number of edges for every identified network, two thresholds were used: one was at a significance level of *p* = 0.005 (corrected; see the Materials and Methods section for more details) and the other was the number of edges, namely, 15 per network (Termenon et al., 2016). Therefore, the number of edges in the identified networks were limited to as close to 15 as possible, except for those that had fewer than 15 edges even at the threshold of *p* = 0.005. For 116 of the 130 psychometric parameters, 128 significant functional networks were identified, of which 82 corresponded to social ability/skill parameters, 17 to IQ parameters and 29 to emotion quotient scale (EQS) parameters (P7 of Figure 1A). The functional networks corresponded to 116 psychometric parameters (74 for social ability/skill parameters, 15 for IQ parameters and 27 for EQS parameters), as 12 psychometric parameters corresponded to more than one functional network (8 for social ability/skill parameters, 2 for IQ parameters, and 2 for EQS parameters). The average number of edges of the 128 networks was 15.01 (mean) ± 4.72 (standard deviation). The set of all functional networks is shown in Figure S1 and Table S1. Each network identified through these processes can be considered to be involved in a brain function responsible for the corresponding psychometric parameter. Ultimately, we identified 128 functional networks representing 116 psychometric parameters, from which we can evaluate a wide variety of human characteristics that the 116 psychometric parameters reflect.

**Figure 3:**
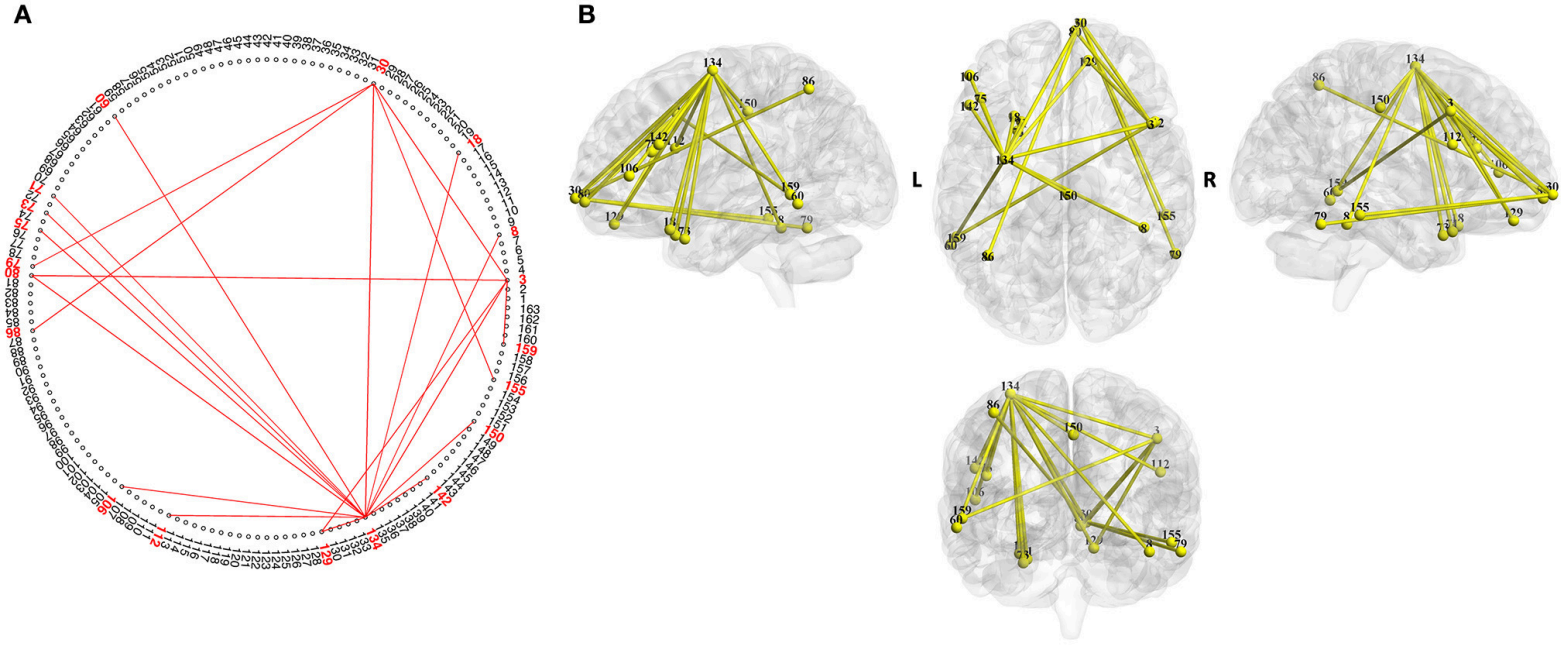
Brain map of the functional network for the psychometric parameter “verbal intelligence quotient (VIQ).” The width represents the strength of the correlation. The network was identified by NBS analysis (P5 of Figure 1A). This network reflects brain function related to “VIQ” and can be used to estimate VIQ. Similar assessments are possible for the other functional networks, and thus, comprehensive human characteristics can be evaluated. **(A)** Two-dimensional view of the network. **(B)** Three-dimensional view of the network.

As an example network, we show the network corresponding to the psychometric parameter “verbal intelligence quotient (VIQ)” (Figure 3) (Barona et al., 1984), as IQs are important in understanding human behavior and reflect diverse brain neurological differences (Deary et al., 2010). Among IQs, the VIQ reflects language ability, which is tightly associated with social function and is a basis of diverse human activities.

The ROIs constituting the functional network are shown in Table 4. The ROIs included in the brain areas of Brodmann area (BA) 47 and BA 22 are known to be associated with language-related function (Shaywitz et al., 1998).

**Table 4:** List of the ROIs included in the functional network corresponding to “Verbal IQ.”

**#**	**Region**	**MNI coordinates**			**Brodmann area**	**Psychometric parameters**
		***x***	***y***	***z***		
7	PoCG_R	44	−16	56	BA 4	Social competence with friends of the same sex
38	FG_R	39	−40	−16	BA 37	BAS/fun seeking
50	PoCG_L	−39	−30	58	BA 40	Planning skills
52	Hipp_R	33	−16	−8	BA 28	Planning skills
68	IFG_orb_R	28	22	−26	BA 47	Impulsivity/Carelessness Style (ICS)
95	Cerebellum_L	−44	−75	−31	Pyramis_L	The Satisfaction with Life Scale (SWLS)
102	IFG_Med_R	3	56	−11	BA 10	Disorganization
107	TP_Inf_R	43	6	−35	BA 38	Attention switching
113	PrCG_R	49	3	26	BA 6	Communication
126	PCC_L	−6	−38	23	BA 23	Crystallized intelligence
132	PrCG_R	60	12	30	BA 9	Arithmetic
139	PoCG_R	55	−21	52	BA 2	Digit symbol
147	SMG_R	47	−40	24	BA 13	Perseverance
150	PCC_M_R	3	−23	42	BA 31	Impulse control
153	PrCG_L	−59	0	28	BA 6	Patience
154	PrCG_L	−46	−5	28	BA 6	Sharing positive emotion
155	ITG_R	56	−34	−16	BA 20	Sharing negative emotion
163	MTG_L	−52	−16	−5	BA 22	Situational awareness

### SVM

To verify that the functional networks identified from the rs-fMRI signals and psychometric parameters can significantly represent cognition/behavior, we derived a multiclass SVM classifier for each functional network. A binary classifier was considered to be sufficient for verification, but we attempted to derive multiclass SVM classifiers because our future aim is to estimate scores of psychometric parameters using only rs-fMRI data. To determine if this was possible, an eight-class SVM classifier was chosen, although a multiclass SVM with more classes would have been ideal for estimating the scores. A multiclass (eight-class) SVM classifier for each psychometric parameter could be derived by using the edges of each network as the input (P8 of Figure 1A). All the classifiers were revealed to have significant accuracy upon cross-validation (one-sample *t*-test, *p* = 0.05) above the chance level, although the accuracy is low, except for five classifiers corresponding to five psychometric parameters (P9). That is, significant classifiers were derived for 123 of the 128 functional networks (78 for social ability/skill parameters, 17 for IQ parameters and 28 for EQS parameters). These 123 SVM classifiers were related to 111 psychometric parameters (70 for social ability/skill parameters, 15 for IQ parameters, and 26 for EQS parameters), as 12 psychometric parameters corresponded to more than one classifier (Figure S2, Table S1).

### Testing the SVM classifiers in different populations

**Figure 4:**
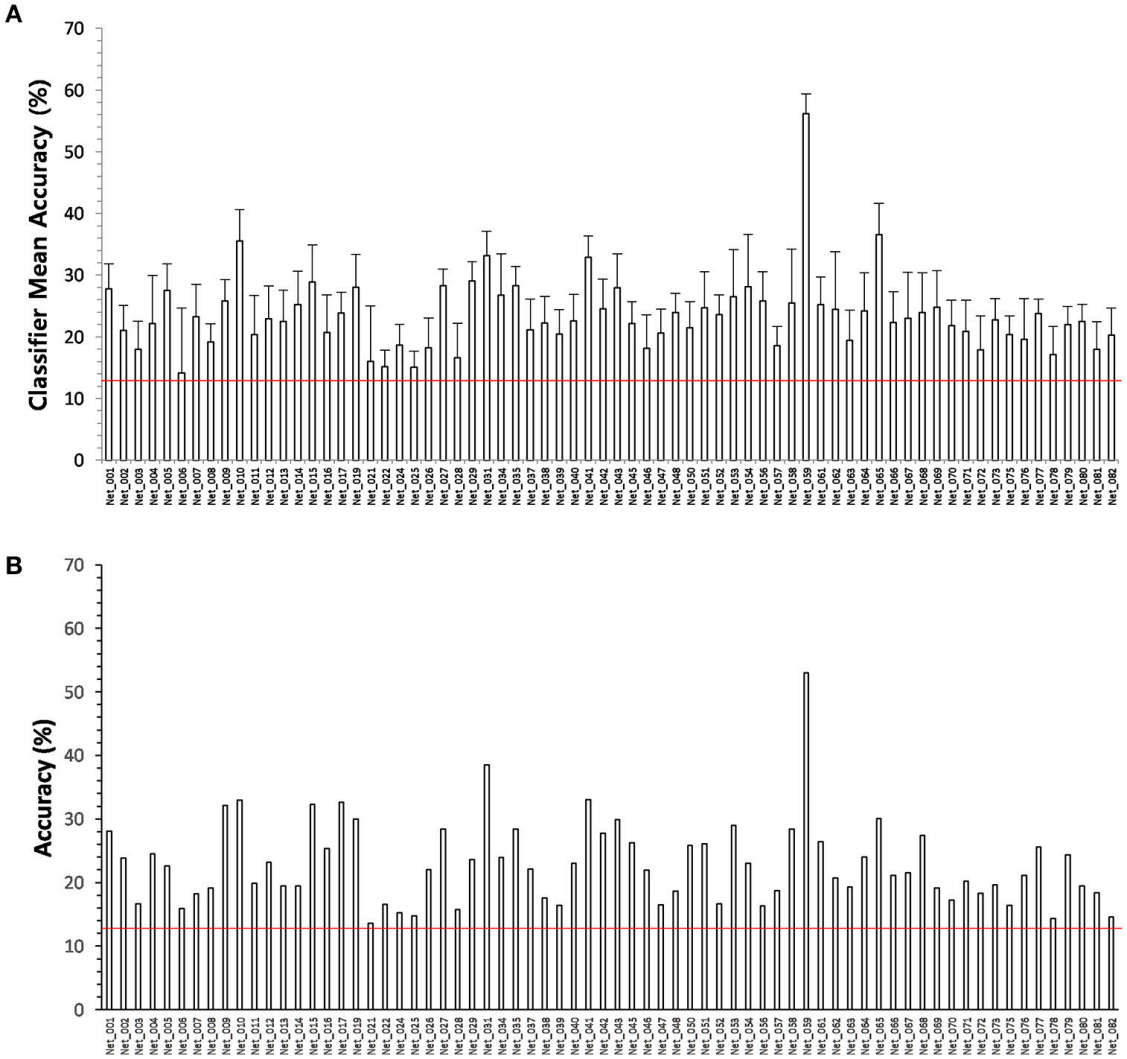
**(A)** Accuracies of 70 SVM classifiers corresponding to the psychometric parameters related to social ability/skill in 153 participants. The error bar denotes the standard deviation. All classifiers are significant at *p* = 0.05. **(B)** Accuracies of same 70 SVM classifiers in another 57 participants. The accuracy results of test are similar to those observed during the training. This shows that the derived classifiers work properly. The redlines on the figures indicate the chance level 12.5%.

To test the performances of the SVM classifiers, we estimated the scores of the psychometric parameters from level 1 to 8 (related to social ability/skill) from MRI data using the 70 SVM classifiers (only one classifier was chosen for each psychometric parameter that corresponded to multiple classifiers) that corresponded to the 70 psychometric parameters related to social ability/skill. We compared the estimated scores with psychologically measured scores of the 70 psychometric parameters for 57 participants in the supplementary experiment. All 70 SVM classifiers exhibited an accuracy above chance level with low sensitivity of 14.1 ± 3.7% but high specificity of 87.8 ± 0.8% (Table 5), and the accuracy results found for this supplementary experiment were similar to those observed in the primary experiment (Figure 4).

**Table 5:** Sensitivity and specificity of classifiers.

	**Sensitivity**	**Specificity**
Net_001	0.30	0.71
Net_002	0.25	0.62
Net_003	0.33	0.67
Net_004	0.27	0.62
Net_005	0.33	0.67
Net_006	0.32	0.64
Net_007	0.25	0.61
Net_008	0.29	0.65
Net_009	0.42	0.73
Net_010	0.33	0.67
Net_011	0.33	0.67
Net_012	0.33	0.68
Net_013	0.38	0.67
Net_014	0.33	0.67
Net_015	0.33	0.69
Net_016	0.30	0.64
Net_017	0.36	0.69
Net_019	0.29	0.66
Net_021	0.33	0.67
Net_022	0.32	0.66
Net_024	0.28	0.63
Net_025	0.24	0.63
Net_026	0.29	0.65
Net_027	0.29	0.67
Net_028	0.33	0.67
Net_029	0.38	0.72
Net_031	0.33	0.67
Net_034	0.33	0.67
Net_035	0.32	0.66
Net_037	0.28	0.62
Net_038	0.30	0.65
Net_039	0.33	0.67
Net_040	0.34	0.67
Net_041	0.30	0.65
Net_042	0.34	0.67
Net_043	0.34	0.68
Net_045	0.32	0.66
Net_046	0.39	0.71
Net_047	0.35	0.66
Net_048	0.33	0.67
Net_050	0.39	0.69
Net_051	0.32	0.67
Net_052	0.32	0.66
Net_053	0.28	0.62
Net_054	0.28	0.64
Net_056	0.33	0.67
Net_057	0.33	0.62
Net_058	0.35	0.68
Net_059	0.30	0.67
Net_061	0.31	0.65
Net_062	0.39	0.72
Net_063	0.39	0.70
Net_064	0.31	0.69
Net_065	0.45	0.66
Net_066	0.33	0.67
Net_067	0.31	0.65
Net_068	0.33	0.67
Net_069	0.28	0.64
Net_070	0.31	0.64
Net_071	0.33	0.66
Net_072	0.35	0.69
Net_073	0.40	0.69
Net_075	0.33	0.67
Net_076	0.31	0.64
Net_077	0.33	0.70
Net_078	0.33	0.67
Net_079	0.28	0.66
Net_080	0.32	0.66
Net_081	0.44	0.75
Net_082	0.33	0.65

## Discussion

The primary aims of this study were to prove that functional networks identified by rs-fMRI signals and psychometric parameters represent brain functions to which corresponding cognition/behavior are related and to determine whether the functional networks can be interpreted in a similar way to functional areas identified by tb-fMRI signals upon task stimulation. Another aim was to identify a set of functional networks for comprehensively evaluating human characteristics.

We found functional networks corresponding to 111 out of 130 psychometric parameters and derived a multiclass SVM classifier for each psychological parameter. These findings demonstrate that each rs-fMRI functional network can represent a corresponding cognition/behavior and that the set of functional networks reported here, which correspond to 111 psychometric parameters, can be used to comprehensively evaluate human characteristics.

Previous studies have attempted to identify functional networks from rs-fMRI signals. Some have identified functional networks by correlation between rs-fMRI signals and known ROIs (Wang et al., 2009; Tian et al., 2011), and others have derived functional networks by correlation between a known seed area or image voxel of the brain and other brain areas/voxels (Greicius et al., 2003; Fox et al., 2009) or by independent component analysis (ICA) in which a group of brain areas sharing a common component of an independent signal are identified to constitute a functional network (Damoiseaux et al., 2006; De Luca et al., 2006). However, the functional roles of the networks were interpreted based on various cognition/behavior related to the tasks in tb-fMRI (van den Heuvel and Hulshoff Pol, 2010), as a combination of functional areas previously identified by tb-fMRI and the tasks performed during tb-fMRI were used in the identification procedure of the functional networks. Therefore, it remains unclear whether functional networks can be identified by psychological indices (psychometric parameters consisting of self-reported questionnaire scores) and whether rs-fMRI signals can represent the corresponding cognition/behavior. By identifying brain networks related to psychometric parameters and the deriving multiclass SVM classifiers corresponding to those psychological parameters, our results demonstrate that rs-fMRI functional networks can represent cognition/behavior. In tb-fMRI, SVM classifiers have been used to prove functional specificity of a brain area by testing whether the brain area can discriminate the related stimulus exemplar from other stimuli (MacEvoy and Epstein, 2009). Similarly, each of our identified classifiers could significantly discriminate the scores of the corresponding psychological parameter, showing that each functional network represented the corresponding cognition/behavior. The results indicate that the identified functional networks can be used in a similar way to the functional areas identified by tb-fMRI for brain imaging. Recent studies on rs-fMRI support our results that the functional brain networks identified by rs-fMRI signals contain intrinsic information of the brain system and have shown that similar brain maps obtained by tb-fMRI can also be acquired by rs-fMRI (Finn et al., 2015; Tavor et al., 2016).

The identification of 111 functional networks and the derivation of SVM classifiers for each of the 111 functional networks suggest that the variance of rs-fMRI signals between subjects reflects differences in cognition/behavior, which also indicates that variations in neural systems develop differently in each individual. Therefore, it is inferred that brain plasticity with different genetic and environmental conditions (Bouchard, 2004) varies among participants and that such variation in plasticity could appear in the functional network representation.

Several studies of brain plasticity at the systems level have been performed. Long-term plasticity has been shown in primary sensory areas, such as in visual areas of blind subjects and auditory areas of deaf subjects (Karni and Sagi, 1991; Gaser and Schlaug, 2003), which could be considered as modifications to brain “hardware.” Mid-term plasticity was also shown in high-level areas like the hippocampus among people who have extensively trained for many years, such as in taxi drivers (Maguire et al., 2000). In addition, short-term plasticity was shown in high-level areas such as the hippocampus in those undergoing intense cognitive function training, such as in those studying for a difficult medical exam for a few months or during physical juggling training for a few weeks (Draganski et al., 2006). Such plastic changes have been observed by rs-fMRI and VBM. Considering the brain plasticity observed by rs-fMRI or VBM, we can infer that the 163 functional areas and 111 functional networks identified by the same modalities used in the previous studies also reflect brain plasticity.

We performed the leave-one-out cross validation in this study because we thought it gives less biased predictions. Recently a study has suggested conservative evaluation for reliability of cross-validation methods in applying machine learning algorithms for small sample sizes (Varoquaux, 2017). In this study, we completely separated data sets for the training and the test and could acquire similar results for the training and the test. In addition, the average accuracy 23.6% of the test data is still above a corrected chance level (about 17% for 150 samples), corrected by the number of samples, which was proposed by a previous study (Combrisson and Jerbi, 2015). These support that our classifiers are reliable.

In terms of the general use of classifiers, accuracy is an important factor. The accuracy of the SVM classifiers is low, although it is above the chance level. One reason is that the number of classes - 8 - could be too high. For 3-class SVM classifiers we could get about 53% accuracy; similarly, we can expect about 80% accuracy for a binary classifier. In this study, we used the same number of classes for all psychometric parameters when designing SVM classifiers, although there was an optimal number of clusters. We used the same algorithm and kernel for all psychometric parameters. However, other classification algorithms or kernels may be better for some psychometric parameters depending on their features and data structures. In future studies, it will be necessary to optimize the classification algorithm to each parameter and to optimize the number of classes to suit the evaluation of human characteristics, especially transient changes in characteristics by brain plasticity resulting from education, training, or diseases. However, even with the 8-class SVM classifier we derived, we can significantly evaluate human characteristics. For example, in a supplementary experiment (not published), we obtained rs-fMRI data from one participant 8 times across 2 weeks. We found that 66 out of 70 SVM classifiers gave the same score (level) more than 4 times, which means that repeated measurements enable our 8-class SVM classifiers to be applied to estimate a wide variety of human characteristics with high specificity (more than 87%; Table 5).

For training, the SVM classifier that we used here only has information regarding the edges of the functional networks with which the correlations of the psychometric scores were comparatively low, 0.51 ± 0.07 (mean ± SD), as shown in Figure S2, which might be a limitation of the performance of the SVM classifiers. But other factors related to the topology of a functional network as a graph, such as centrality and mean path, can be used to improve the accuracy. Additional data from other modalities, such as fractional anisotropy or mean diffusivity from diffusion-weighted imaging, may also be useful for further improving the accuracy. In the future, we plan to use these types of data to further improve the accuracy for generalizing our classifiers.

The tb-fMRI signal is known to be induced by stimulation through neuro-vascular coupling, and the site at which an fMRI signal is measured is known to contain neurons that are processing the information related to a given stimulus. Therefore, tb-fMRI is known to be a direct method to identify functional areas. However, resting-state fMRI is considered to be indirect because additional supporting information is needed to characterize the connectivity, such as psychological parameters or behavioral information, and it remains unclear whether neurons in brain areas of a rs-fMRI functional network are directly related to the processing of the information required for the expected cognitive or behavioral brain function. Therefore, even in the case in which the data acquired by the two modalities can yield the same results in evaluating cognition/behavior, the functional mechanism represented by brain areas or networks may be different. This may explain why ROIs for a psychometric parameter were not identified but a functional network was identified. This type of problem related to intrinsic functional characteristics should be further examined to elucidate the mechanism of brain function.

To understand the details of the functional role of the functional network of each psychological parameter, tb-fMRI may be needed. However, in this study, the aim was to prove that functional networks can be identified from rs-fMRI signals and psychometric parameters and to identify biomarkers of cognition/behavior to evaluate a wide variety of human characteristics that can be used to describe individuals. Therefore, although the details of identified functional networks should be investigated further, we believe that our aim of identifying functional networks/classifiers that characterize most of the intended psychometric parameters has been achieved.

Regarding the size of the subject population for significantly obtaining brain information from rs-fMRI and psychological parameters, the population size of 153 subjects for this study is considered to be appropriate because a previous study suggested that more than 100 subjects can provide reliable variation for estimating brain plasticity (Termenon et al., 2016).

To the best of our knowledge, this study derived the largest number of functional networks/classifiers (or identified functional brain networks) reflecting cognition/behavior among fMRI studies performed to date. Although there are several large databases around the world that are represented by the “human connectome project” (www.humanconnectome.org), none of the databases include as many psychometric parameters as included in this study. This is the first study of its kind in the field of brain imaging that reveals the possibility to describe an individual based on a comprehensive set of diverse human characteristics. However, other psychometric parameters may need to be added to obtain more classifiers so that an individual can be described as completely as possible. In the present study, 19 psychological parameters were not significantly associated with a functional network, for which we may need to devise new tasks and use tb-fMRI to elucidate the corresponding functional networks. In addition, it may be possible that abilities or emotions related to the psychometric parameters are not reflected in resting state fMRI signals, which may be an interesting topic to investigate in a future study.

Taken together, our results demonstrate that; (i) rs-fMRI signals include intrinsic information of brain function related to cognition/behavior, (ii) functional networks identified by psychometric parameters can represent corresponding cognition/behavior, and (iii) the set of functional networks/classifiers identified here can be used to comprehensively evaluate human characteristics.

## Conclusion

We identified a set of 128 functional networks of cognition/behavior by rs-fMRI that span a variety of human characteristics and psychometric parameters, and we derived 123 multiple-class SVM classifiers corresponding to 111 psychometric parameters. This demonstrates that we can identify functional areas or networks of the brain not only by tb-fMRI but also by rs-fMRI. It also demonstrates that we can evaluate cognition/behavior and develop biomarkers for a wide variety of human characteristics using the 111 dimensions of the data obtained from a single rs-fMRI scan. The data and classifiers may also be applied to longitudinal studies or studies evaluating educational, training, or career development programs.

## Author contributions

Y-WS, YK, and SO designed this study. The psychological data for this study were acquired by YK, CA, and YO. The anatomical and functional magnetic resonance images (MRI) data were acquired by Y-WS and DK. The VBM analysis of anatomical data was conducted by Y-WS and DK. Brain network analysis and SVM analysis of functional data was conducted by U-SC. Y-WS wrote the first draft of the manuscript with SO, YK, and U-SC contributed to revise this manuscript. All authors reviewed this manuscript.

### Conflict of interest statement

The authors declare that the research was conducted in the absence of any commercial or financial relationships that could be construed as a potential conflict of interest.

## Supplementary material

Figure S1 Set of all functional networks.

Figure S2 Correlation between each functional network and its psychometric score for 70 functional networks with 153 subject data by a linear multiple regression based on the least square analysis. The average correlation value *R* is 0.51 (*S.D* 0.07) and *R*^2^ is 0.27 (*S.D* 0.07), *p* < 0.0001.

Table S1 Description of all functional networks.

Supplementary Datasheet 1 Supplementary references.
