# Supplementary material for: A Set of Functional Brain Networks for the Comprehensive Evaluation of Human Characteristics
Source: Front Neurosci. 2018 Mar 14;12:149. doi: 10.3389/fnins.2018.00149 (PMC5861187; doi:10.3389/fnins.2018.00149)

Cognitive competence

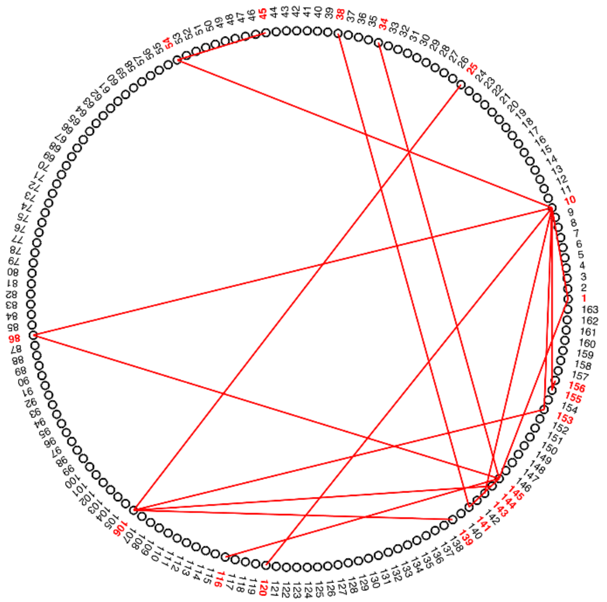

Extracurricular competence
including physical competence

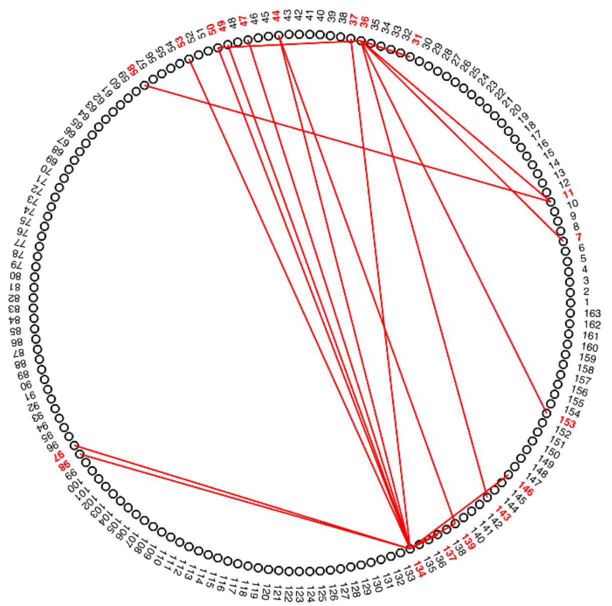

General self-worth

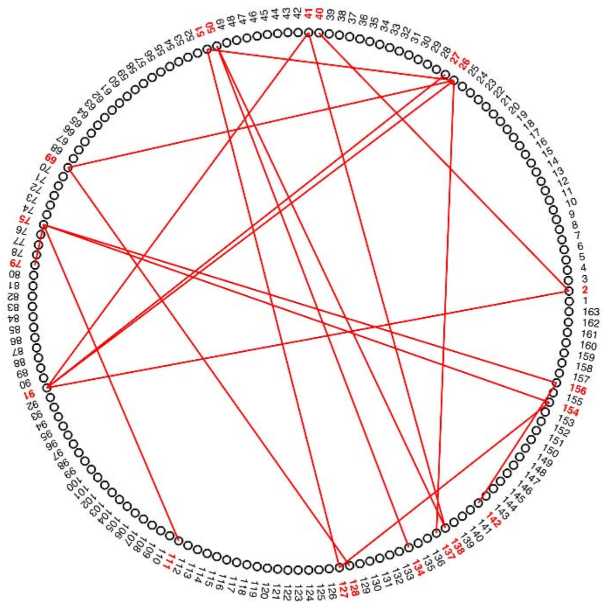

Perceived Competence Scale for Adolescence1

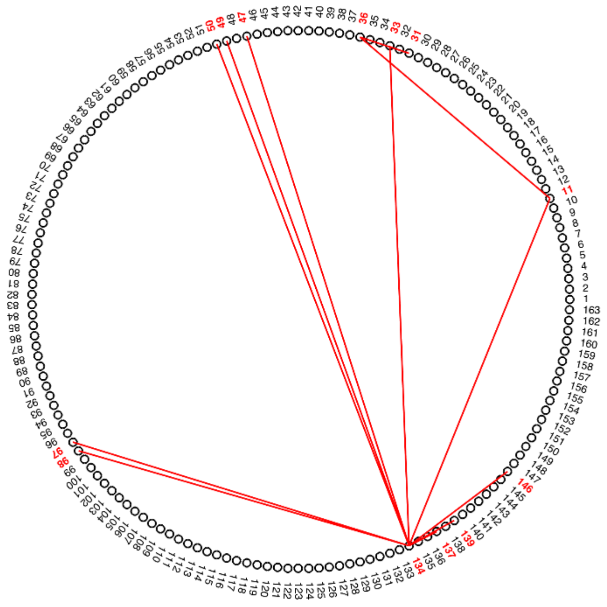

Inhibitory Control

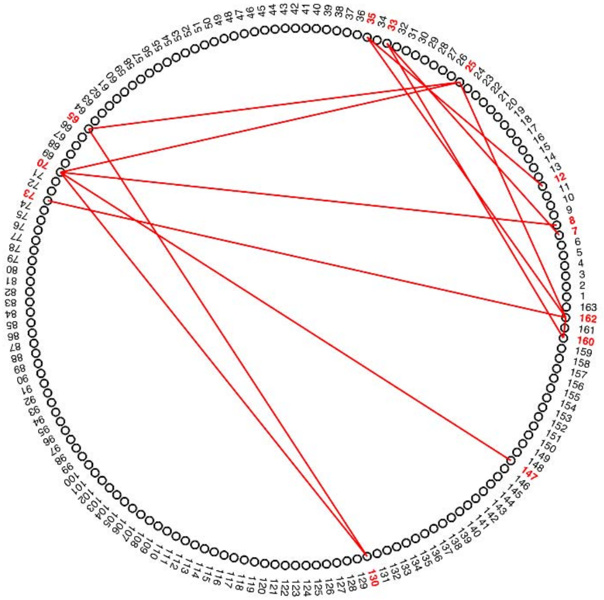

Attentional Control

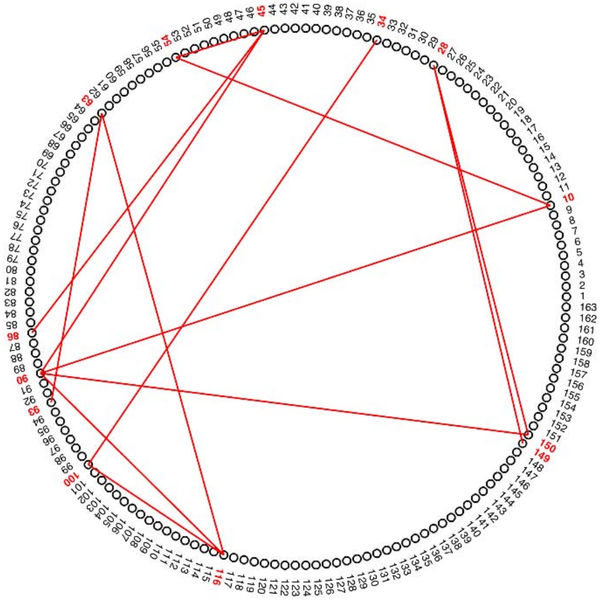

Perceived Competence Scale for Adolescence2

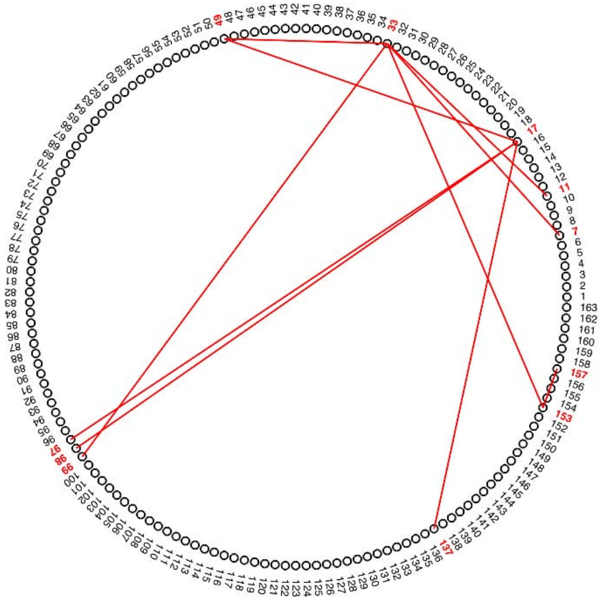

Trust vs. Mistrust

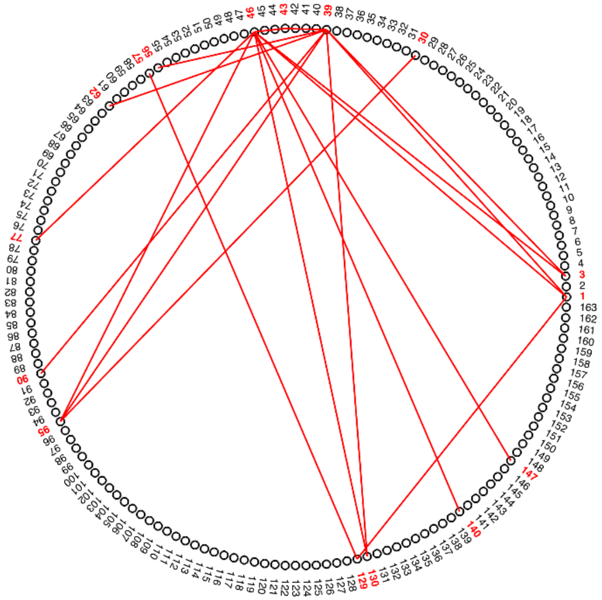

Autonomy vs. Shame/Doubt

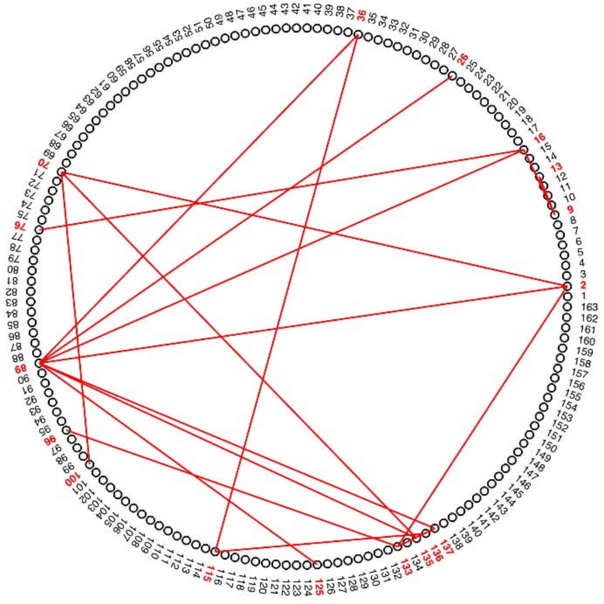

Initiative vs. Guilt

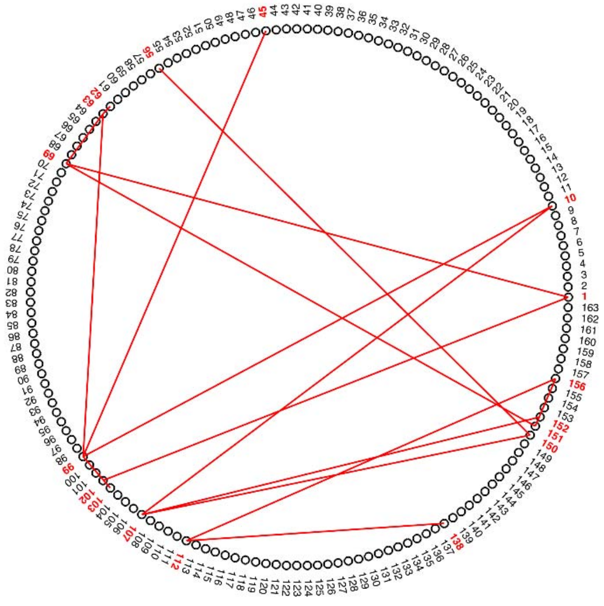

Industry vs. Inferiority

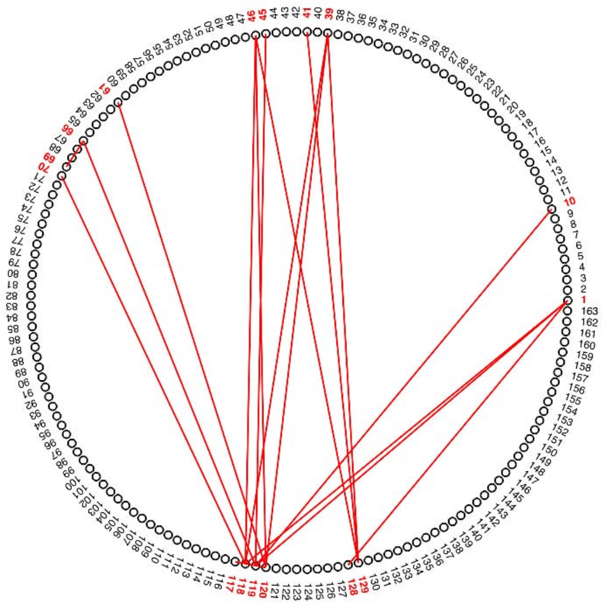

Identity vs. Role Confusion

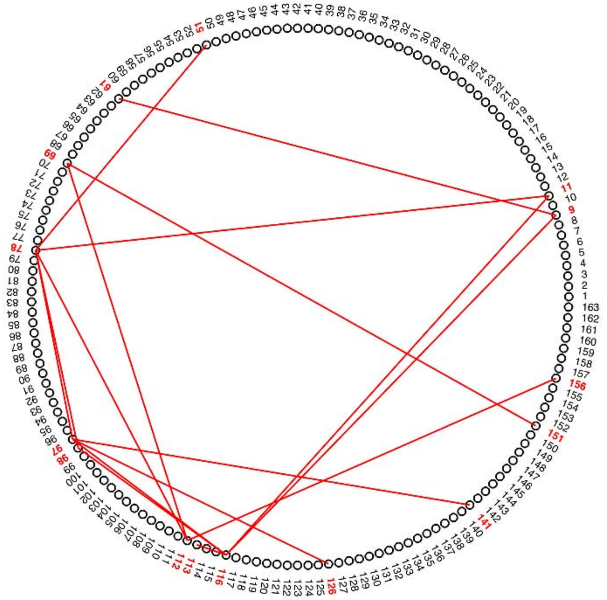

Intimacy vs. Isolation

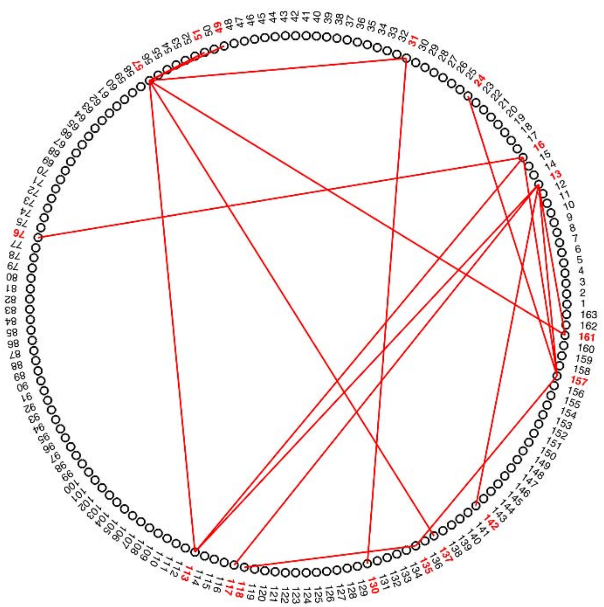

Japanese version of Rasmussen's Ego Identity Scale (REIS)

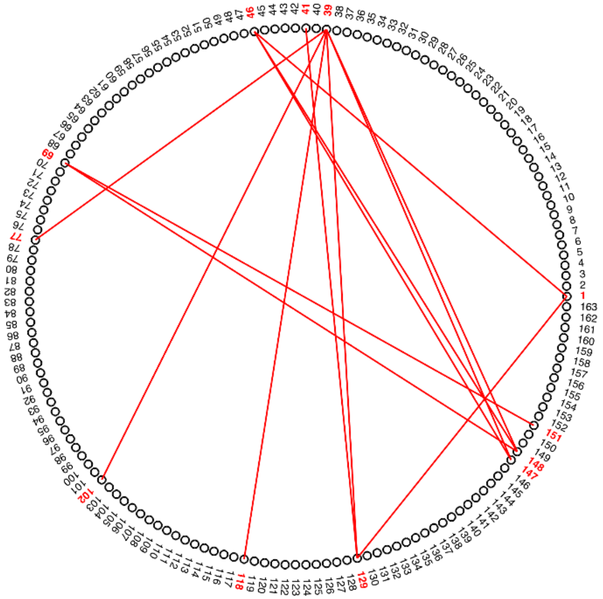

Behavioral Inhibition System: BIS

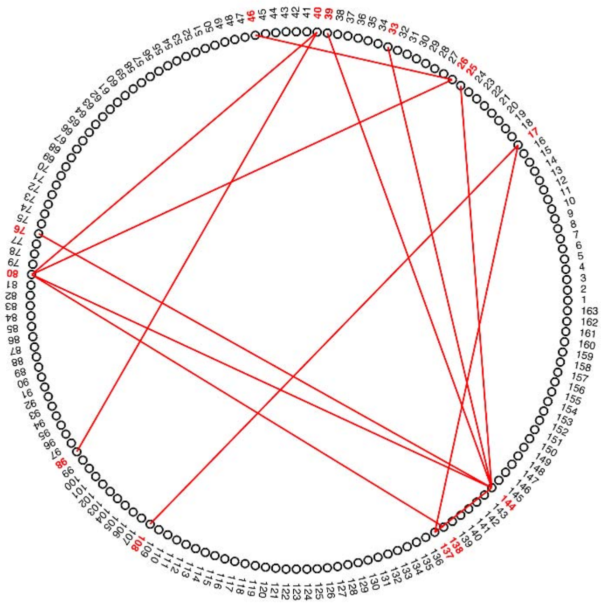

BAS / Driver

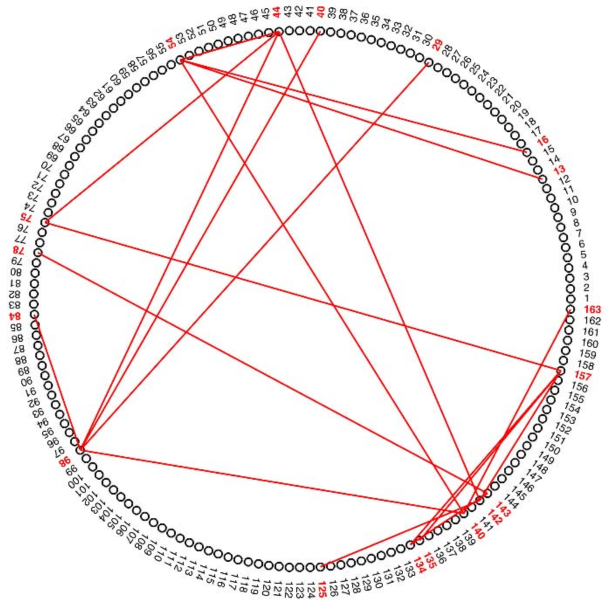

BAS / Reward

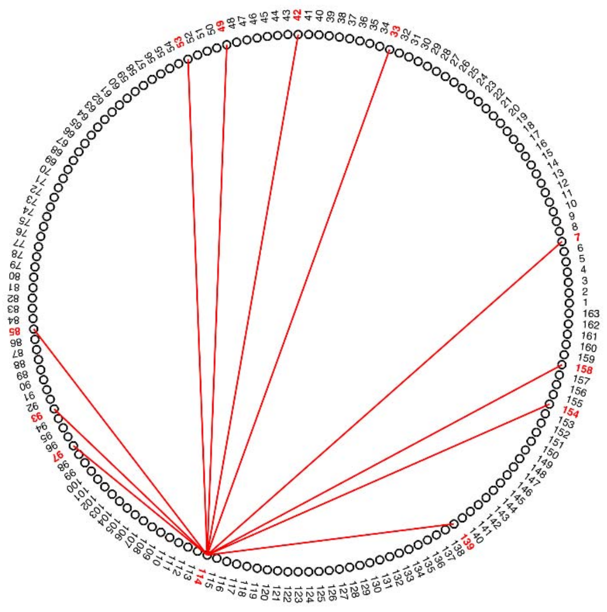

Behavioral Inhibitory System(BIS)
/ Behavioral Activate System (BAS) Scale

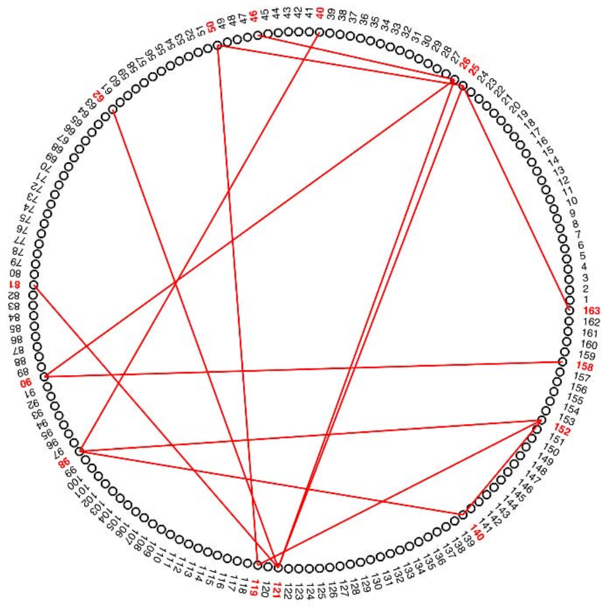

BAS / Fun Seeking

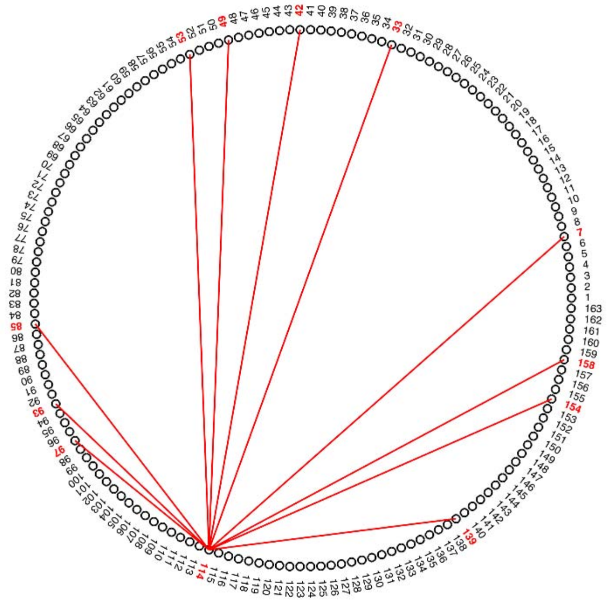

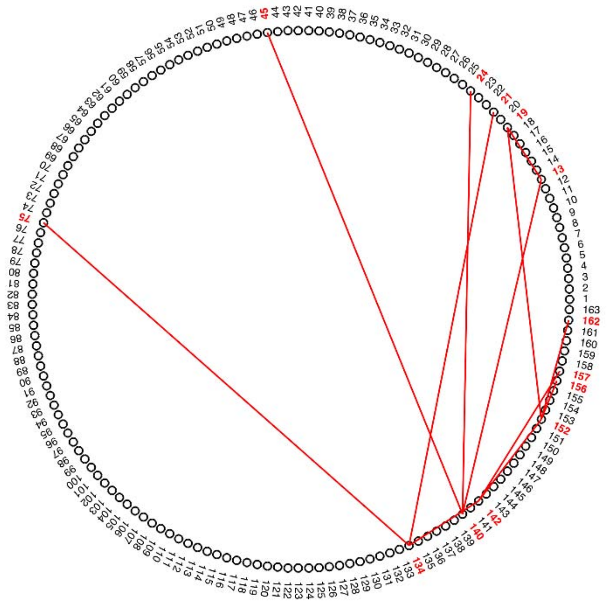

Japanese version of Rasmussen's Ego Identity Scale (REIS)2

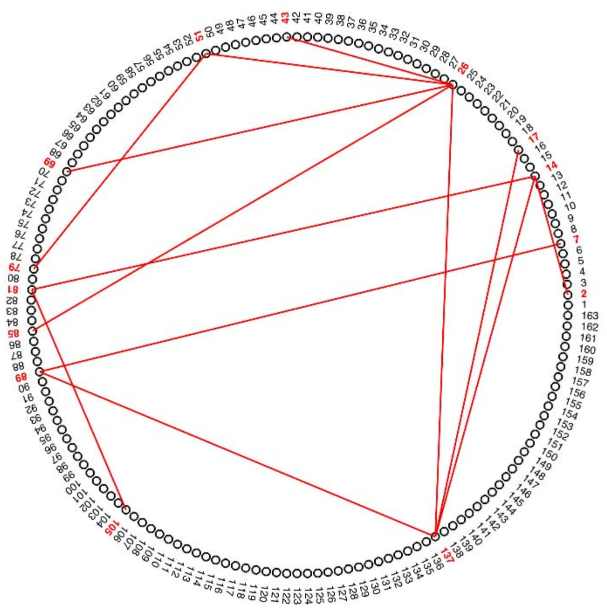

Emotional disturbance

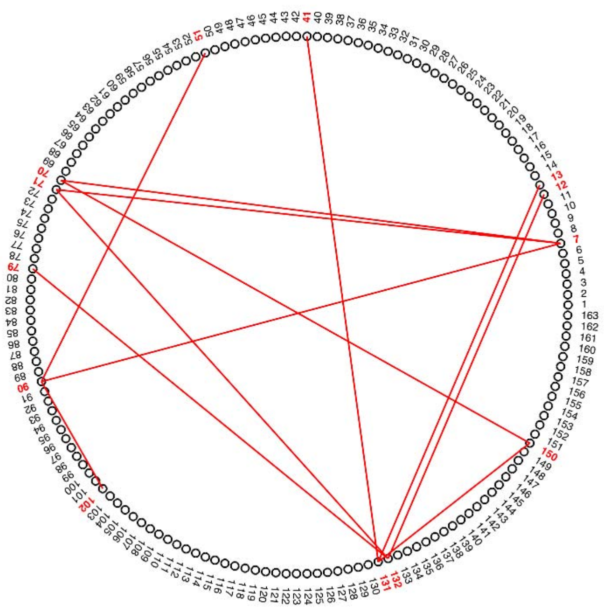

Anxiety regarding others' evaluation of oneself and perceived maladjustment to interpersonal situations

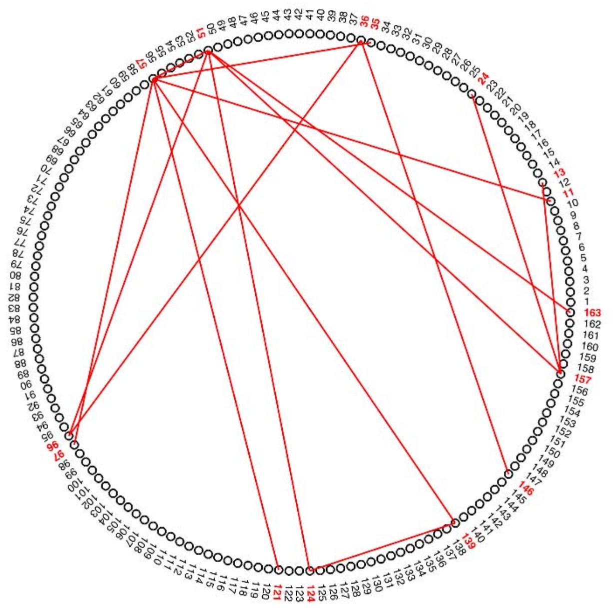

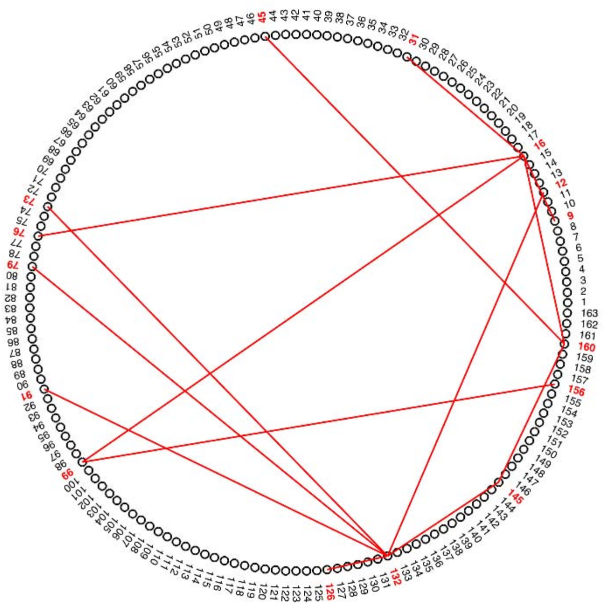

Difficulty in expressing opinions

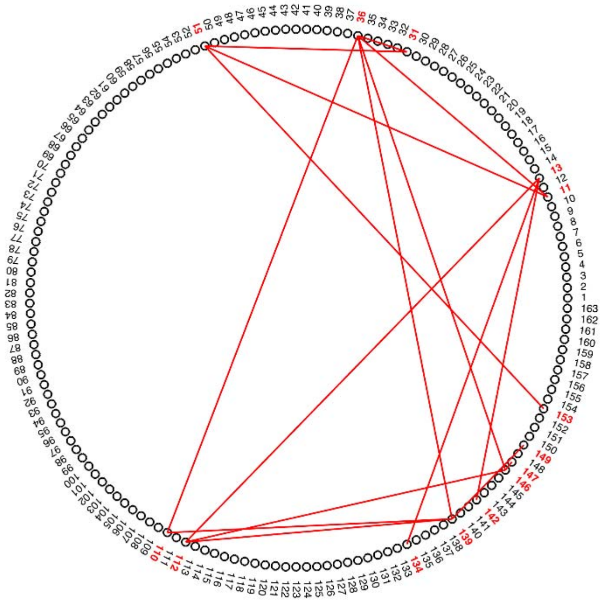

Jones and Russell's Social Reticence Scale
for college students

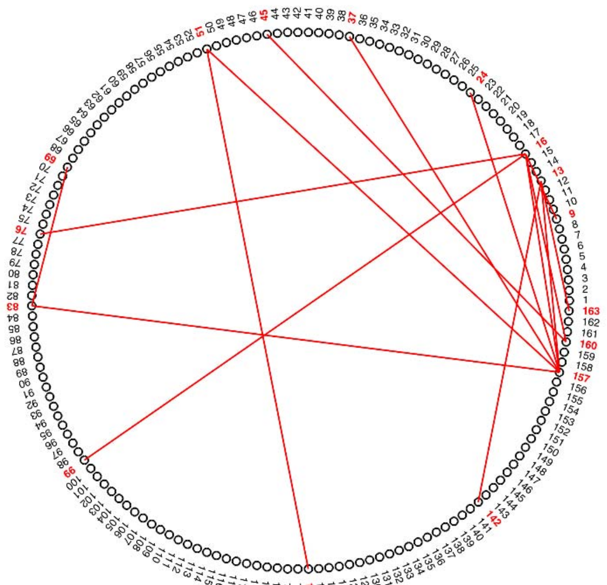

beginning social skills

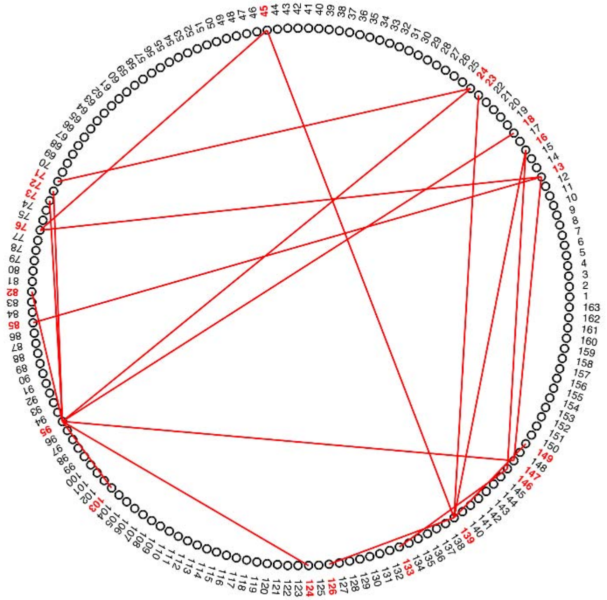

Advanced social skills

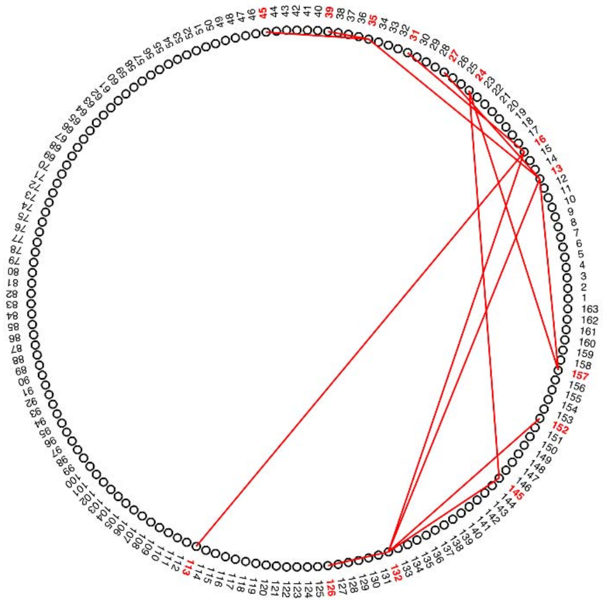

Skills for dealing with feelings

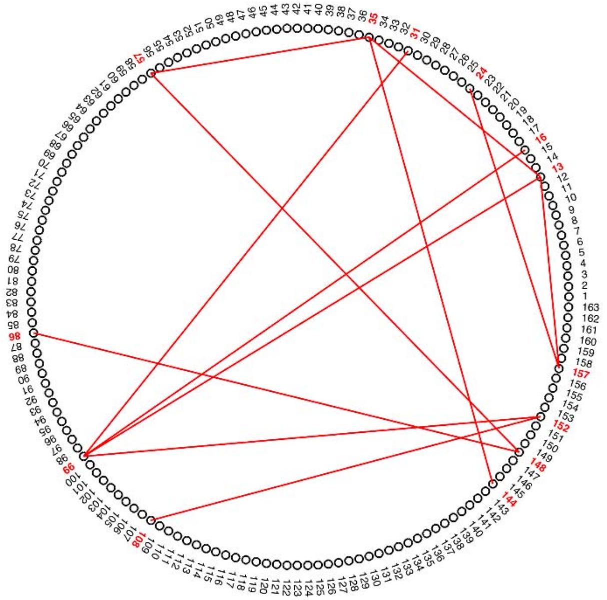

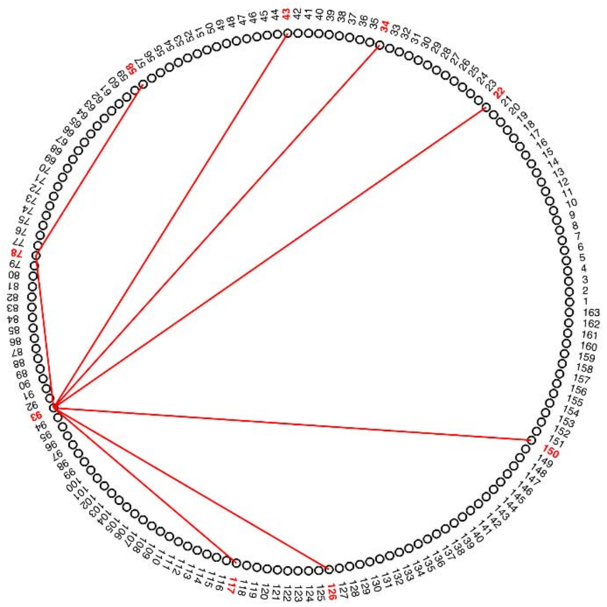

Skill alternatives to aggression

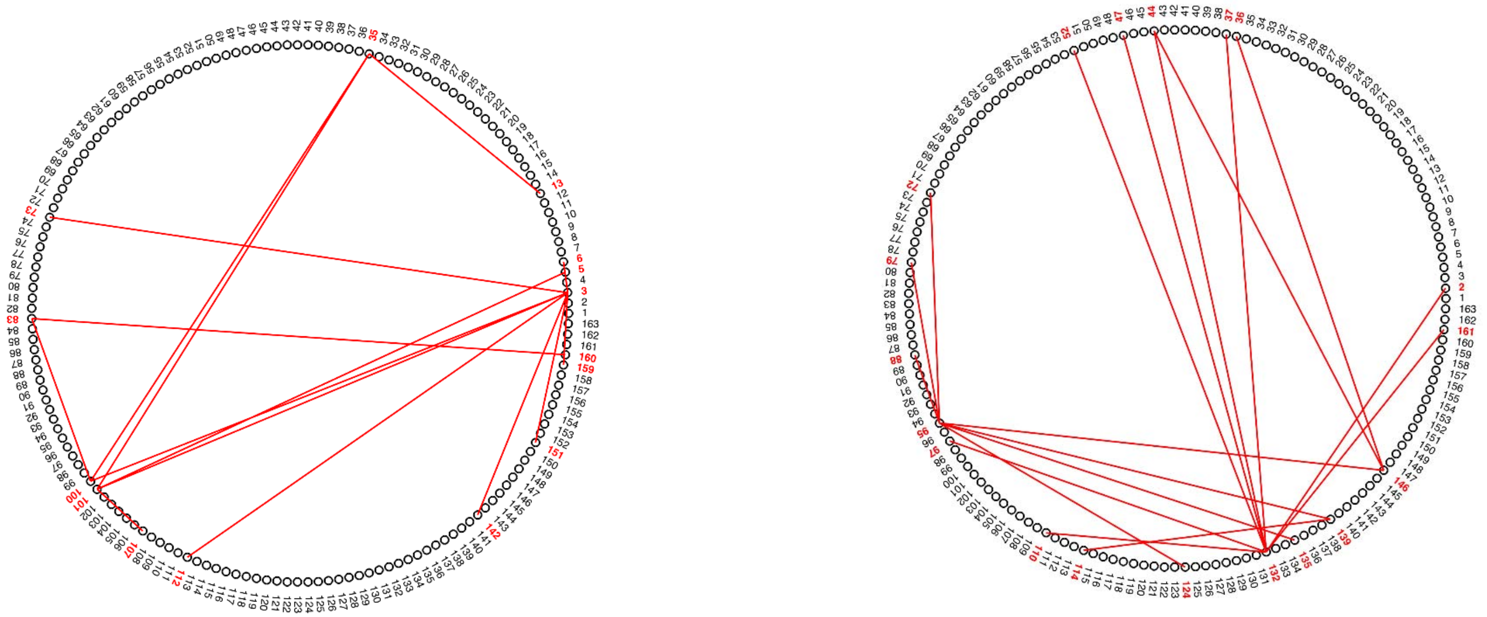

Skills for dealing with stress

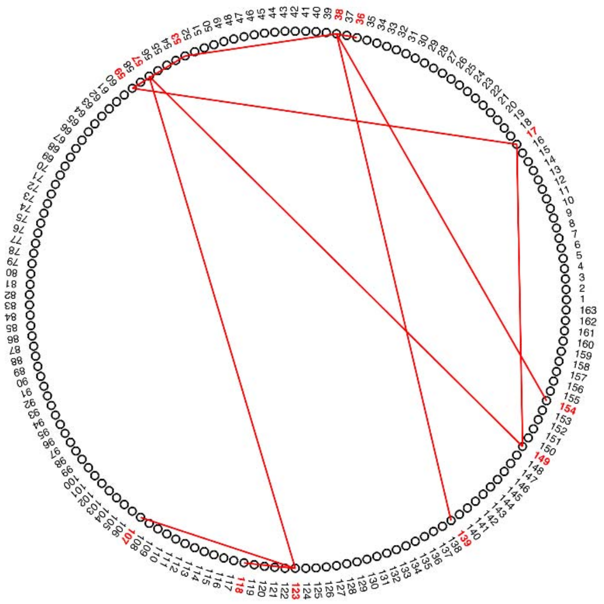

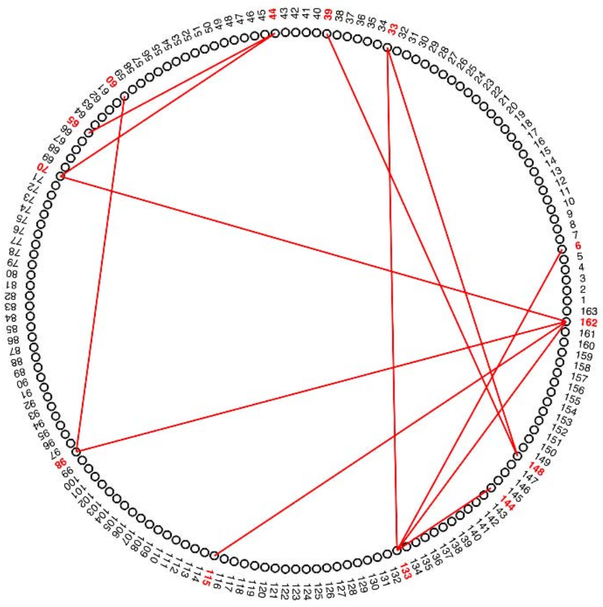

Planning skills

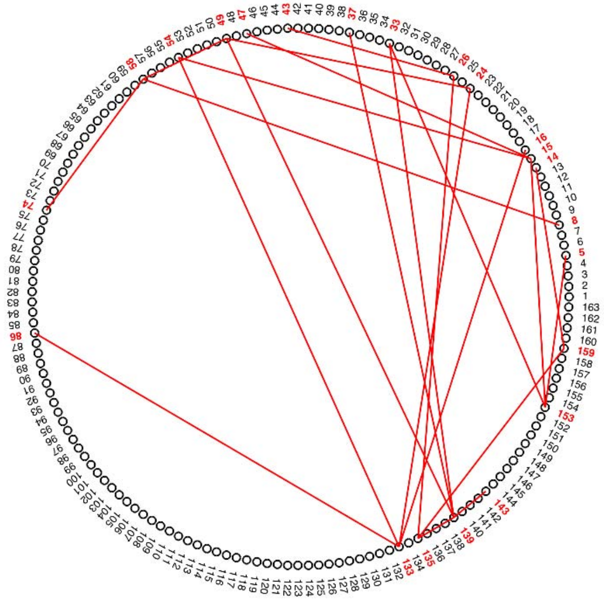

Kikuchi's Scale of Social Skills (KiSS-18)

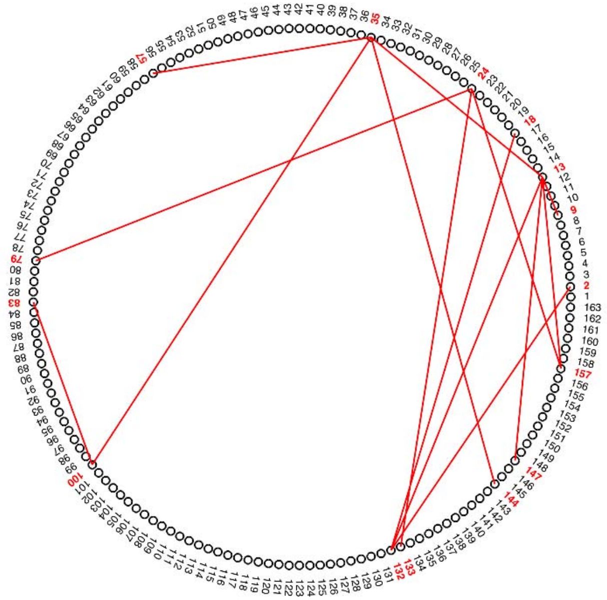

Public self-consciousness

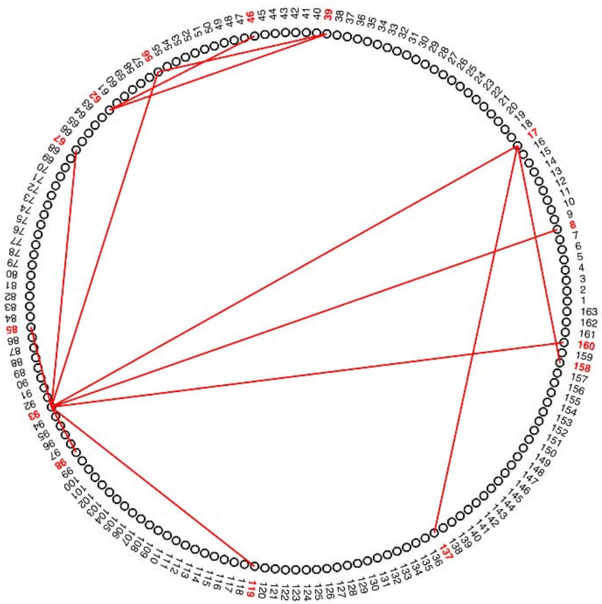

Private self-consciousness

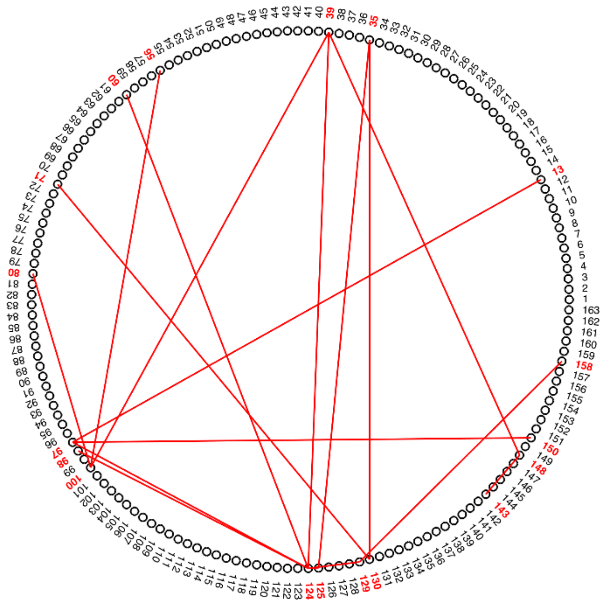

Self-consciousness scale for Japanese

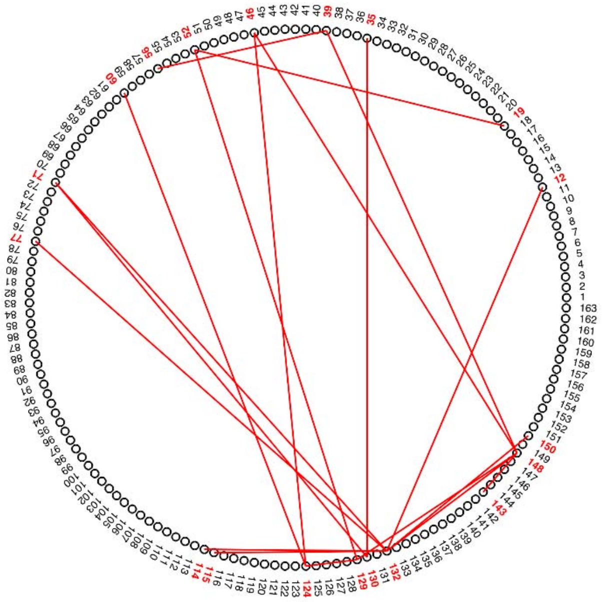

The Self-Concept Clarity (SCC) Scale

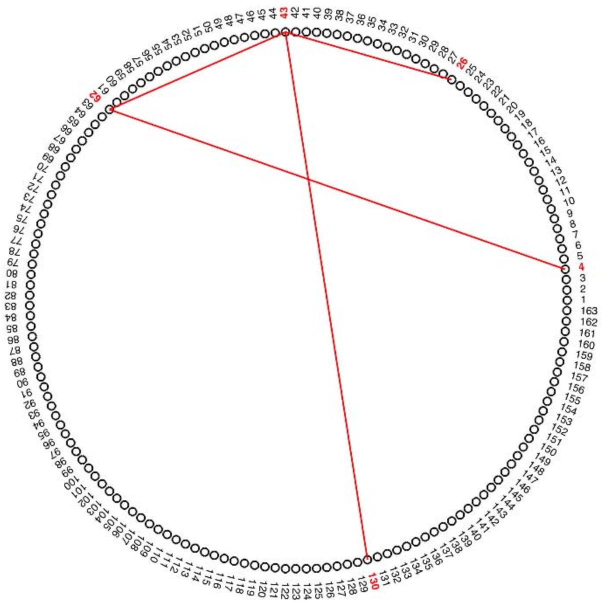

Directing-Behavior Function Subscale

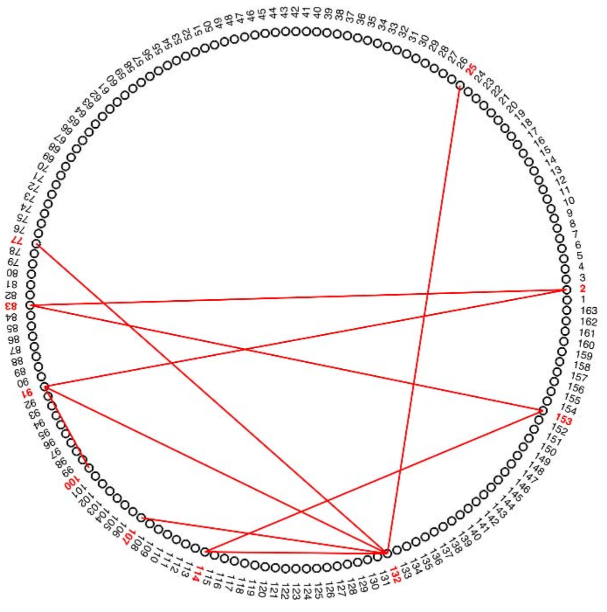

Social-Bonding Function Subscale

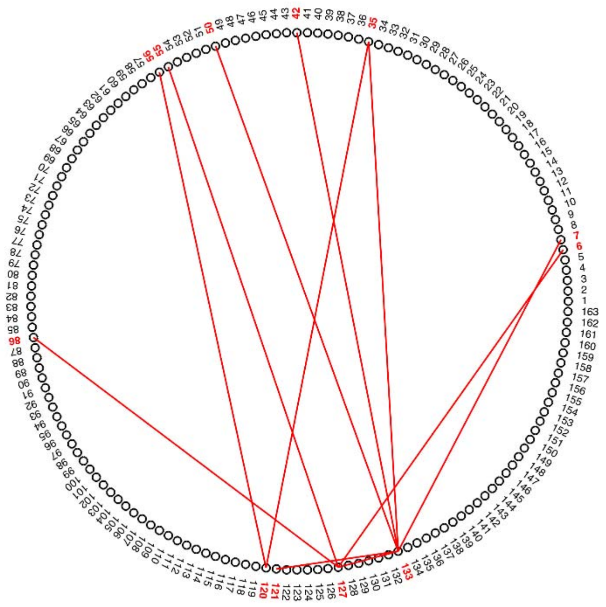

TALE (The Thinking About Life Experiences) Scale

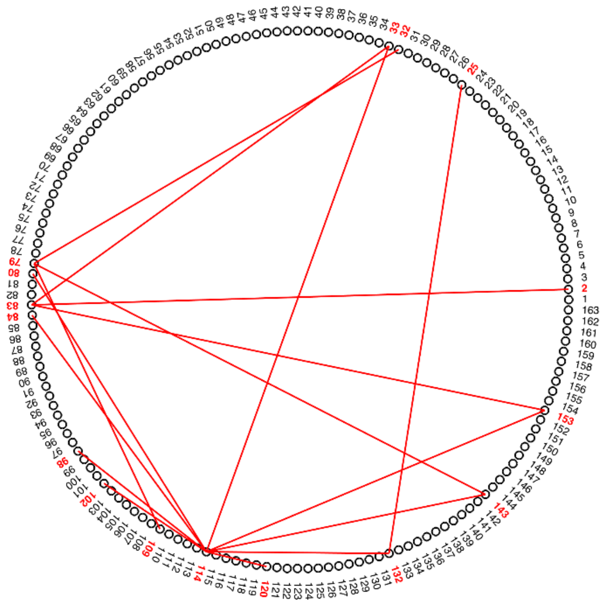

The Ego-Resiliency Scale (ER89)

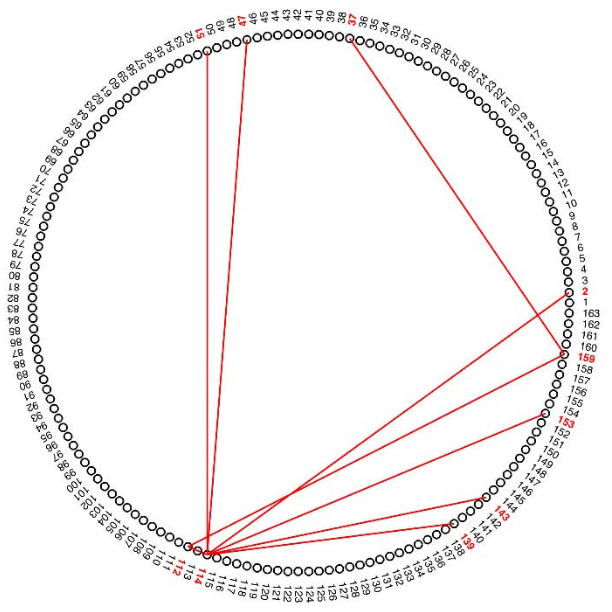

Positive Problem Orientation

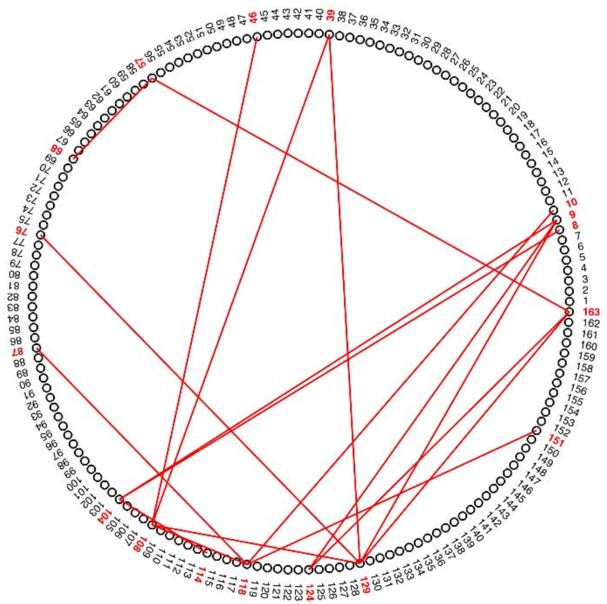

Negative Problem Orientation

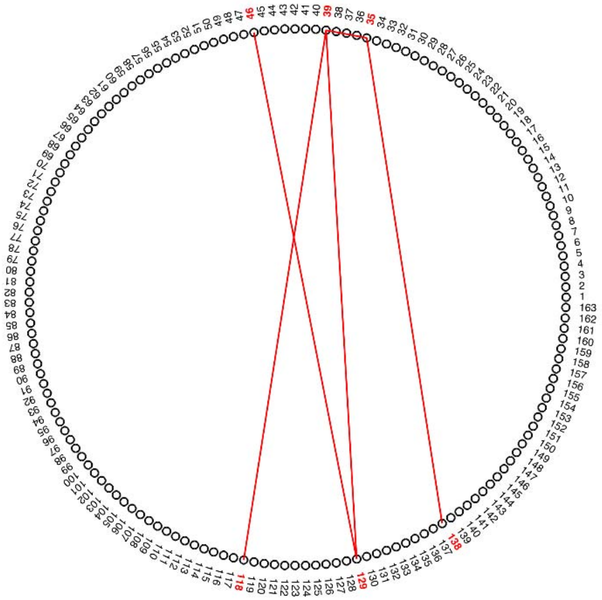

Problem Definition and Formulation

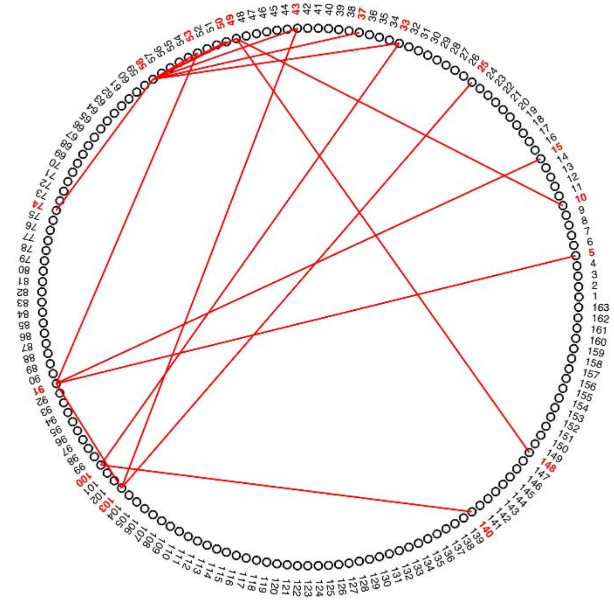

Generation of Alternative Solution

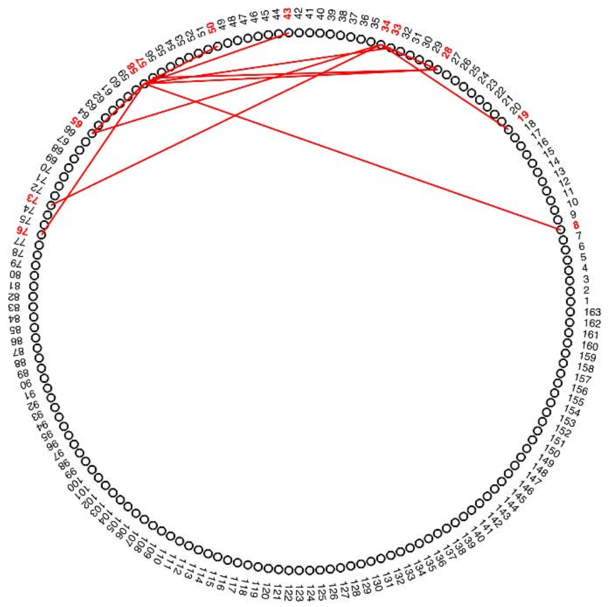

Decision Making

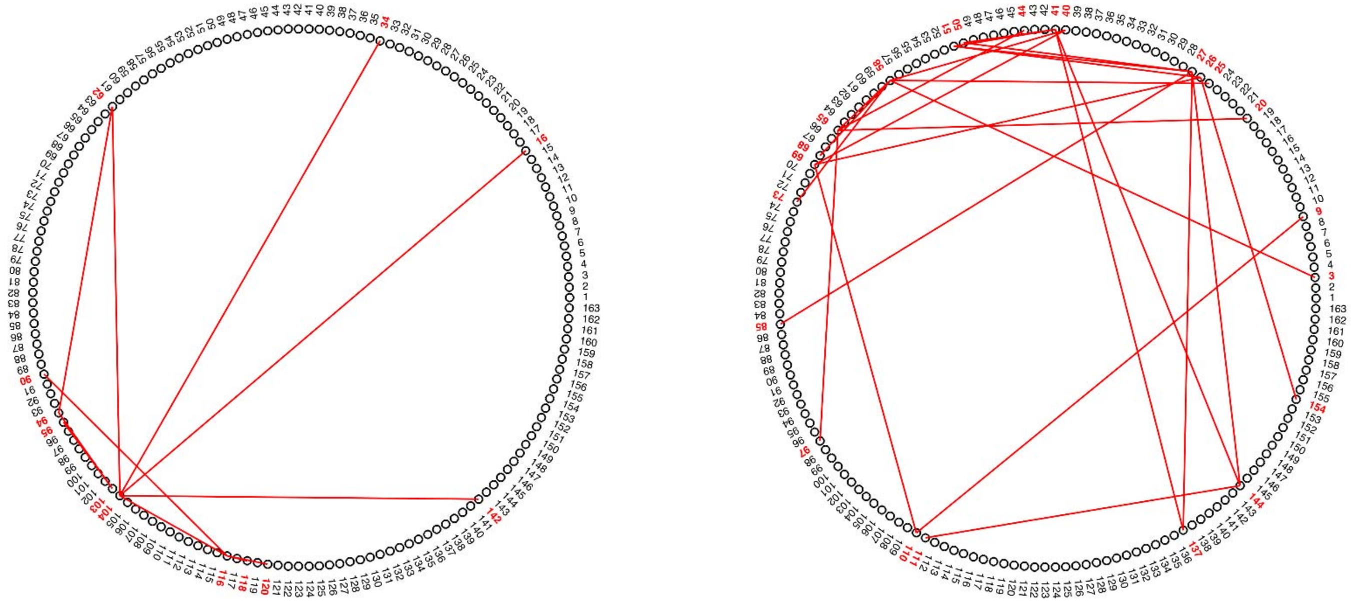

Solution Implementation and Verification

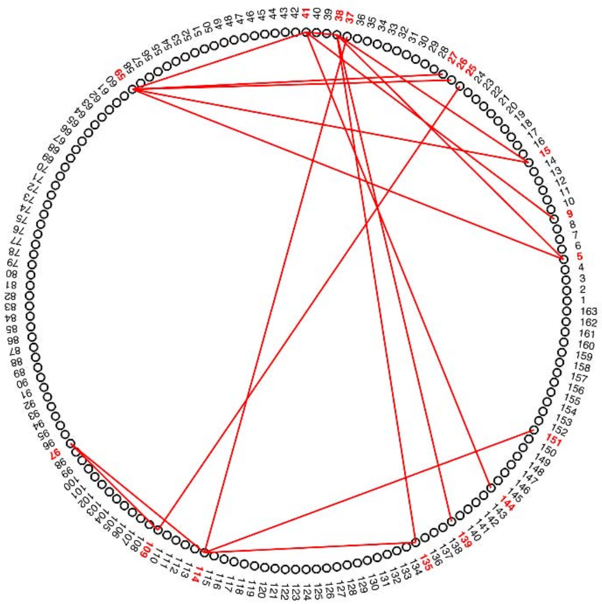

Rational Problem Solving

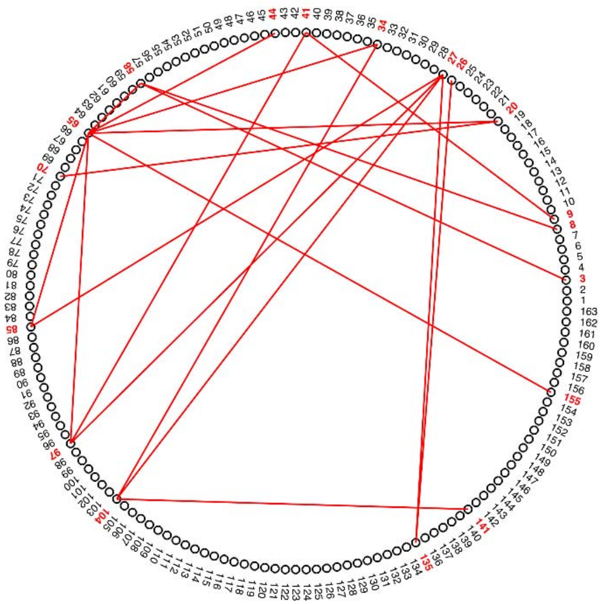

Impulsivity / Carelessness Style

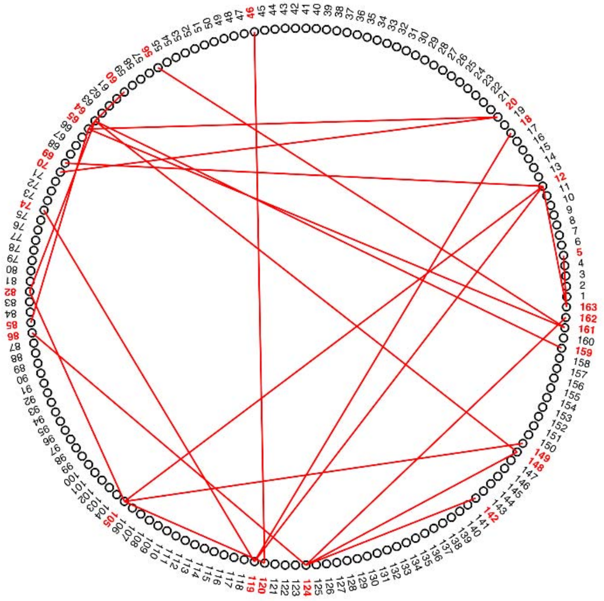

Social Problem-Solving Inventory-Revised(SPSI-R) Scale

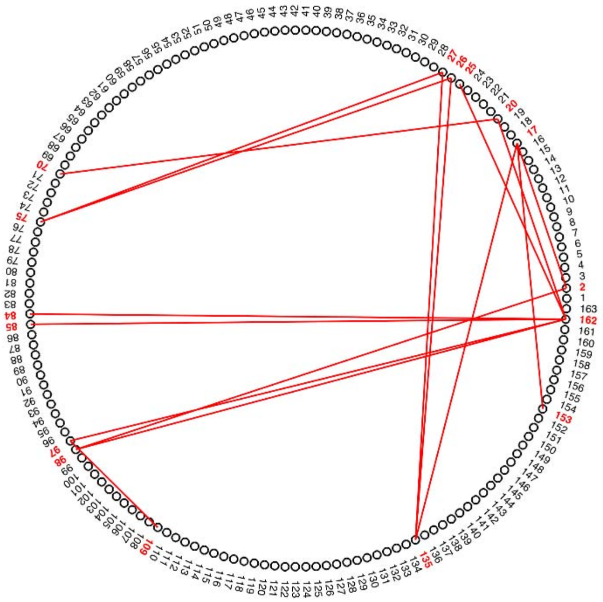

Avoidance Style

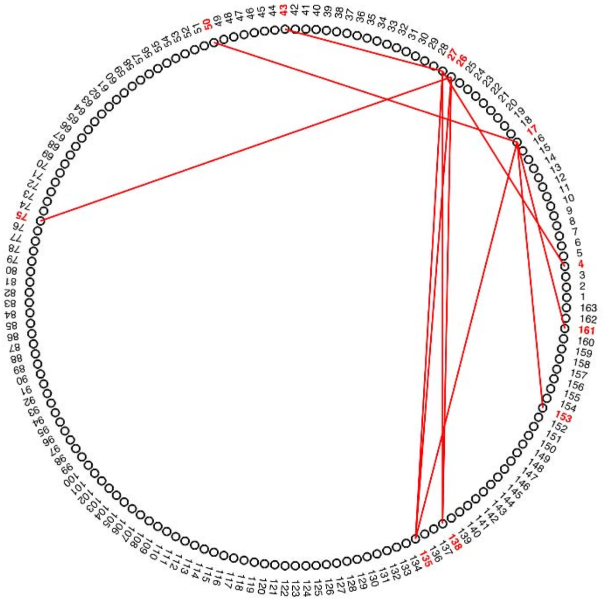

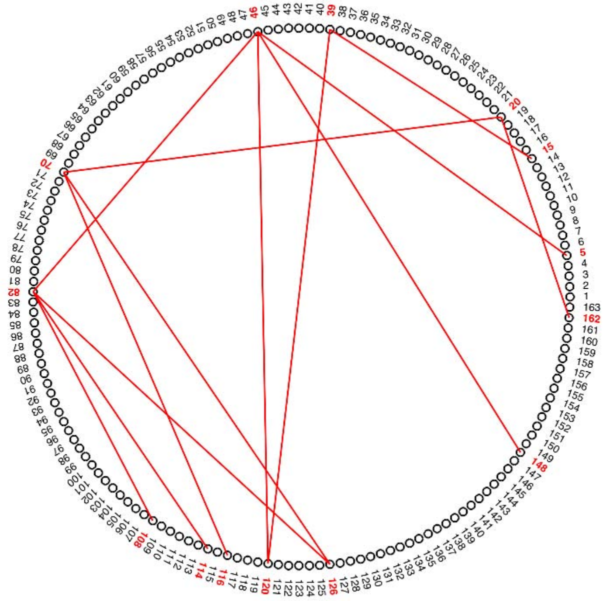

Negative-Self(NS)

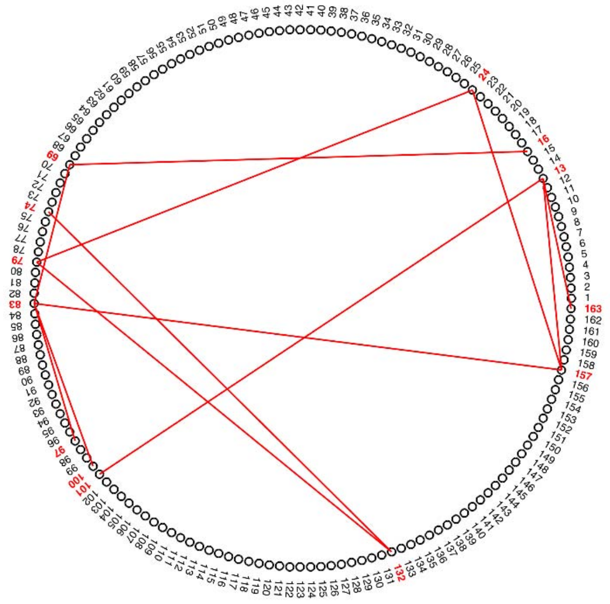

Positive-Self (PS)

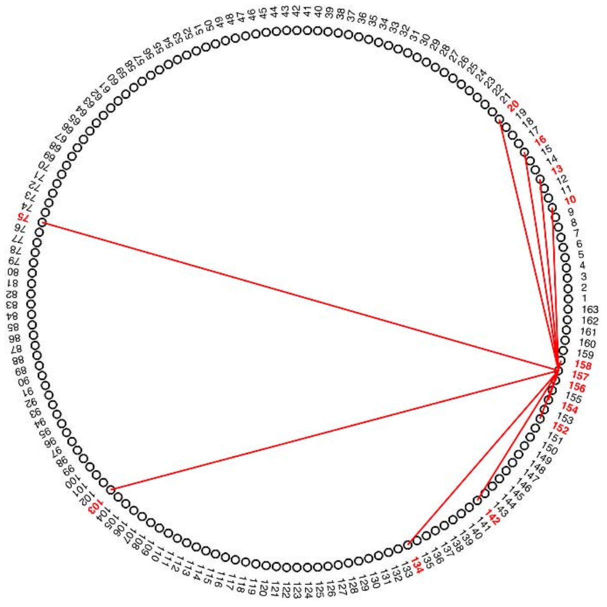

Negative-Other (NO)

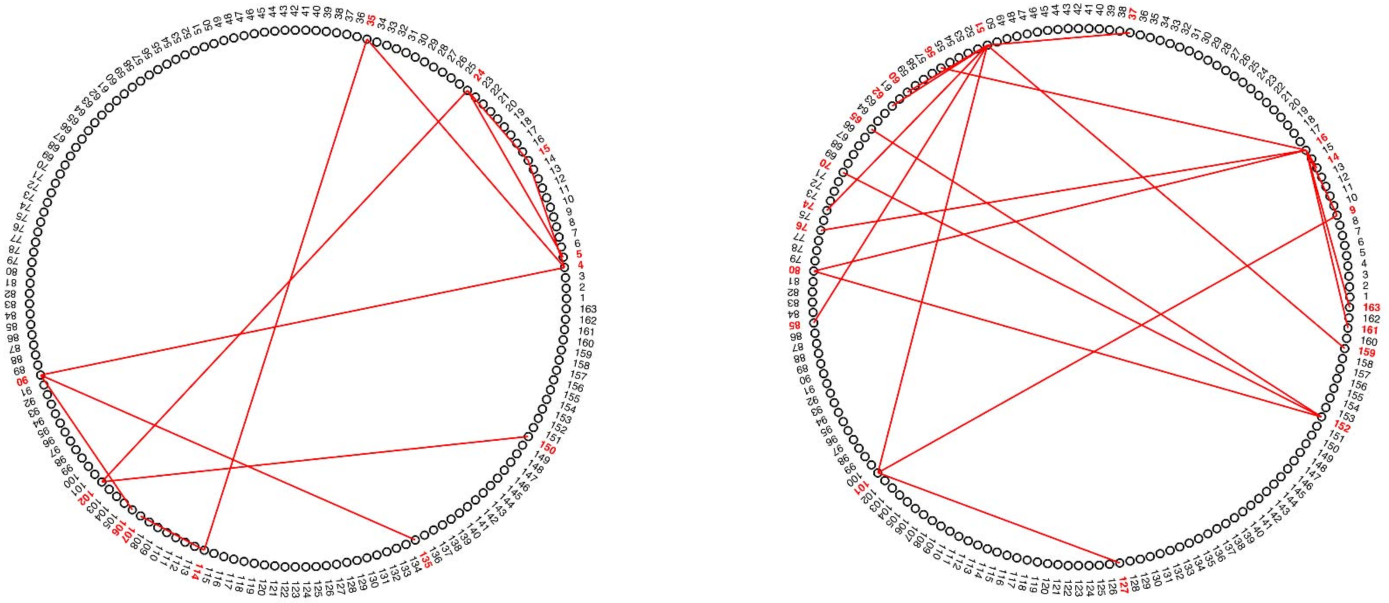

Positive-Other (PO)

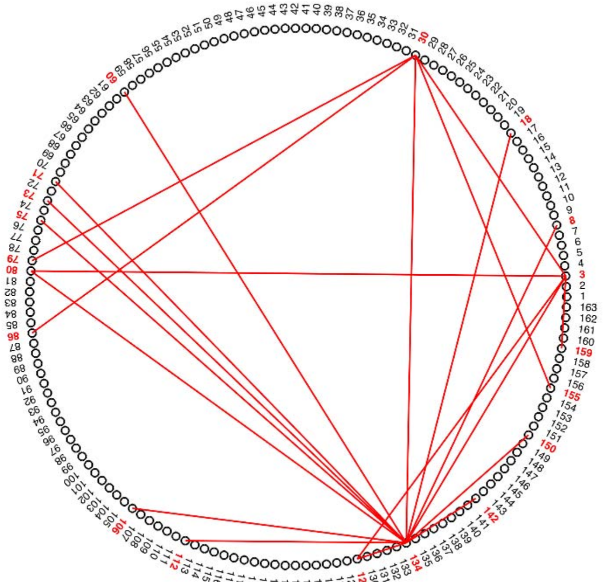

Brief Core Schema Scale

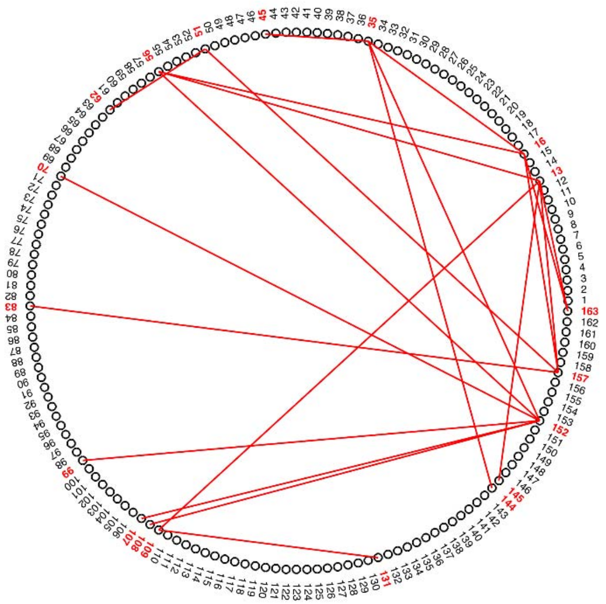

Intentional behavior

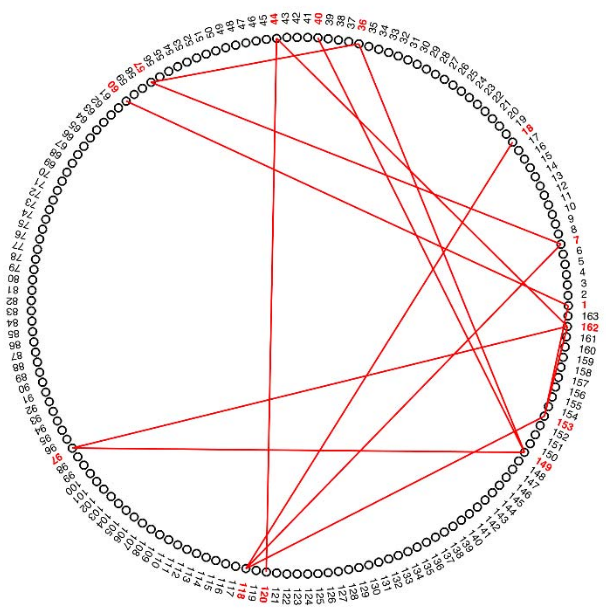

Planfulness

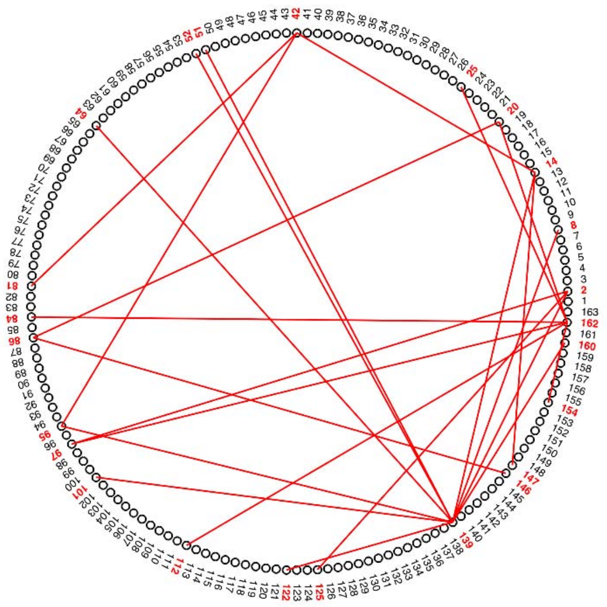

Readiness for change

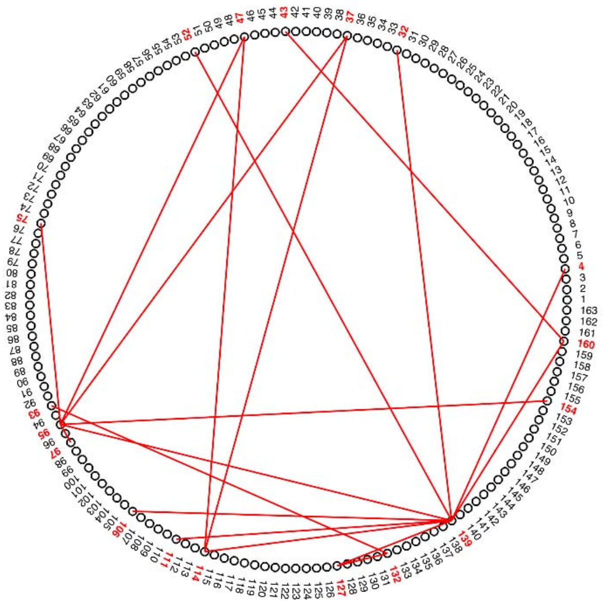

Using resource

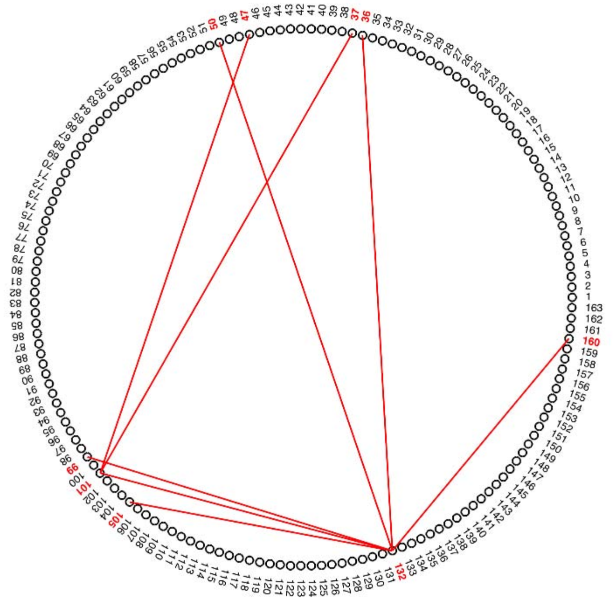

Personal Growth Initiative Scale-II

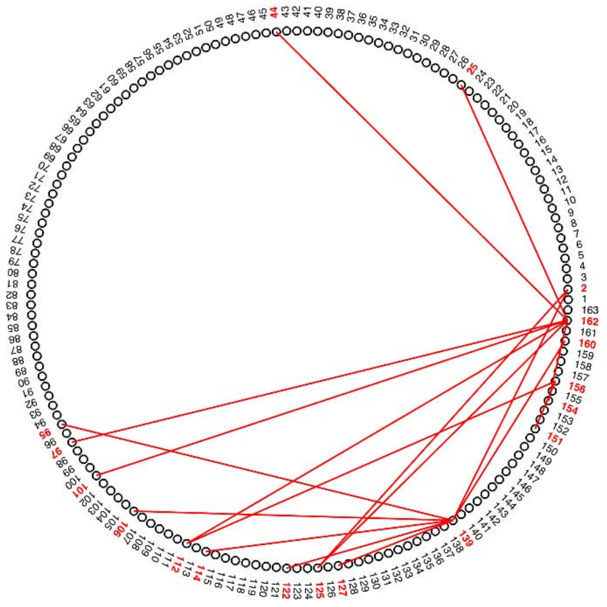

Subjective Happiness Scale: SHS

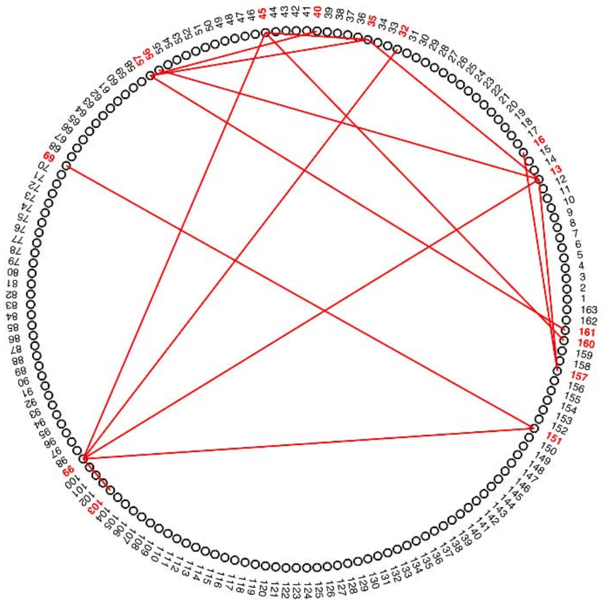

The Satisfaction with Life Scale (SWLS)

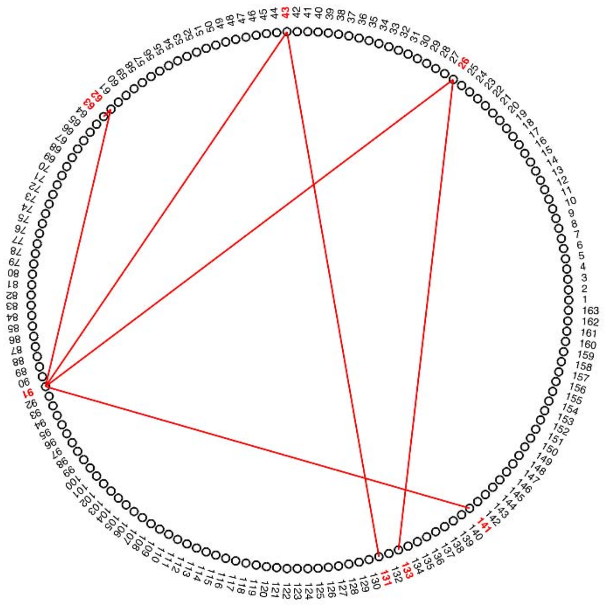

State Anxiety (A -State)

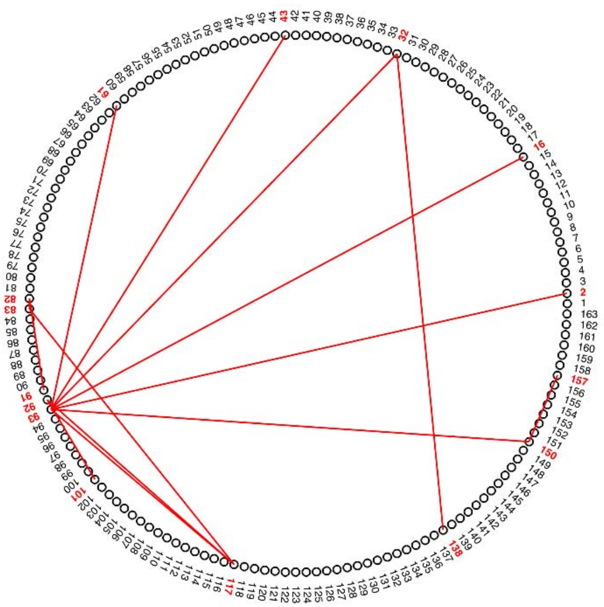

Trait Anxiety (A-Trait)

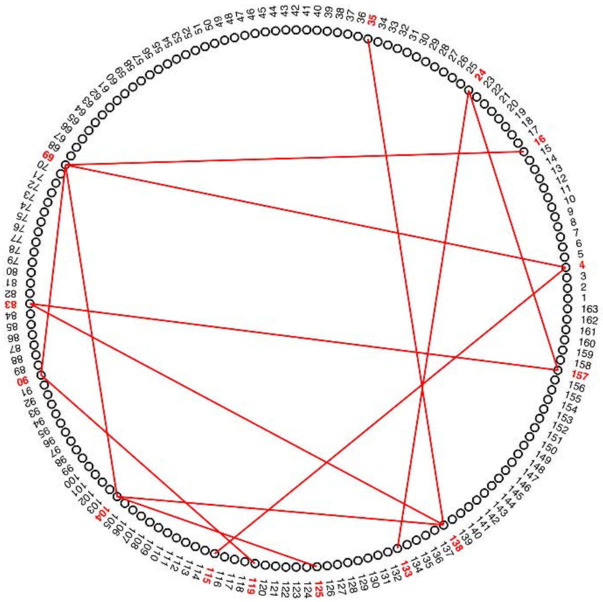

The State-Trait Anxiety Inventory

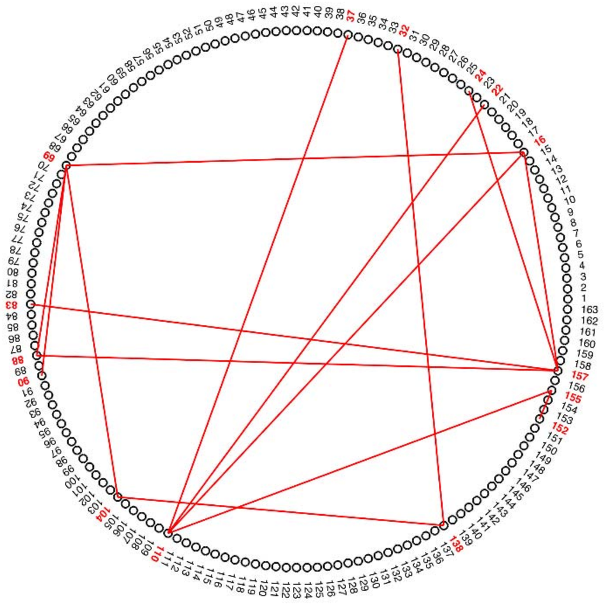

Positive symptoms

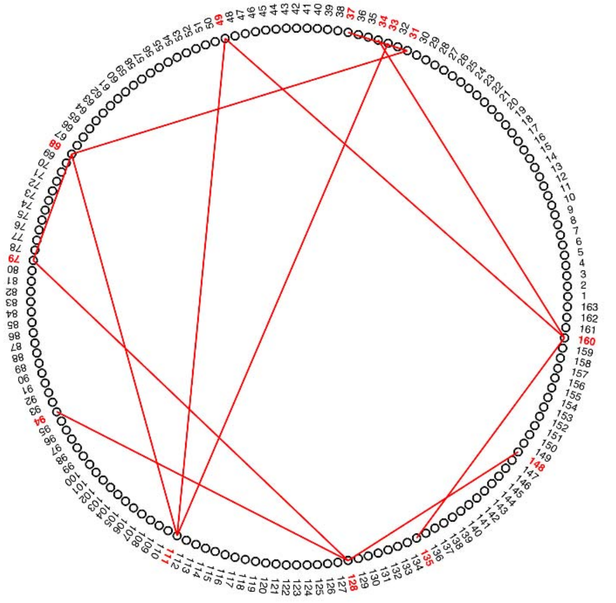

Negative symptoms

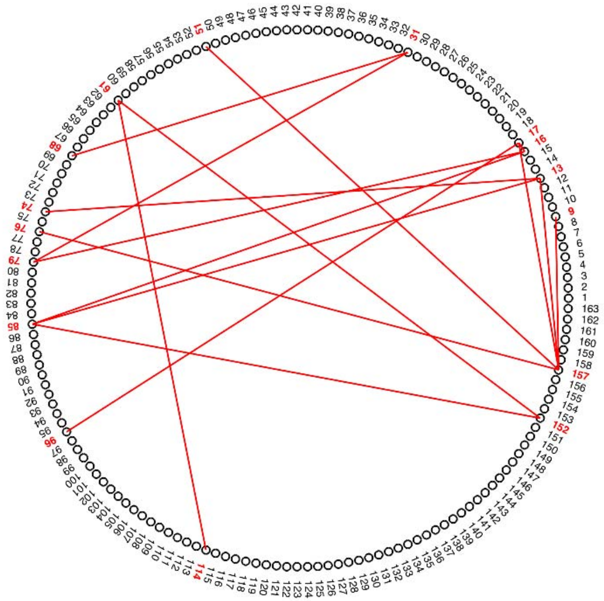

Disorganization

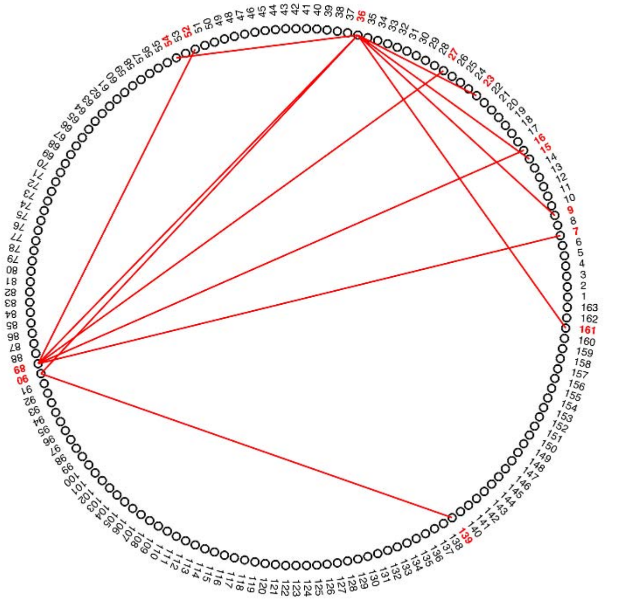

Schizotypal Personality Questionnaire-Brief

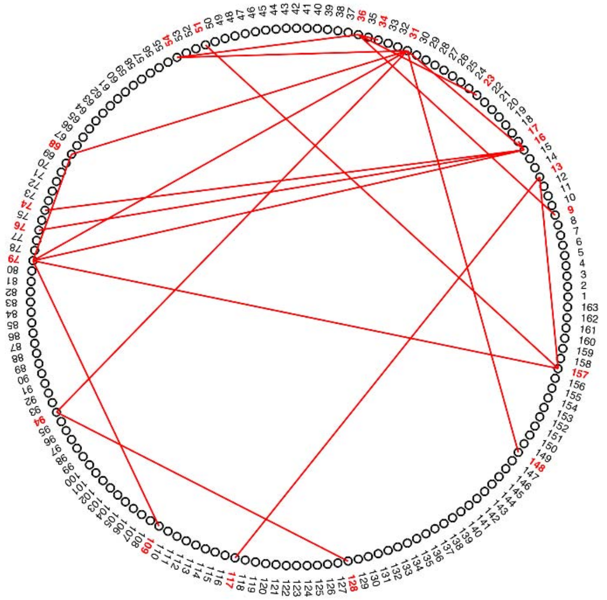

Social skill

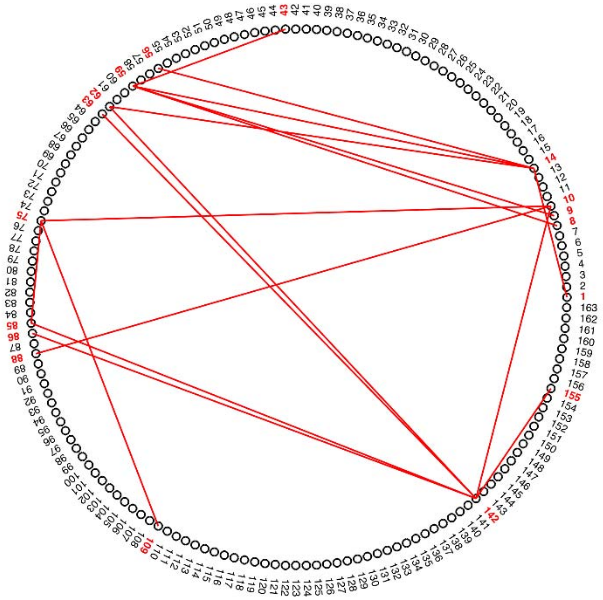

Attention switching

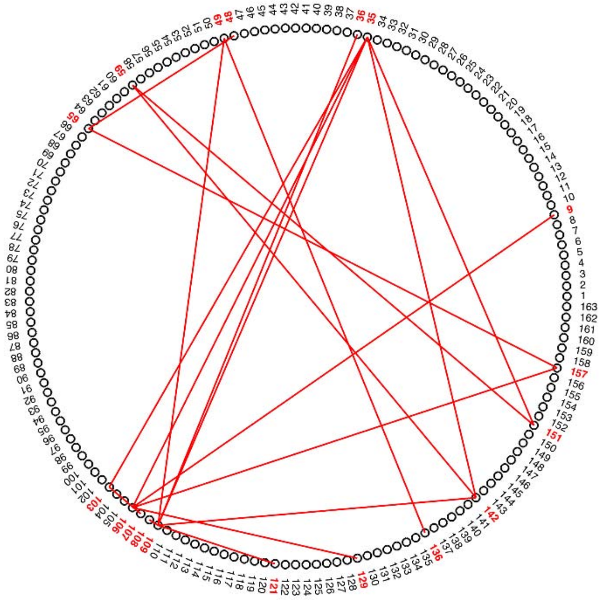

Attention to detail

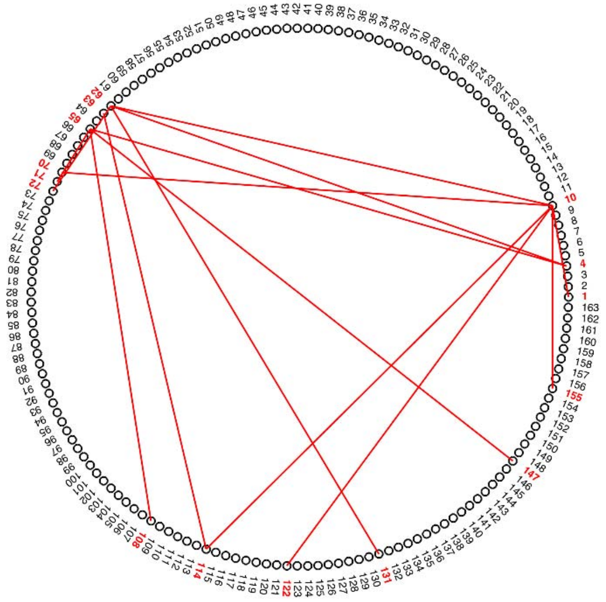

Communication

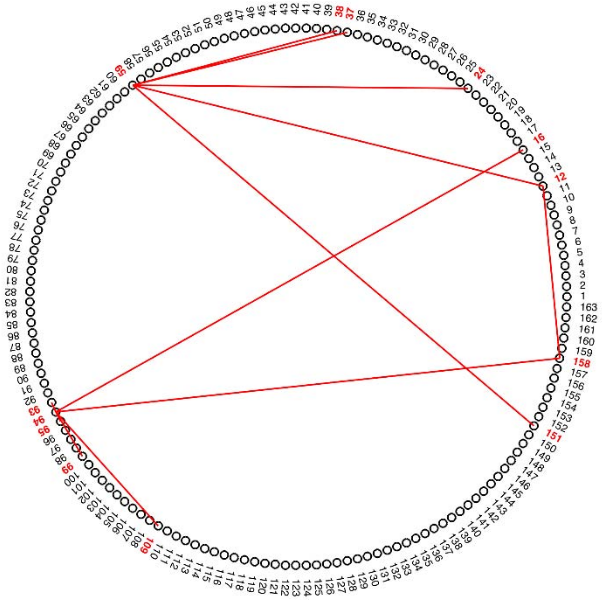

Imagination

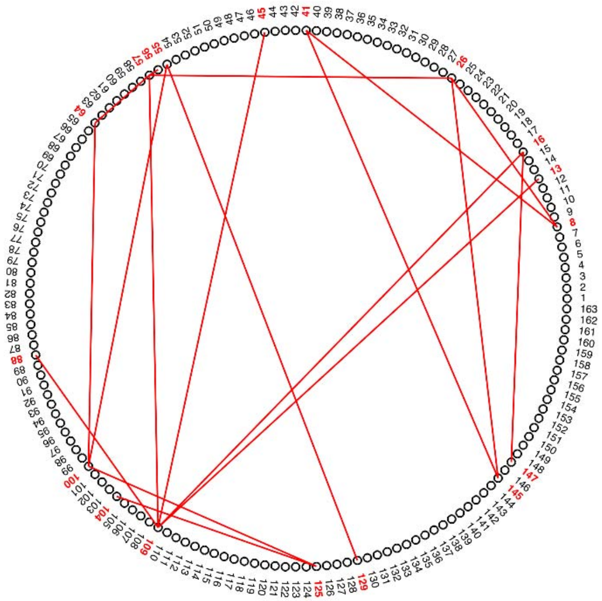

Autism-Spectrum Quotient

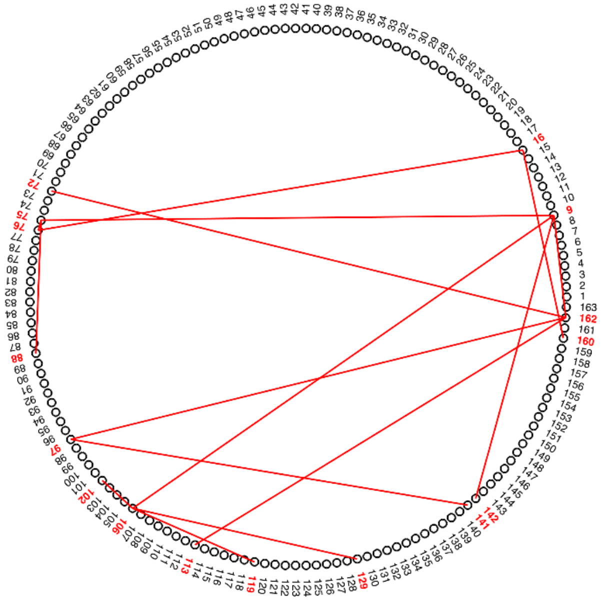

IQ Paras

Verbal IQ

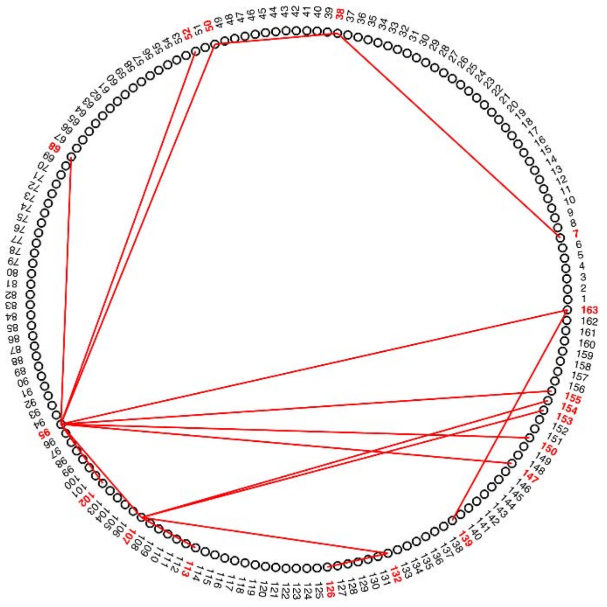

Performance IQ

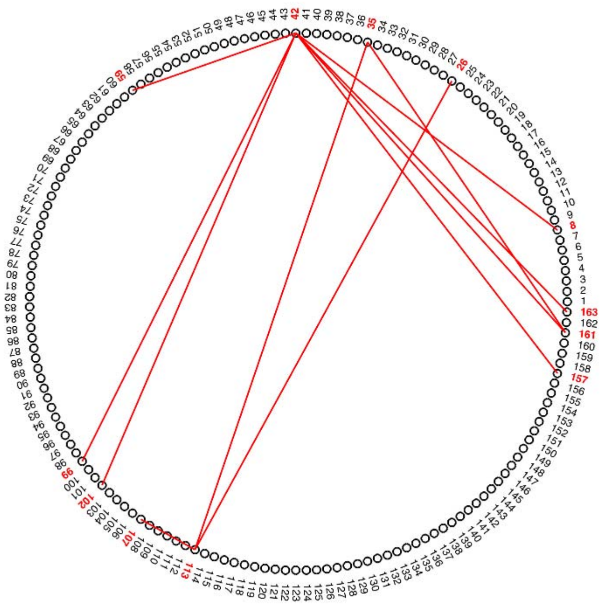

Full scale IQ

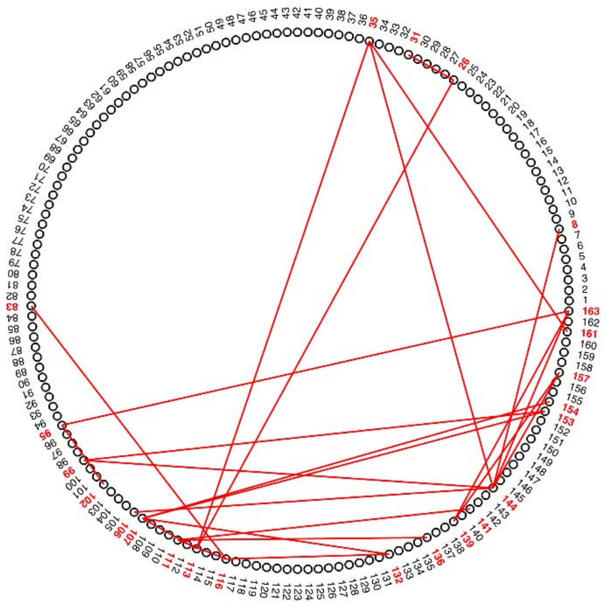

Comprehension

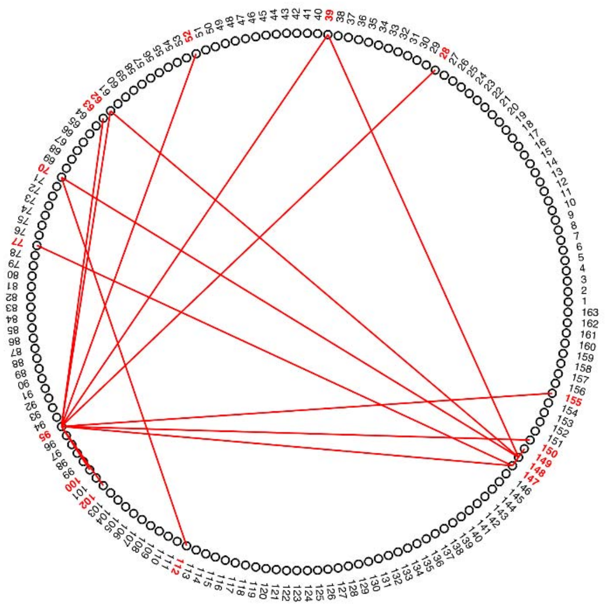

Crystallized intelligence

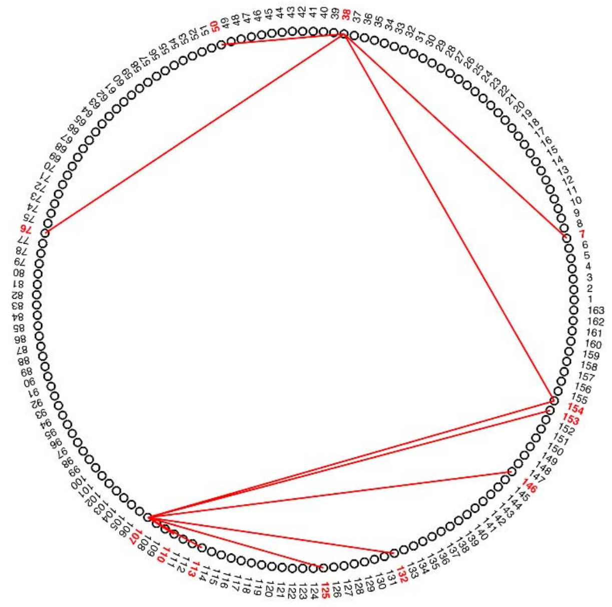

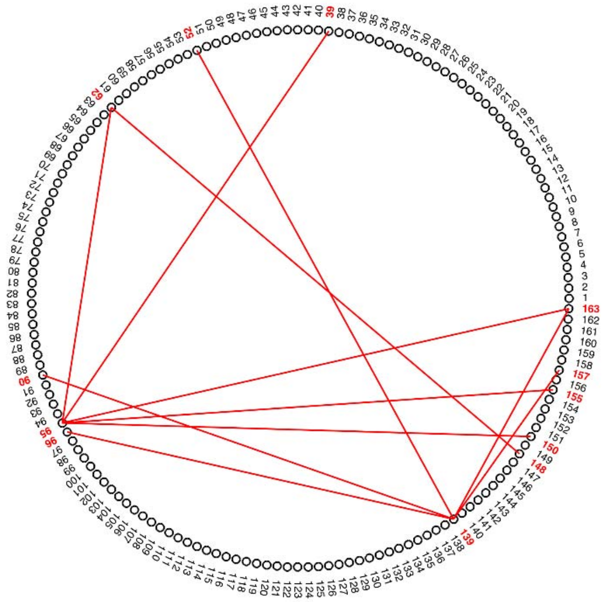

Perceptual Organization

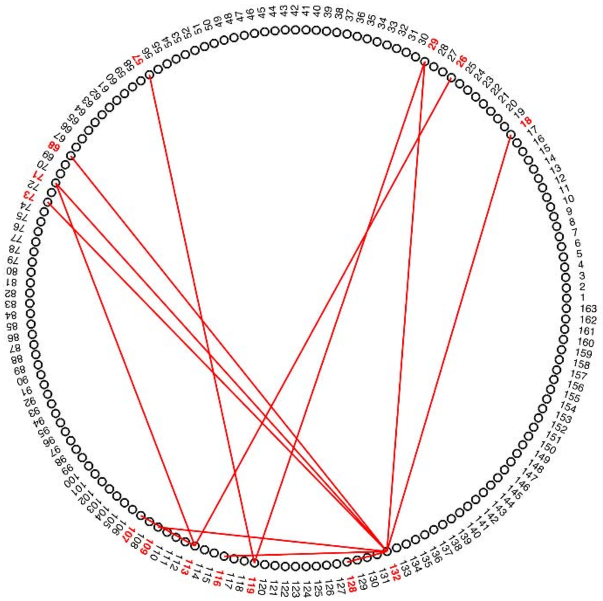

Similarities

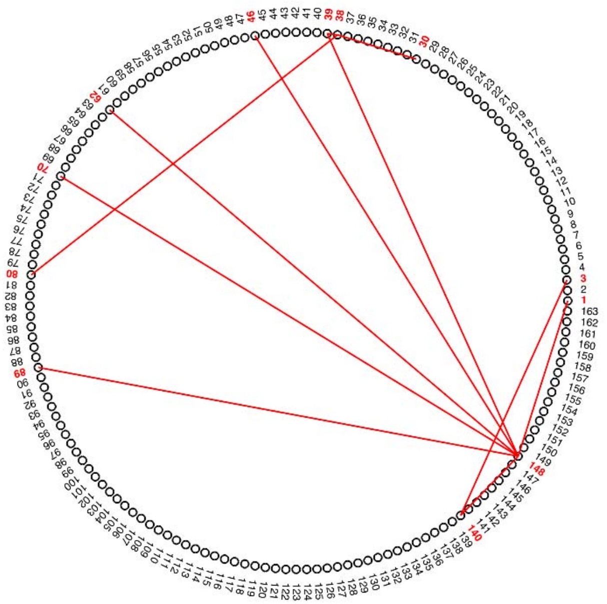

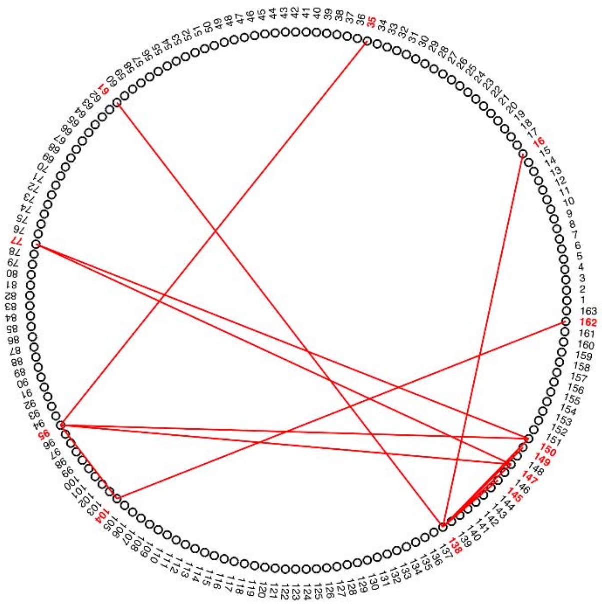

Arithmetic

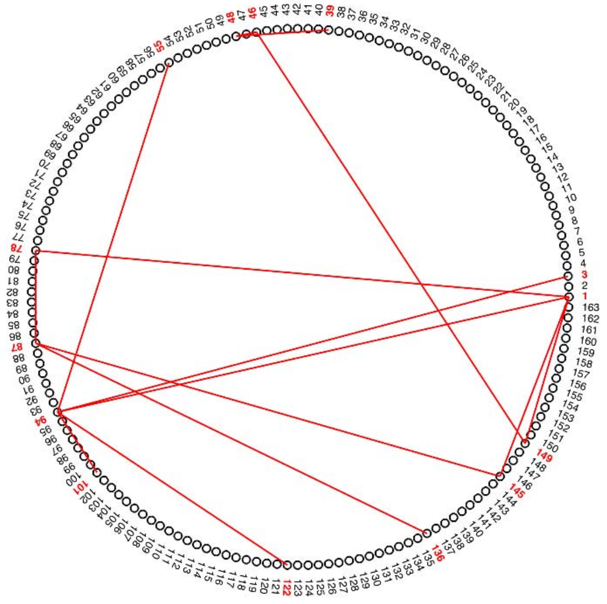

Digit Span

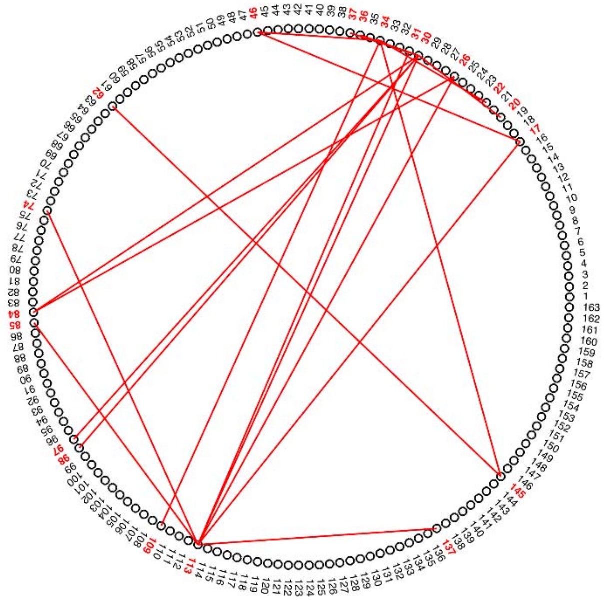

Information

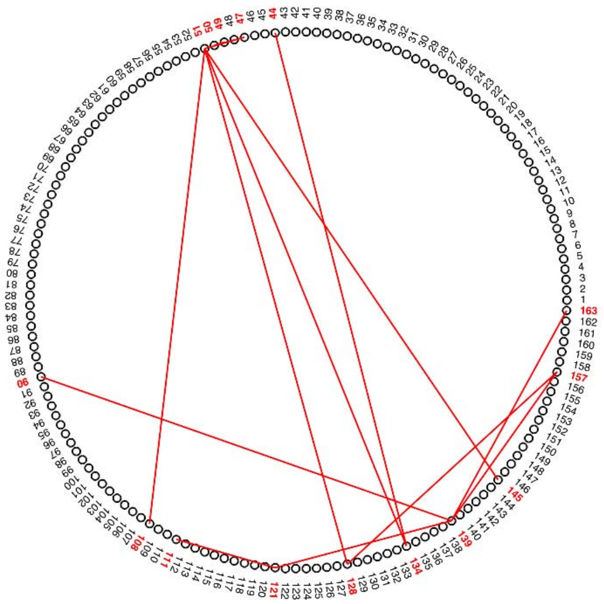

Comprehension

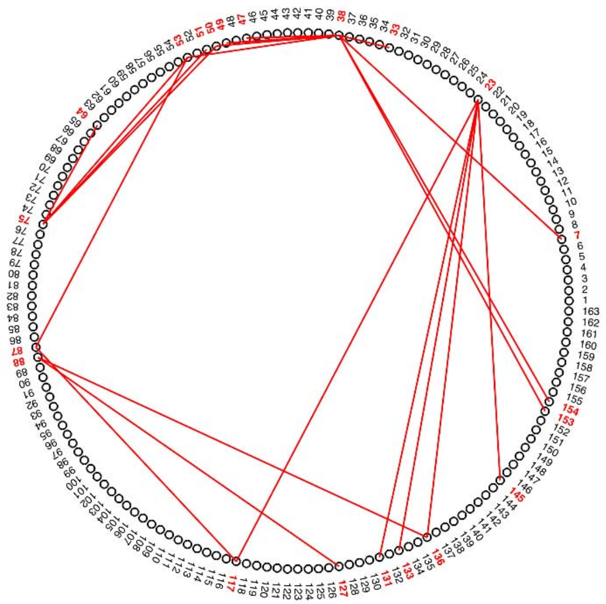

Digit Symbol

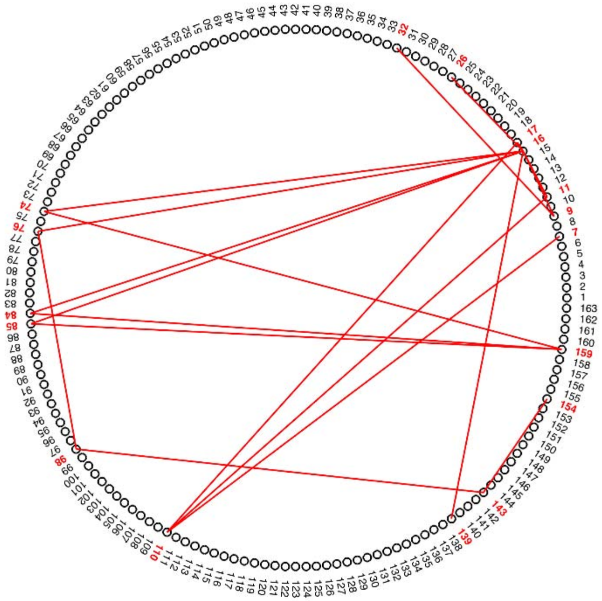

Block Design

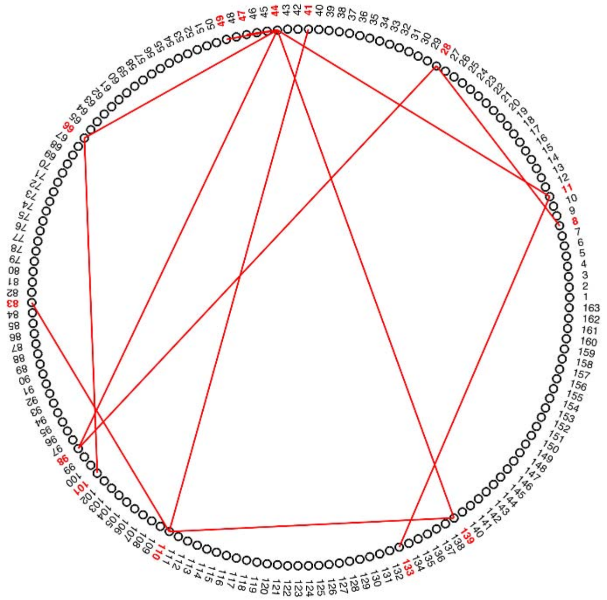

Matrix Reasoning

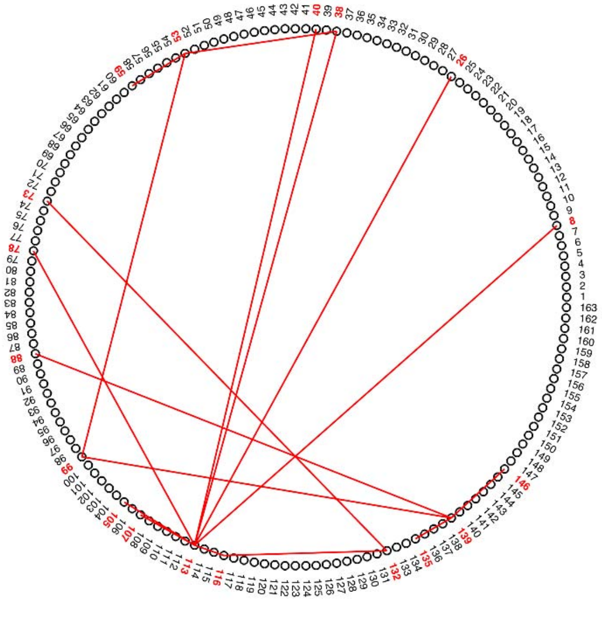

Picture Arrangement

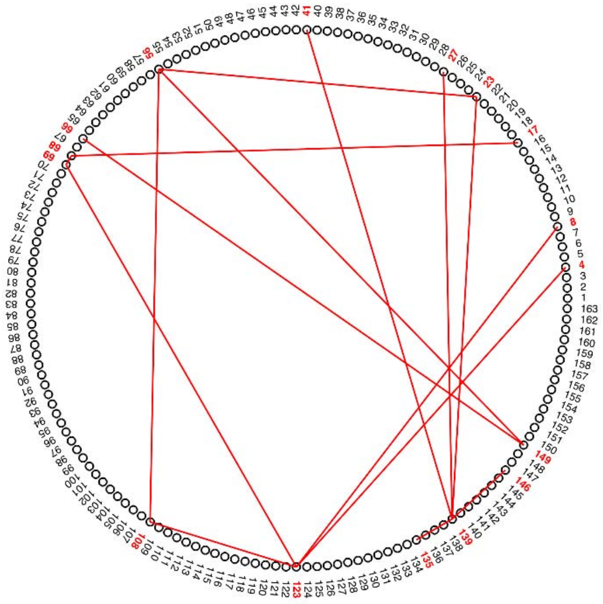

EQ Paras

Emotional awareness

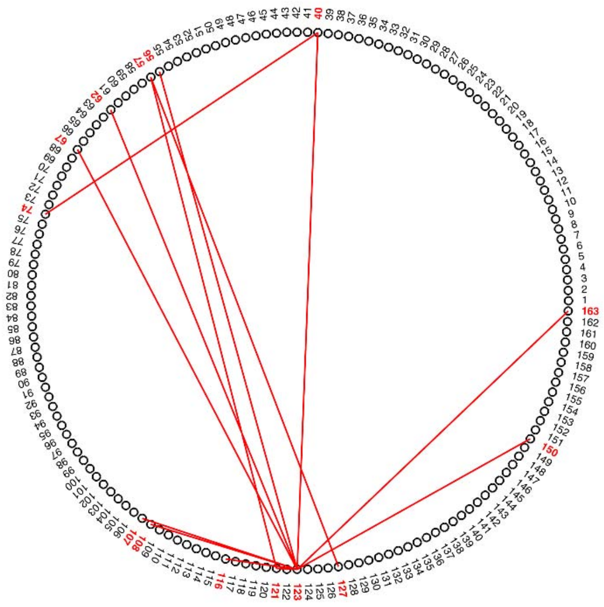

Self-efficacy

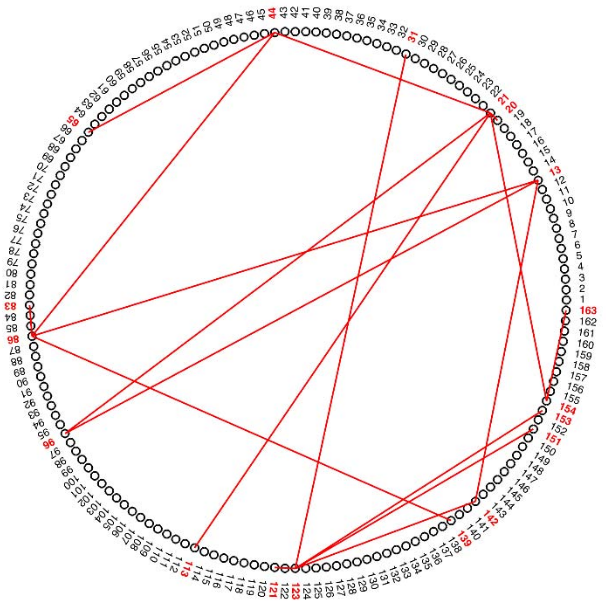

Perseverance

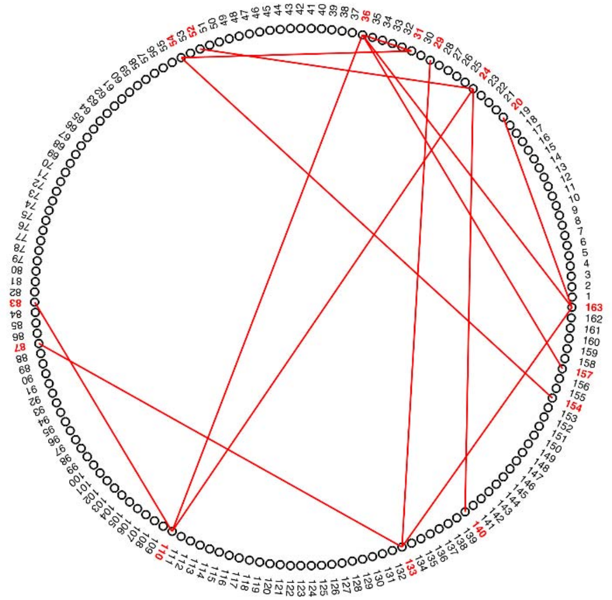

Enthusiasm

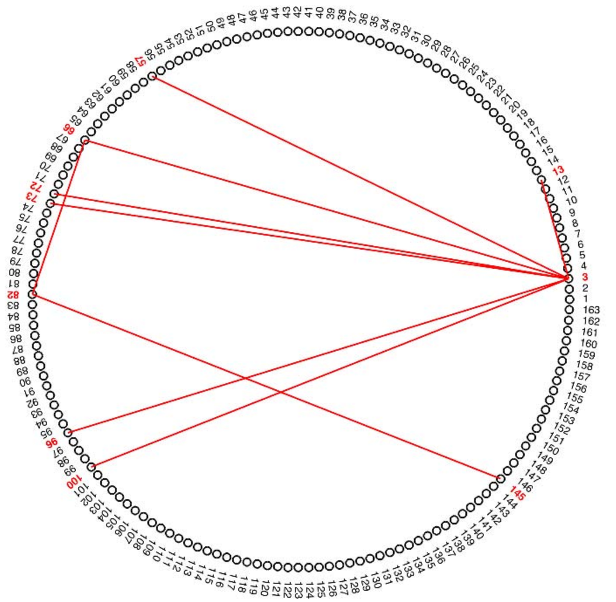

Self-decision

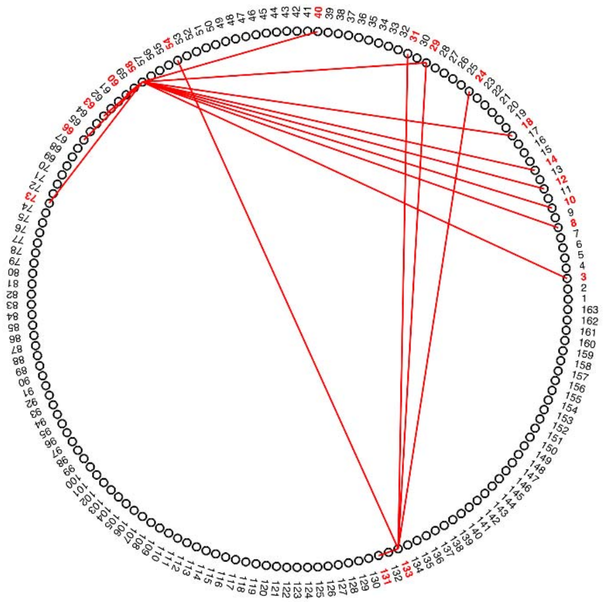

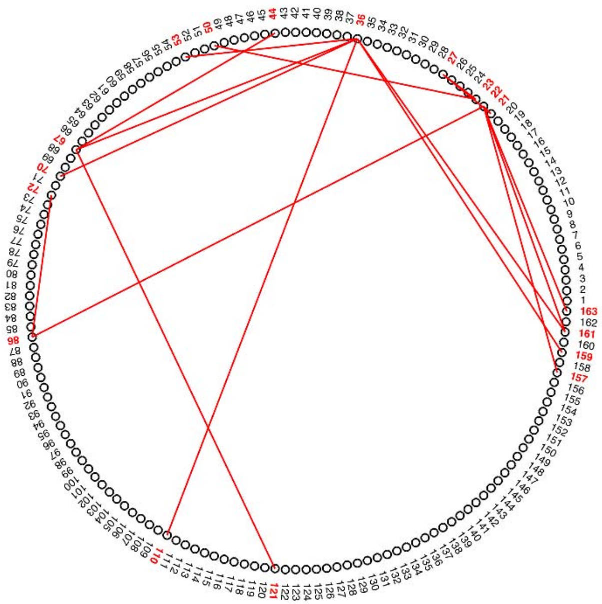

Impulse control

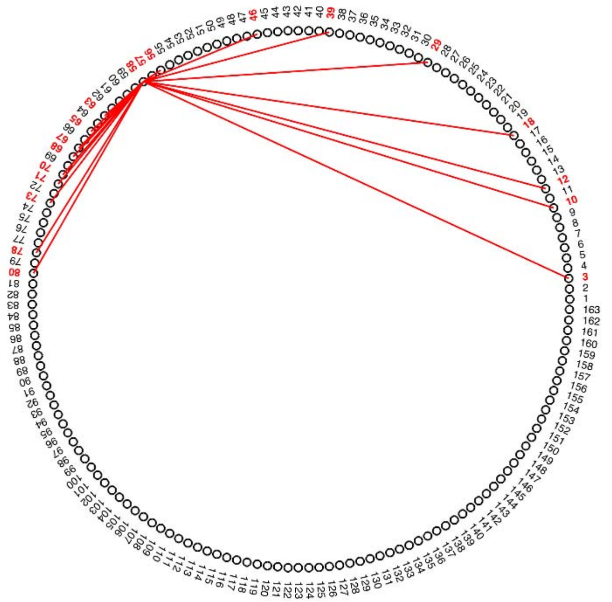

Sharing positive emotion

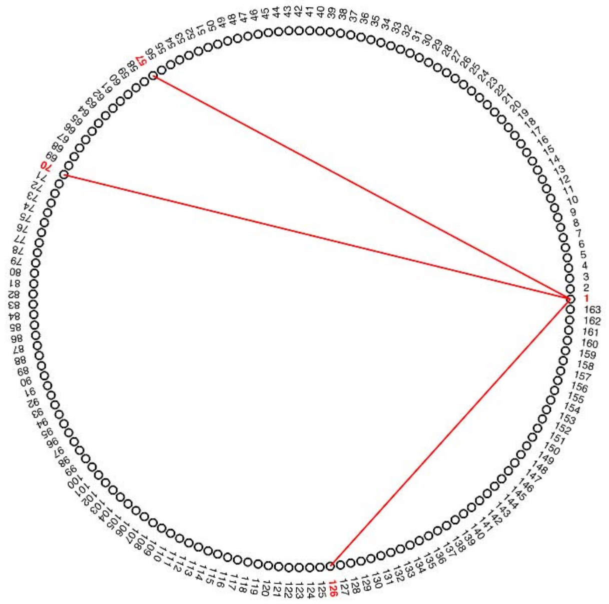

Sharing negative emotion

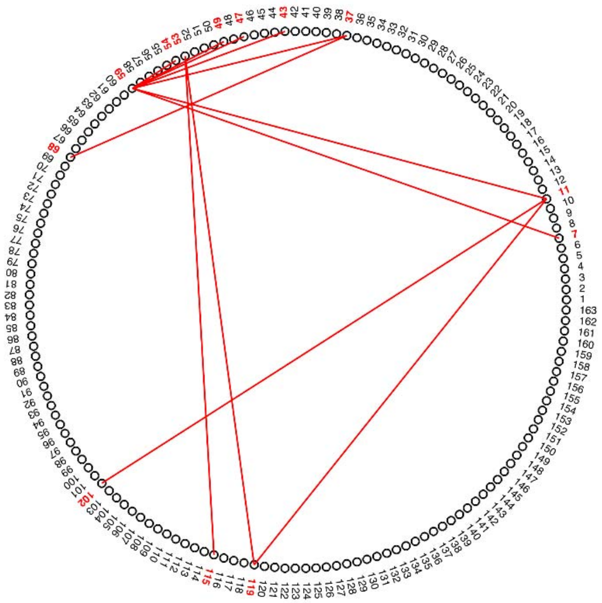

Sociability

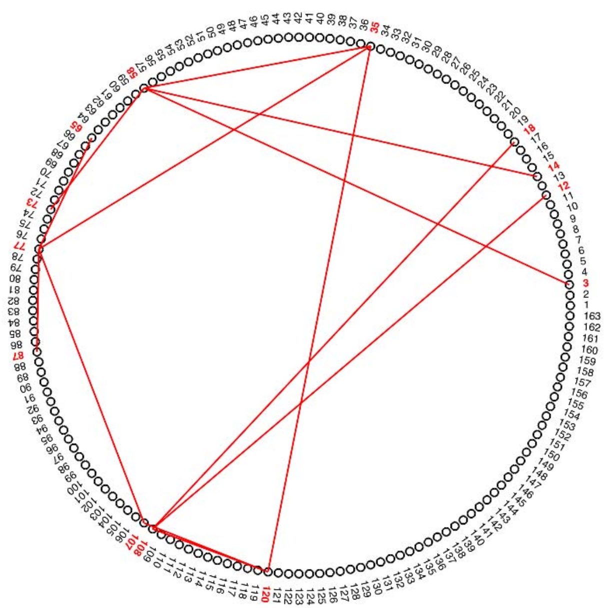

Cooperation

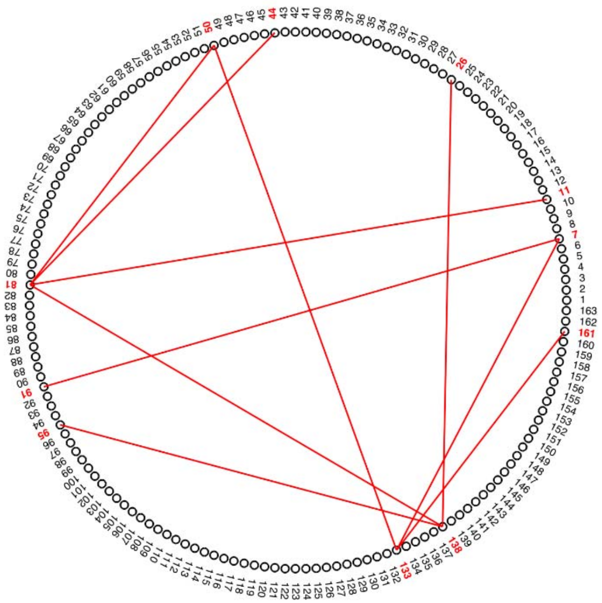

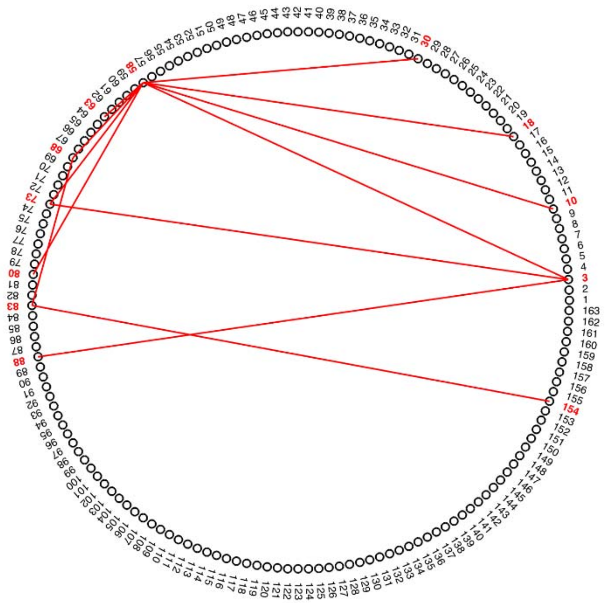

Decision making

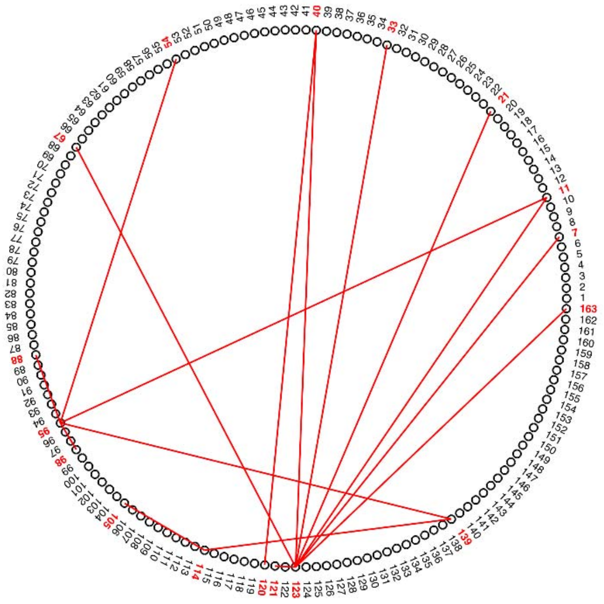

Optimism

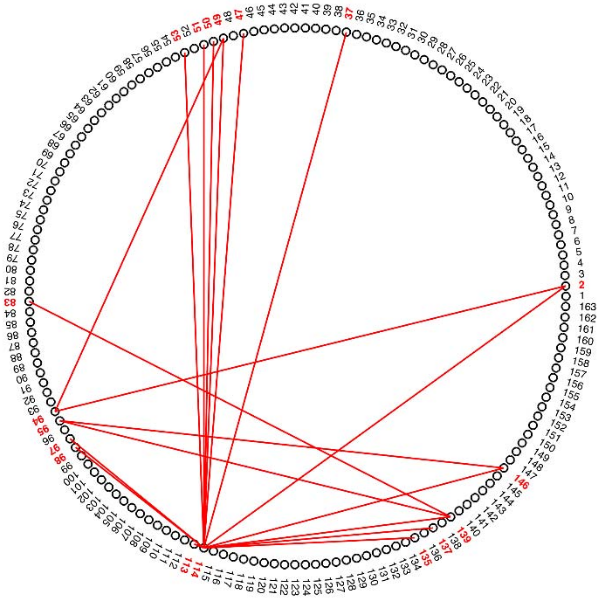

Group consideration

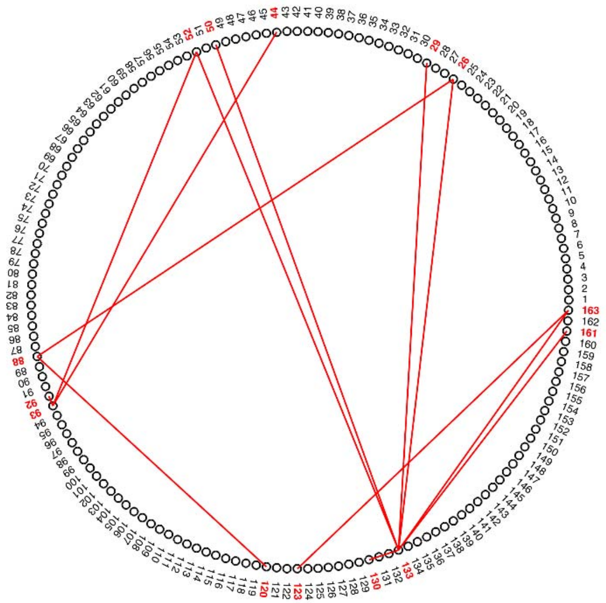

Influence

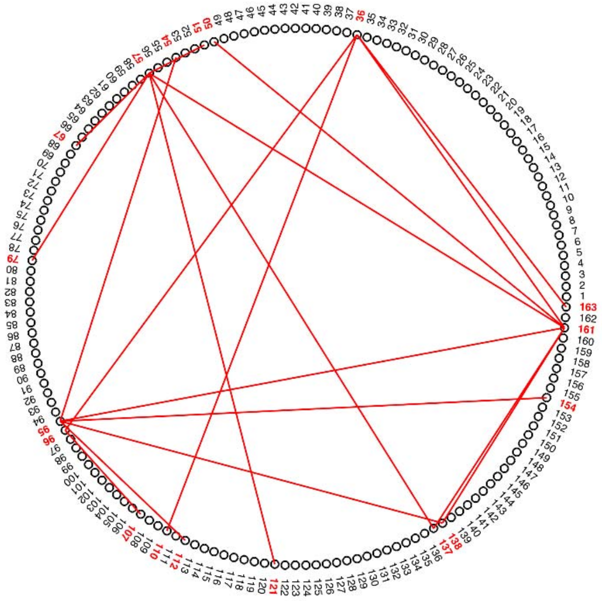

Risk management

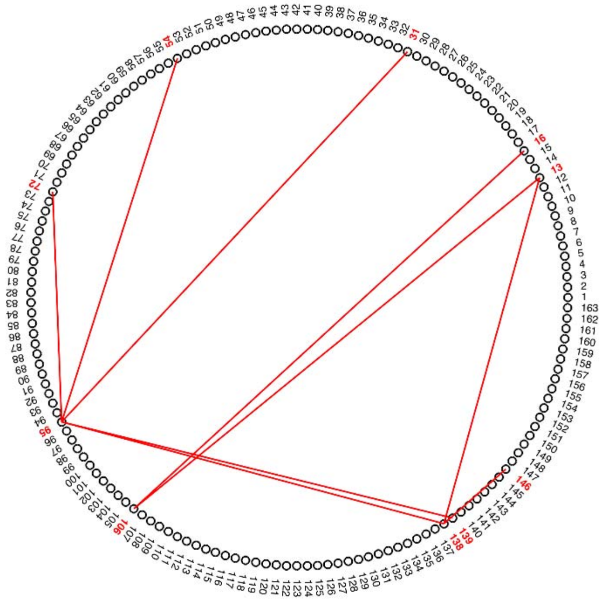

Tactfulness

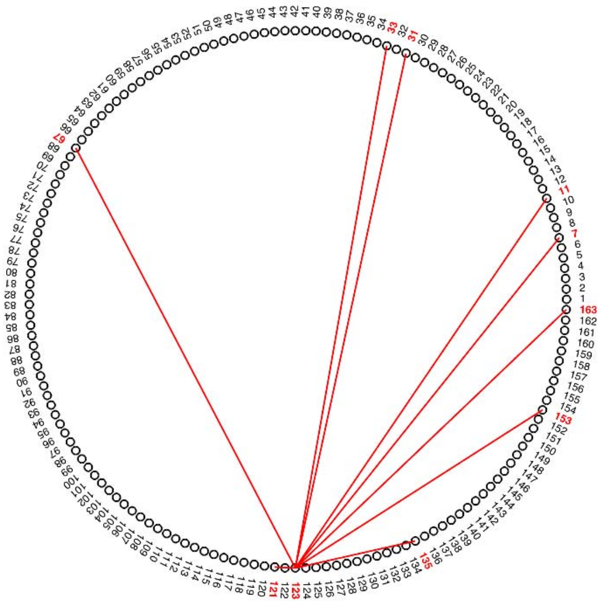

Adaptability

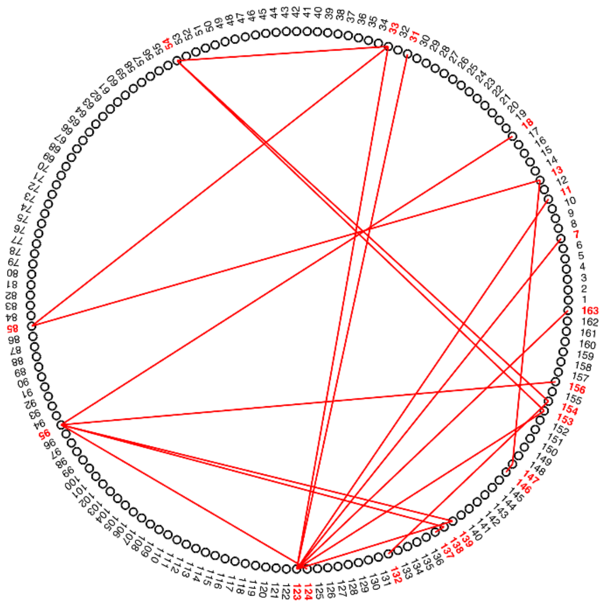

Self-awareness

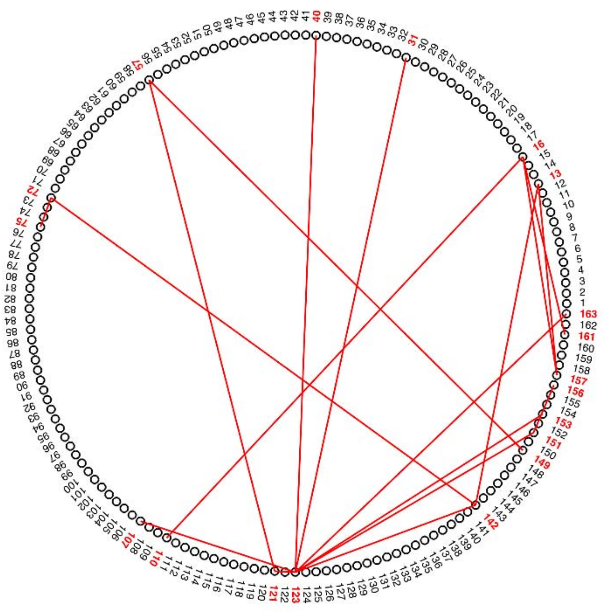

Self-control

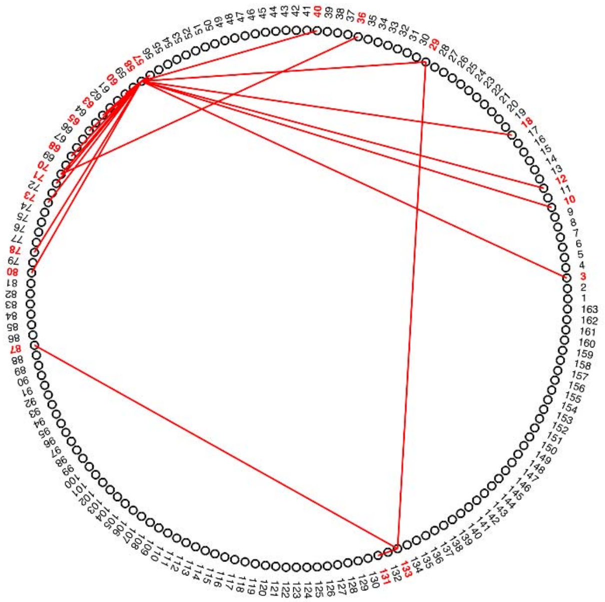

Interpersonal relationship

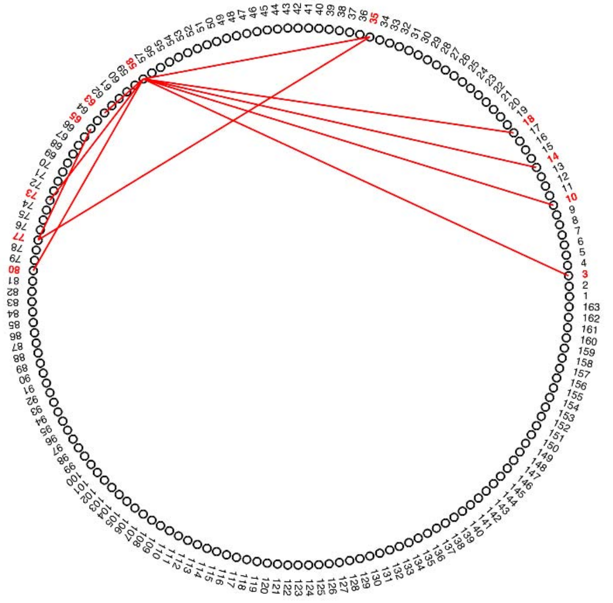

Situational awareness

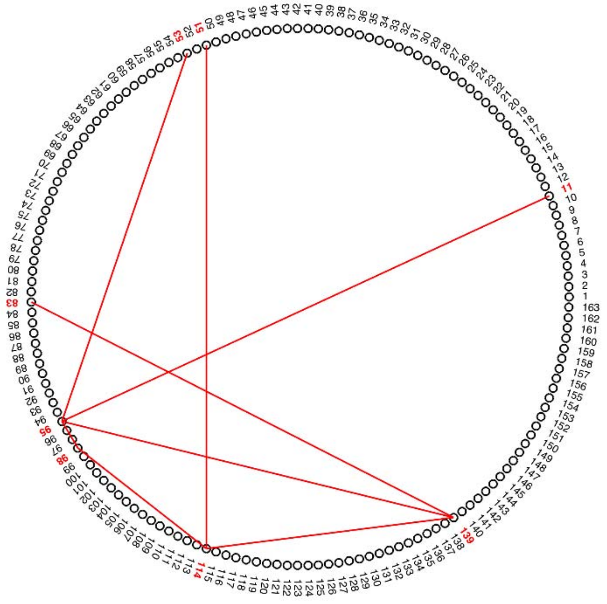

Leadership

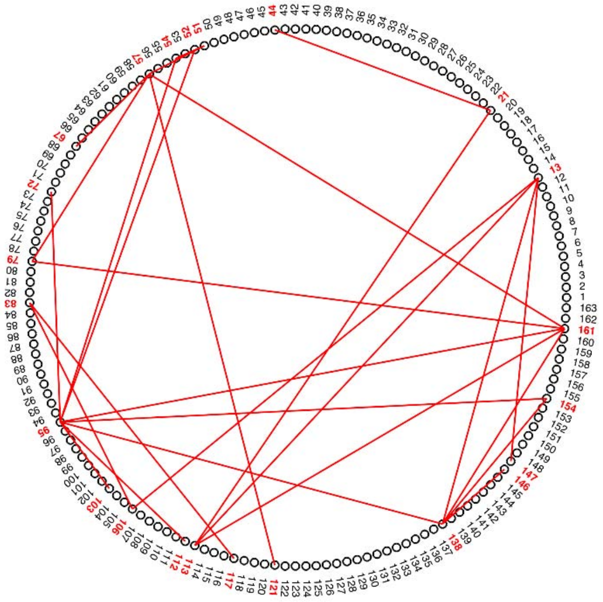

Flexibility

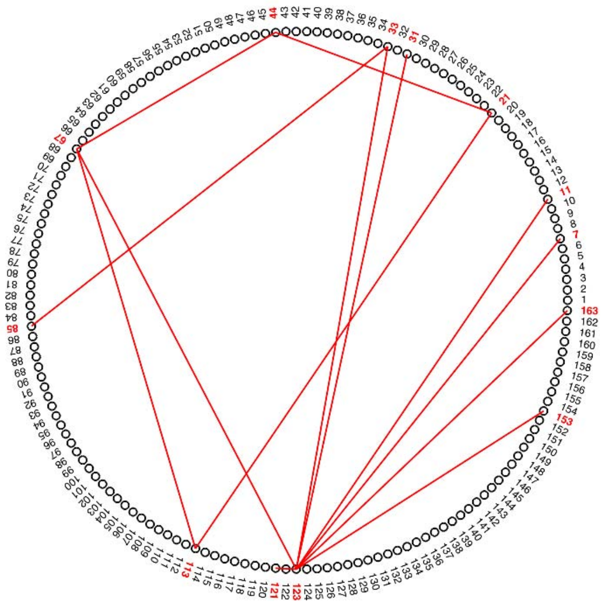

Intrapersonal EQS

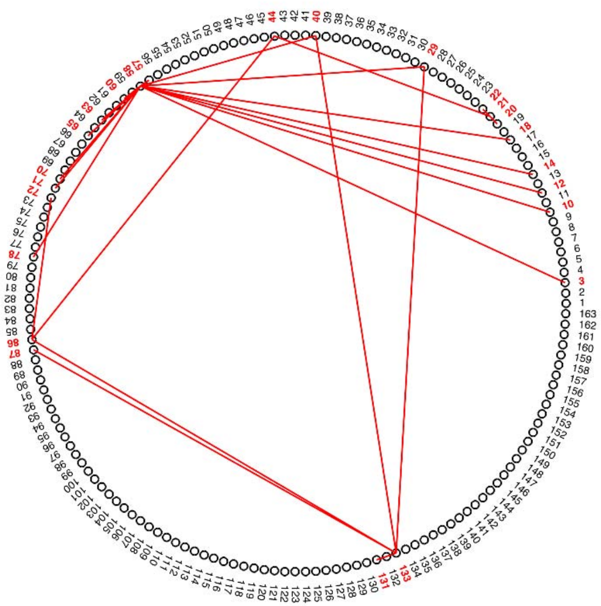

Interpersonal EQS

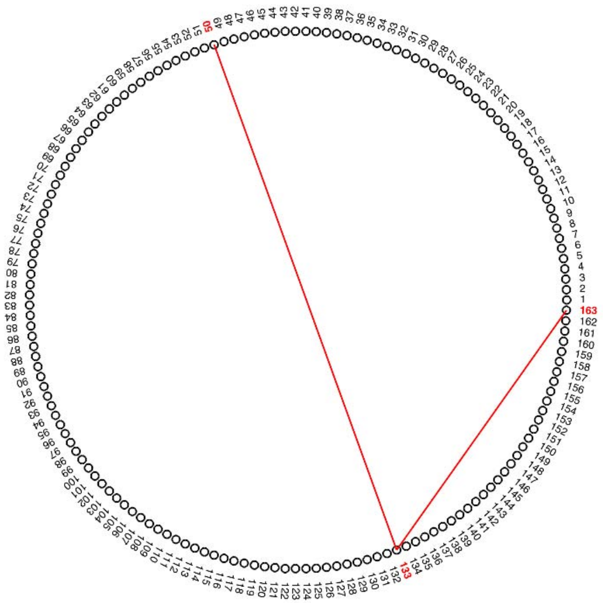

Situational EQS

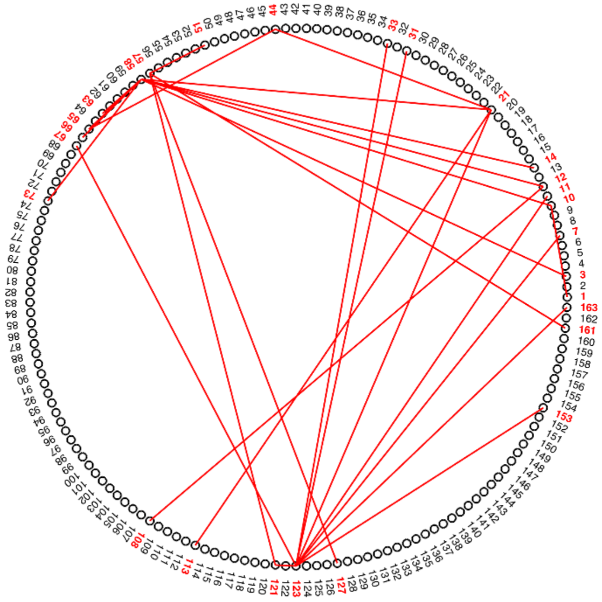

EQS total scale

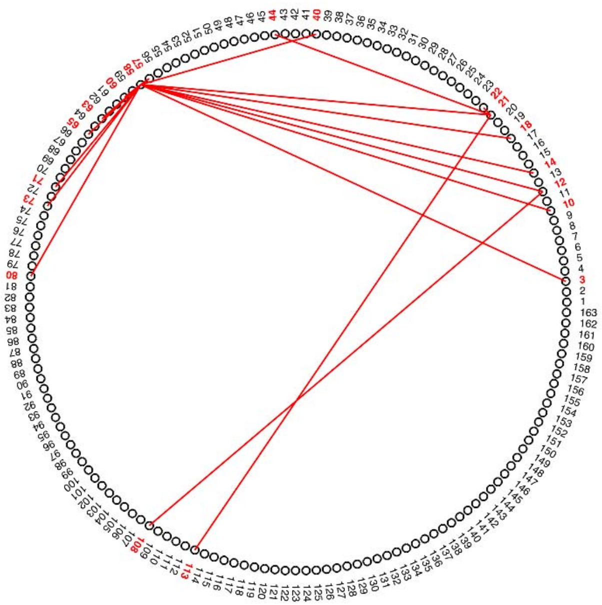

Supplement: Figure S1 — Set of all functional networks. [file Image1.PDF]
