# Supplementary material for: A Set of Functional Brain Networks for the Comprehensive Evaluation of Human Characteristics
Source: Front Neurosci. 2018 Mar 14;12:149. doi: 10.3389/fnins.2018.00149 (PMC5861187; doi:10.3389/fnins.2018.00149)
Supplement: Figure S2 — Correlation between each functional network and its psychometric score for 70 functional networks with 153 subject data by a linear multiple regression based on the least square analysis. The average correlation value R is 0.51 (S.D 0.07) and R2 is 0.27 (S.D 0.07), p < 0.0001. [file Image2.PDF]

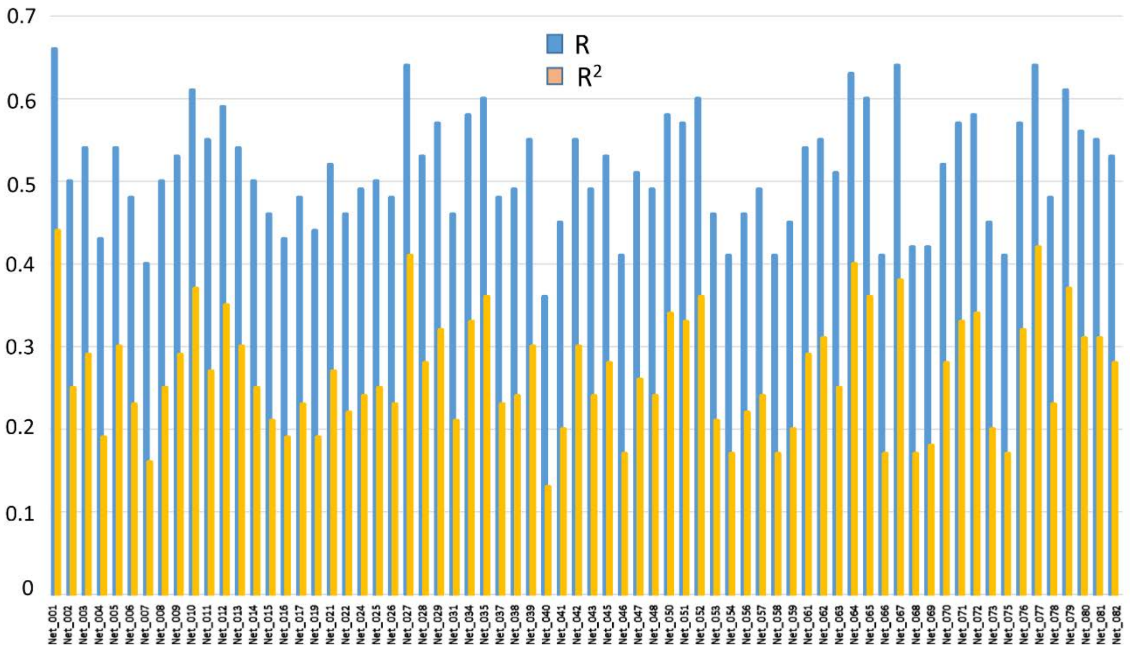

Supplementary Figure 2

Correlation between each functional network and its psychometric score for 70 functional networks with 153 subject data by a linear multiple regression based on the least square analysis. The average correlation value R is 0.51 (S.D 0.07) and R^2 is 0.27 (S.D 0.07), $p < 0.0001$.
