# Supplementary material for: A Set of Functional Brain Networks for the Comprehensive Evaluation of Human Characteristics
Source: Front Neurosci. 2018 Mar 14;12:149. doi: 10.3389/fnins.2018.00149 (PMC5861187; doi:10.3389/fnins.2018.00149)
Supplement: Supplementary Datasheet 1 — Supplementary references. [file DataSheet1.docx]

1. Matsui, H., and Murata, M. (1997). Study of the perceived competence scale for adolescence. *Jpn. J. Educ. Psychol.*, 45, 220-227.
2. Yamagata, S., Takahashi, Y., Shigemasu, K., Ono, Y., and Kijima, N. (2005). Development and Validation of Japanese Version of Effortful Control Scale for Adults. *Jpn. J. Pers.*, 14(1), 30-41.
3. Miyashita, K. (1987). An examination of the Japanese version of Rasmussen’s ego identity scale. *Jpn. J. Educ. Psychol.*, 35, 253-258.
4. Kamide, H., and Daibo, I. (2005). Development of a Japanese version of the BIS/BAS scale. *Jpn. J. Interpers. Soc. Psychol.*, 5, 49-58.
5. Uchida, T., and Ueno T. (2010). Reliability and validity of the Rosenberg Self Esteem Scale: using the Japanese version of the RSES by Mimura & Griffiths (2007). *Annu. Bull., Grad. School Educ., Tohoku Univ.*, 58(2), 257-266.
6. Sakurai, S., and Sakurai, T. (1991). Construction of Japanese Version of Shyness Scale for College Students. *Bull. Nara Univ. Educ.*, 40(1), 235-243.
7. Kikuchi, A. (2004). Notes on the Researches Using KiSS-18. *Bull. Fac. Soc. Welfare, Iwate Prefectural Univ.*, 6(2), 41-51.
8. Sugawara, K. (1984). An attempting to construct the self-consciousness scale for Japanese. *The Jpn. J. Psychol.*, 55(3), 184-188.
9. Tokunaga, Y., and Horiuchi, T. (2012) Development of a Japanese Version of the Self-Concept Clarity (SCC) Scale. *Jpn. J. Pers.*, 20(3), 193-203.
10. Ochiai, T., and Oguchi, T. (2013). Development of a Japanese version of the TALE Scale. *Jpn. J. Psychol.*, 84(5), 508-514.
11. Hata, U., and Onodera, A. (2013). Development and Validation of a Japanese Version of the Ego-Resiliency Scale (ER89). *Jpn. J. Pers.*, 22, 1, 37-47.
12. Sato, H., Takahashi, F., Matsuo, M., Sakai, M., Shimada, H., Chen, J., et al. (2006). Development of the Japanese version of the social problem-solving inventory-revised and examination of its reliability and validity. Jpn. J. Behav. Ther. 32, 15-30.
13. Uchida, T., Kawamura, C., Mifune, N., Hamaie, Y., Matsumoto, K., Ambo, H., et al. (2012) The Japanese Version of the Brief Core Schema Scale for Schemata Concerning the Self and others: Identification of Schema Patterns and Relationship with Depression. *Jpn. J. Pers.*, 20(3), 143-154.
14. Tokuyoshi, Y., and Iwasaki, S. (2015). Development and psychometric evaluation of a Japanese version of the Personal Growth Initiative Scale-II. *Jpn. J. Psychol.*, 85, 2, 178-187.
15. Shimai, S., Otake, K., Utsuki, N., ikemi, A., and Lyubomirsky, S. (2004). Development of a Japanese version of the Subjective Happiness Scale (SHS), and examination of its validity and reliability. *Jpn. J. Public Health*, 51, 10, 845-853.
16. Kadono, Z. (1994). Attempt-The Satisfaction with Life Scale [SWLS]—of Japanese creating satisfaction measure for life. *Annu. Con. Jpn. Assoc. Educ. Psychol.*, 36, 192.
17. Shimizu, H., and Imae, K. (1981). Development of the Japanese edition of the Spielberger State-Trait Anxiety Inventory (STAI) for student use. *Jpn. J. Educ. Psychol.*, 29, 348–353.
18. Ito, S., Obu, S., Ota, M., Takao, T., and Sakamoto, S. (2008). Reliability and validity of the Japanese version of schizotypal personality questionnaire brief. *Jpn. J. Social. Psychiatry*, 17, 158-176.
19. Wakabayashi, A., Tojo, Y., Baron-Cohen, S., and Wheelwright, S. (2004). The Autism-Spectrum Quotient (AQ) Japanese version: Evidence from high-functioning clinical group and normal adults. *Jpn. J. Psychol.*, 75, 1, 78-84.
20. Uchiyama, K., Shimai, T., Utsuki, N., and Otake, K. (2001). EQS Manual. Tokyo, Japan: Jitsumu kyoiku Shuppan, Practical Education Press.
21. Fujita, K., Maekawa, H., Dairoku, H., and Yamanaka, K. (2006). *Japanese Wechsler Adult Intelligence Scale–Third Edition.* Tokyo, Japan: Nihon Bunka Kagakusha.
